# Supplementary material for: Dual-target peripheral and central magnetic stimulation for rehabilitation of chronic pelvic pain syndrome associated with psychosomatic symptoms: Study protocol for a randomized controlled trial
Source: PLoS One. 2025 Jul 17;20(7):e0326740. doi: 10.1371/journal.pone.0326740 (PMC12270166; doi:10.1371/journal.pone.0326740)
Supplement: S2 File — (DOCX) [file pone.0326740.s002.docx]

**Table 5 Randomization Application Form**

Application Date: Year Month Day

Random Number:

| **General Information** | | | |
| --- | --- | --- | --- |
| Name Abbreviation: | | Age: years | Gender: Male □ Female □ |
| Weight： Kg | | Height： m | BMI Index： Kg/m2 |
| DMS-5 Diagnosis | Generalized Anxiety Disorder Severity: Mild □, Moderate □, Severe □ | | |
|  | Severity of Major Depression: Mild □, Moderate □, Severe □ | | |

| Selection Criteria Checklist | | |
| --- | --- | --- |
| Inclusion criteria | 是 | 否 |
| 1.Meeting the CPPS diagnostic criteria in the 2022 EAU guidelines | □ | □ |
| 2. Definitive generalized anxiety disorder or major depressive disorder | □ | □ |
| 3. Individuals aged between 18 and 70 years | □ | □ |
| 4.No identifiable pathological changes in physical examinations and auxiliary tests | □ | □ |
| 5.No treatment other than oral medications in the 3 months prior to the visit | □ | □ |
| 6.Patient's informed consent and voluntary participation in the study | □ | □ |
| All of the above criteria must be "yes," otherwise the patient cannot be enrolled. | | |

| Exclusion Criteria Checklist | | |
| --- | --- | --- |
| Exclusion criteria | Yes | No |
| 1. Patients in the acute phase of systemic and intracranial hemorrhagic diseases | □ | □ |
| 2.Individuals with serious underlying conditions, such as cardiovascular, liver, kidney, respiratory, and blood disorders, in addition to malignant tumors and other advancing illnesses | □ | □ |
| 3.Patients with cardiac metal membranes, cardiac pacemakers, intracranial metal implants, lumbar sacral metal implants, and implantable electronic devices | □ | □ |
| 4.Individuals with infections in the head or lumbar sacral regions | □ | □ |
| 5.Individuals exhibiting unstable vital signs | □ | □ |
| 6.Patients with previous adverse reactions to magnetic therapy | □ | □ |
| 7.Individuals with atypical autonomic reflexes | □ | □ |
| 8. Patients with cognitive impairment who cannot cooperate | □ | □ |
| 9. Expectant or breastfeeding women | □ | □ |
| 10. Patients with a history diseases causing peripheral nerve damage | □ | □ |
| 11. Patients with debilitating diseases, such as malignant effusion, active pulmonary tuberculosis, cancer, or myasthenia gravis | □ | □ |
| 12.Patients with severe mental illness or epilepsy | □ | □ |
| All of the above criteria must be "no"; otherwise, the patient cannot be enrolled. | | |

Is it eligible for enrollment: Yes □ No □

Reason for not enrolling:

Randomization results for eligible participants:

Group A (dual-target magnetic stimulation) □

Group B (peripheral magnetic stimulation) □

Group C (sham stimulation) □
