# Supplementary material for: Dual-target peripheral and central magnetic stimulation for rehabilitation of chronic pelvic pain syndrome associated with psychosomatic symptoms: Study protocol for a randomized controlled trial
Source: PLoS One. 2025 Jul 17;20(7):e0326740. doi: 10.1371/journal.pone.0326740 (PMC12270166; doi:10.1371/journal.pone.0326740)
Supplement: S3 File — (DOCX) [file pone.0326740.s003.docx]

**成都肛肠专科医院临床研究**

**申请书**

**申报类型：** **[** **]大型队列研究** **[** **]重点项目** **[** **√]一般项目**

**项目名称：**外周联合中枢双靶磁刺激促进伴有精神心理障碍的慢性盆腔疼痛综合征康复：随机对照试验方案

**申请者：** 骆春梅

**所在科室：** 便秘科

**联系电话：** 18708102327

**电子信箱：** luochunmei@stu.cdutcm.edu.cn

**版本号：** 2.0

**版本日期：** 2023.10.20

成都肛肠专科医院临床研究中心

**一、科室意见**

| **申请者承诺**  我保证本申请书填报内容的真实性。如果获得资助，我与本项目组成员将严格遵 守《成都肛肠专科医院临床研究管理办法（试行）》的有关规定，切实保证研究 工作时间，按计划认真开展研究工作，按时报送有关材料。  申请者（签章）  年 月 日 |
| --- |
| **科室意见**  科主任（签章）：  年 月 日 |

**二、基本信息**

| **申** **请** **人** **信** **息** | **姓** **名** | | 骆春梅 | **性别** | 女 | **出生年月** | 1986 年 2月 | | **民族** | 汉族 |
| --- | --- | --- | --- | --- | --- | --- | --- | --- | --- | --- |
|  | **职** **称** | | 副主任医师 | | | **所在科室** | 便秘科 | | | |
|  | **学** **位** | | 硕士（在读博士） | | | **授予年份** | 2014 年 | | | |
|  | **电** **话** | | 18708102327 | | | **电子邮箱** | luochunmei@stu.tcm.edu.cn | | | |
|  | **主要研究** **领域** | | 盆底功能障碍的基础与临床研究 | | | | | | | |
| **项** **目** **基** **本** **信** **息** | **项目名称** | | 外周联合中枢双靶磁刺激促进伴有精神心理障碍的慢性盆腔疼痛综合征康复：随机对照试验方案 | | | | | | | |
|  | **研究类型** | | [ √]临床试验（随机对照试验） [ ]病例对照研究  [ ]队列研究 [ ]横断面研究 [ ]诊断性研究 [ ]其它  （请在最符合的一个研究类型前打“ √”) | | | | | | | |
|  | **研究年限** | | 2024年 1 月至 2025 年 12月 | | | | | **申请经费** | 5 万元 | |
| **摘** **要**  ( 500 字 以 内 ) | 慢性盆腔疼痛综合征（CPPS）发病率较高，可能表现出严重症状，导致心理健康问题，引起焦虑、抑郁和疼痛灾难化，并给家庭和社会带来巨大的压力。CPPS的确切原因和机制仍不清楚；研究显示，它可能是由精神心理问题及多系统功能障碍导致。因此，在CPPS治疗中解决精神心理问题至关重要。重复外周磁刺激（rPMS）在治疗CPPS方面具有潜在的有效性，而重复经颅磁刺激（rTMS）已经证明对焦虑和抑郁有治疗效果。本研究采用随机、双盲方法，招募66名合并有精神心理问题的CPPS参与者，并按性别分层随机分配到三组（1:1:1）：双靶位磁刺激组、外周磁刺激组和假刺激组，接受相应治疗。通过比较治疗前后的盆底疼痛评分（女性）或国家卫生研究院慢性前列腺炎症状指数（NIH-CPSI，男性），抑郁、焦虑和压力量表（DASS-21），盆底肌表面肌电图，阴部神经运动诱发电位和36项生活质量量表（SF-36）评分的变化，评估双靶磁刺激在伴有精神心理障碍的CPPS患者中的疗效和安全性。 | | | | | | | | | |
| **关键词** | | 重复经颅磁刺激，重复周围磁刺激，慢性盆腔疼痛综合征，随机对照试验 | | | | | | | | |

**三、项目组人员（含主要研究者）**

| **姓名** | **单位** | **学位** | **职称** | **项目任务** **分工** | **伦理培训** | **GCP** **培训** | **签名** |
| --- | --- | --- | --- | --- | --- | --- | --- |
| 骆春梅 | 成都肛肠专科医院 | 在读博士 | 副主任医师 | 课题负责 人 | 有 | 有 |  |
| 杨向东 | 成都肛肠专科医院 | 博士 | 教授 | 项目指导 | 有 | 有 |  |
| 常德贵 | 成都中医药大学附属医院 | 博士 | 主任医师 | 项目实施的督导 | 有 | 有 |  |
| 贺佳蓓 | 四川省第五人民医院 | 硕士 | 主治医 师 | 数据整理、 统计分析 | 有 | 有 |  |
| 蓝海波 | 成都肛肠专科医院 | 硕士 | 副主任医 师 | 项目实施、 病例的观 察，随访 | 无 | 有 |  |
| 赵美珠 | 成都肛肠专科医院 | 硕士 | 副主任医 师 | 项目数据 的收集、分 析 | 无 | 无 |  |
| 甄晓彬 | 成都肛肠专科医院 | 本科 | 主治医师 | 病例随访、 记录 | 无 | 无 |  |
| 刘忍 | 成都肛肠专科医院 | 硕士 | 主治医师 | 病例随访、 记录 | 无 | 无 |  |
| 白兰金 | 成都肛肠专科医院 | 硕士 | 住院医师 | 项目实施 | 无 | 无 |  |
| 李雪倩 | 成都肛肠专科医院 | 本科 | 住院医师 | 项目实施 | 无 | 无 |  |
| 田偲义 | 成都肛肠专科医院 | 本科 | 主管护师 | 病例的观 察，随访 | 无 | 无 |  |

项目秘书： 李雪倩 联系方式：19980717169 邮箱：469243152@qq.com

**四、申请者工作积累**

| 申请人临床工作中有丰富的经验积累，本项目开展所需的相关技术储备充分：  1.本人自 2011 年起一直从事于肛肠专业至今已有 10 余年，临床相关专业经验丰富。现任中华便秘医学会副秘书长，四川省预防医学会盆底疾病防分会委员会秘书，四川省女子医师协会肛肠专委会委员，中国肛肠病学研究生联合会理事等多个专业学会任职。  2.本项目开展所需的相关技术储备充分：2014年硕士毕业后便确定以盆底功能障碍等临床研究作为专业研究方向，长期从事慢性顽固性便秘的相关研究，擅长慢性顽固性便秘、顽固性肛门坠胀、肛门直肠疼痛、慢性前列腺痛、大便失禁、膀胱过度活跃等疑难疾病的诊治。并先后承担了市级科研项目“顽固性肛门坠胀与疼痛证治规律研究”和院级科研项目“胃肠肛门病合并精神心理疾病的证治规律研究”的研究。 |
| --- |

**五、创新性和可行性分析**

| 1.本项目特色及创新性  本研究采用前瞻性随机对照实验，设置对照组实验，将采用假刺激和采用外周磁刺激的CPPS患者作为对照组，采用双靶磁刺激的CPPS患者作为试验组，通过对比三组患者的临床相关指标安全性及有效性，探讨双靶磁刺激在合并精神心理障碍的CPPS患者中的治疗效果以及安全性。双靶磁刺激是笔者团队根据多年的临床经验以及深入了解各种CPPS治疗方法的基础上所得出的，目前在国内外文献报道中均未发现针对合并有精神心理问题的CPPS患者采用同类型治疗的相关研究。  2.可行性分析（科室条件、人员配备、病例来源、技术条件等）  成都肛肠专科医院是一家三级甲等专科医院，是集医疗、教学、科研、预防保健为一体的医学中心；其肛肠科是国家重点临床专科，便秘科及盆底中心在中国西部地区具有极高的影响力。该临床研究的治疗将在医院的盆底中心进行。  本团队前期采用外周磁刺激在CPPS治疗中的应用效果以及安全性的研究中已积累一定的临床经验，采用经颅磁刺激治疗焦虑、抑郁等精神障碍已具备标准化的流程。根据相关报道指出，CPPS在国内外的发病率均显现出上升趋势，合并精神心理问题是其疗效不佳的主要原因，因此，寻找针对合并精神心理障碍的CPPS患者的有效治疗方式显得尤为重要。  本研究团队目前拥有高级职称技术人员 5 人，硕士及以上学历8人，具有丰富的临床研究、患者随访及统计分析经验，能保证临床数据记录以及统计分析的准确性，人才梯度合理，分工明确， 能保证本课题的顺利完成。 |
| --- |

**六、进度计划和预期目标**

| 1.进度计划（包括各年度任务目标、考核指标及研究开发内容完成的计划进度。按年度进 行填写，每年度的研究计划和目标应明确、可考核，并能与课题整体研究任务、完成时间、 考核指标相对应。）  2024.01-2025.01 ①成功入组 40 例 CPPS患者（进行随机化分组，给予对应治疗），收集患者相关临床指标及术后并发症发生情况，并对数据进行系统记录。  2025.02-2025.07 ②成功入组2 0 例 CPPS患者（进行随机化分组，给予对应治疗），收集患者相关临床指标及术后并发症发生情况，并对数据进行系统记录。  2025.08-2025.10 ③将所收集的数据进行统计学分析。  2025.11-2025.12 ④攥写论文。  2. 预期目标与考核指标（重点说明本研究完成的最终成果和载体形式，如论文、成果、专利等；考核指标内容需完整、明确，能够考查课题完成的程度和实际效果。）  （1）探究双靶磁刺激在CPPS患者治疗中的应用效果及安全性。  （2）发表与临床研究相关的 SCI 文章 2 篇。 |
| --- |

七、经费预算

| **序** **号** | **预** **算** **科** **目** **名** **称** | **经费预算（万）** | **备** **注**  **（计算依据与说明）** |
| --- | --- | --- | --- |
| 1 | 临床研究业务费 | 3 | 计划书、CRF 表的制定费，研究 助理及研究者培训费，研究生劳 务费、外出学习、文献检索等费 用。 |
| 2 | 咨询费、调研费和劳务费 | 1 | 专家咨询费、论文发表，润色、 入组病例调查随访费等 |
| 3 | 资料费 | 0.3 | 复印打印费，资料统计分析费 等。 |
| 4 | 协作费 | 0.5 | 课题实施过程中需要协作或调 研所支付的费用。 |
| 5 | 交通费 | 0.2 | 外出学习交通费 |
|  | **合计** | 5 |  |

**外周联合中枢双靶磁刺激促进伴有精神心理障碍的慢性盆腔疼痛综合征康复：随机对照试验方案**

**研究方案**

**研究负责人：**骆春梅

**申办方：**成都肛肠专科医院

**版本号：**2.0

**版本日期：**2023-10-20

**目录**

[方案摘要](#_Toc18536) 3

[1. 立题依据 6](#_Toc21208)

[参考文献： 7](#_Toc6323)

[2. 研究目的 9](#_Toc10318)

[3. 研究对象 9](#_Toc25580)

[3.1 入选标准 9](#_Toc2035)

[3.2 排除标准 10](#_Toc5291)

[3.3 退出标准 10](#_Toc19402)

[4. 方案设计 10](#_Toc30603)

[5. 样本量估算及分组 10](#_Toc1563)

[5.1 样本量估算 11](#_Toc21861)

[5.2 患者编号和随机化分组 11](#_Toc12384)

[6. 研究步骤或治疗方案 11](#_Toc17164)

[6.1 临床干预措施和步骤。 11](#_Toc17021)

[6.2 双靶磁刺激治疗程序 12](#_Toc25465)

[6.3 外周磁刺激组治疗程序 12](#_Toc4490)

[6.4 假刺激组治疗程序 12](#_Toc18302)

[6.5 治疗方案 12](#_Toc19356)

[7. 随访方案 12](#_Toc23040)

[8. 研究结果的评价指标 12](#_Toc30858)

[8.1 主要疗效指标 12](#_Toc12794)

[8.2 次要疗效指标 13](#_Toc12279)

[8.3 安全性指标 13](#_Toc15048)

[9. 安全性评价 13](#_Toc16620)

[9.1 获益 13](#_Toc23531)

[9.2 风险 14](#_Toc24344)

[9.3 风险防范措施 14](#_Toc961)

[10. 招募过程 2](#_Toc8910)0

[10.1 招募程序 20](#_Toc8745)

[10.2 启动时间 21](#_Toc30320)

[10.3 招募材料 21](#_Toc16219)

[10.4 预期招募人数 21](#_Toc355)

[10.5 研究对象参加研究的时间 21](#_Toc28005)

[11 . 数据管理与统计分析计划 21](#_Toc14785)

[11.1 数据处理 21](#_Toc28533)

[11.2 统计分析 22](#_Toc23087)

[12 .资料保密计划 23](#_Toc8692)

[13. 伦理要求与知情同意 23](#_Toc28794)

[13.1 取得伦理委员会批件 23](#_Toc6746)

[13.2 知情同意 23](#_Toc1943)

[13.3 知情同意书的修订 23](#_Toc15801)

[13.4 受试者的隐私保护 23](#_Toc9845)

[13.5 研究方案的修改 24](#_Toc31942)

[14 . 质量控制和质量保证 24](#_Toc29300)

[14.1 数据管理 24](#_Toc26869)

[14.2 本研究的质量控制 24](#_Toc31362)

[14.3 原始资料的保存 24](#_Toc13216)

[14.4 试验盲法的设置 24](#_Toc6183)

[15. 数据和记录的保存 25](#_Toc28507)

[16. 研究流程 25](#_Toc32276)

[17. 总结报告和论文发表 25](#_Toc27867)

[附录1 26](#_Toc6167)

附录2 [2](#_Toc15674)7

[附录3 2](#_Toc8090)8

[附录 4 2](#_Toc19027)9

[附录5 3](#_Toc6167)0

附录6 [3](#_Toc15674)1

[附录7 34](#_Toc8090)

**方案摘要**

| **研究名称** | 外周联合中枢双靶磁刺激促进伴有精神心理障碍的慢性盆腔疼痛综合征康复：随机对照试验方案 |
| --- | --- |
| **主要研究者** | 骆春梅副主任医师 |
| **研究目的** | 本研究旨通过重复外周磁刺激（rPMS）联合重复经颅磁刺激（rTMS）对伴有精神心理障碍的盆腔疼痛综合征患者治疗的有效性和安全性，通过评估双靶磁刺激治疗对患者盆腔疼痛量表/NIH-CPSI评分表、抑郁焦虑和压力（DASS-21）量表及SF-36生活质量量表评估和盆底肌表面肌电图及阴部神经运动诱发电位的影响，以及对，比较双靶磁刺激与外周磁刺激对伴有精神心理障碍慢性盆腔疼痛综合征患者疼痛程度、精神心理及生活质量的影响；为临床治疗提供方案选择的依据。 |
| **主要评价指标** **（疗效指标）** | 盆腔疼痛评分表（女性）/NIH-CPSI（男性） |
| **次要评价指标** **（疗效指标）** | 盆底肌表面肌电  阴部神经运动诱发电位  抑郁焦虑和压力量表（DASS-21）  SF-36生活质量量表 |
| **安全性指标** | 血常规  肝肾功  心电图 |
| **入选标准** | （1）符合欧洲泌尿外科协会（European Association of Urology，EAU）慢性盆腔疼痛指南（2022版）CPPS诊断标准。  （2）年龄：18-70岁；  （3）符合DSM-5标准的广泛性焦虑障碍或重性抑郁症；  （4）体格检查和辅助检查未见明显病理变化；  （5）在就诊前3个月内，除口服药物外没有其他治疗；  （6）患者知情同意并自愿参与研究。 |

| **排除标准** | (1) 急性全身和颅内出血性疾病患者。  (2) 患有严重基础疾病的个体，如心血管、肝脏、肾脏、呼吸系统和血液疾病，以及恶性肿瘤和其他进展性疾病。  (3) 患有心脏金属膜、心脏起搏器、颅内金属植入物、腰椎金属植入物和可植入电子设备的患者。  (4) 在头部或腰骶区域感染的个体。  (5) 表现出生命体征不稳定的个体。  (6) 对磁疗有过不良反应的患者。  (7) 具有非典型自主神经反射的个体。  (8) 无法配合的认知障碍患者。  (9) 孕妇或哺乳期妇女。  (10) 患有导致外周神经损伤的疾病史的患者。  (11) 患有严重疾病的患者，如恶性渗出、活动性肺结核、癌症或重症肌无力。  (12) 患有严重精神疾病或癫痫的患者。 |
| --- | --- |
| **退出标准** | 参与者将在以下情况下被要求退出RCT：  （1）如果患者不遵守规定的治疗计划或接受替代治疗；  （2）如果患者出现严重不良反应或病情变化，导致他们无法继续参与试验；  （3）如果患者出现重要器官功能障碍、血压不稳、心率异常或呼吸困难；  （4）如果患者要求撤回知情同意，因为不可忍受的不良反应或无特定原因；  （5）如果患者在治疗或随访期间失去了随访。 |
| **受试者数量样本量验证** | 根据Yang Jing-ming及其同事的研究，在β=0.1和α=0.05的条件下，效应大小为0.5。疼痛评分将作为主要测量标准，并采用G*Power3.1.9.7估算样本量。经计算，研究队列中约有54名患者，考虑到10%的脱失率，每组将包括22人，该RCT需要66名参与者。 |

| **治疗方案** | 1. 双靶磁刺激组治疗操：①治疗前：三查七对，由从业资格师进行风险评估，包括患者的精神症状、药物治疗效果和禁忌症等。②治疗时：确定刺激方案：个体化刺激强度，首次治疗时测定静息运动阈值（   restmotor threshold，RMT），静息运动阈值确定患者磁刺激强度，rTMS刺激强度为120%RMT。辅助定位装置：根据定位帽，确定刺激皮层的位置;抑郁患者选择左侧DLPFC进行10Hz的高频刺激，焦虑患者选择右侧DLPFC进行1Hz的低频刺激。将座椅放平调整到水平10-20°，患者俯卧于座椅上，圆形线圈中心对准骶3区，刺激时肛周肌肉有收缩感，还有跖屈反应说明位置正确，先进行rPMS后再将座椅调至坐位进行rTMS。③治疗结束后：询问患者有何不适反应，老年患者离开前在床边稍坐片刻。医生完整填写各类记录单，保存备用；并行治疗室消毒隔离。  （2）外周磁刺激组：①治疗前：同双靶磁刺激组。②治疗时：仅进行rPMS,操作同双靶磁刺激组。③治疗结束后：同双靶磁刺激组。  （3）假刺激组：①治疗前：同双靶磁刺激组。②治疗时：采用不产生磁场的线圈，仅发出声音，先经骶3区假外周磁刺激再行假经颅磁刺激，操作同双靶磁刺激组。③治疗结束后：同双靶磁刺激组。  （4）疗程规定：20次为一疗程，每周5次，每天治疗1次；患者药物种类和剂量有明显调整时，需要主动告知治疗师。  （5）紧急情况处理：系统应按GB9706.1-2007中22.7要求提供防护措施，如紧急制动装置。在治疗过程中，如果出现意外抽描、意识丧失或其他紧急情况时，立即按照以下措施进行处理和急救：①立即停止rTMS治疗；②保护患者气道通畅；③密切监测忠者生命体征；④心脏停跳或呼吸骤停者，立即进行人工心肺复苏；⑤联系患者家属，告知情形和相应处理办法。紧急情况处理结束后，总结经验和教训。 |
| --- | --- |

**1.** **立题依据**

慢性盆腔疼痛综合征（CPPS）是指在没有证实的感染或其他明显的局部病理学原因可以解释疼痛的情况下，盆腔相关区域出现的持续或反复出现超过三个月的疼痛。它通常与消极的认知、行为、性和情感后果，以及提示下尿路（LUT）、性、肠、盆底或妇科功能障碍的症状有关^[1]^。男性中，CPPS在全球范围内的流行率在2%-16%之间^[2]^；而全球女性CPPs患病率高达24%^[3]^。慢性盆腔疼痛综合征等一系列的盆底功能性疾病其多样而难以启齿的症状会严重影响患者的社交活动及生活质量，并引起自卑及抑郁等精神问题，从而增加家庭乃至社会的压力^[4]^。一项统计发现每年治疗CPPS的费用估计约为8.8亿美元^[5]^。

CPPs确切的病因及发病机制目前仍不清楚，可能是精神心理障碍及多系统功能障碍相互作用的最终结果^[6]^。有研究认为慢性盆腔疼痛综合征作为盆底功能障碍的一种表现，它的发生发展与盆底神经肌肉损伤有关^[7]^.85%患者的慢性痛综合征起源于肌肉^[8]^。肌肉过度活动是慢性盆底痛综合征的特征，也是导致疼痛的主要原因^[9]^。CPPS的症状会严重影响患者的心理状况，带来焦虑、抑郁、疼痛灾难化等心理问题^[10]^。心理社会因素不仅直接导致精神障碍的发生与发展，还影响到躯体疾病的转归。心理因素在持续性盆腔和泌尿生殖疼痛的维持中始终被认为是相关的^[11]^。联合国疼痛协会将慢性疼痛描述为“不愉快的感觉和情感经历”,需要心理-社会治疗。这种治疗模式认识到了身心调节对疼痛的影响^[12]^。

CPPS患者往往具有中枢和外周神经系统超敏性，疼痛调节功能失调，往往会加重疼痛^[13-18]^。目前基于盆腔疼痛的病因，多采用物理治疗、药物治疗和神经阻滞。对于难治性病例，中枢和周围神经系统可以使用神经调节^[19]^。两个系统评价已经评估了神经调节技术对CPPS的效果。两项研究都得出结论，神经调节可能在减轻疼痛和改善生活质量方面对CPPs患者有效^[20,21]^。磁刺激可以在深层神经结构中产生小电流，以非侵入性地激活神经结构（可以用于检测神经刺激，并作为一种非侵入性的临床工具来调节和治疗神经功能）。有研究报道了在局部肌肉骨骼损伤患者中，施加8000次脉冲磁刺激于疼痛部位可以减轻疼痛^[22]^。另有研究表明骶神经根的磁刺激可以显著改善耻骨神经痛和坐骨神经痛^[23]^。一项研究表明，具有特定参数的磁刺激作用于盆骶区的周围神经可以抑制或激活神经通路，并调节异常的反射弧，从而影响尿道、膀胱、直肠和肛门、和盆底肌肉的功能^[24]^。经颅磁刺激由于其在调节脑功能方面的特异性以及它是一种无痛、安全可靠的治疗方法，磁刺激的使用持续快速增长。rTMS疗法在全球范围内应用于抑郁症患者，左侧DLPFC的HF-rTMS（使用局部8字型线圈或深H1线圈）具有明确的抗抑郁疗效^[25]^。美国食品和药物管理局（FDA）最早于2008年12月16日批准了这一应用；另一方面，在老年样本中，rTMS的益处已被报道对情绪有益^[26]^。右侧DLPFC的LF-rTMS(1 Hz-rTM)明显减轻广泛性焦虑，改善自我报告的情绪调节和睡眠质量^[27-29]^。

研究表明重复外周磁刺激（rPMS）可能有效治疗CPPS，重复经颅磁刺激（rTMS）对焦虑、抑郁等起到治疗作用。然而，外周磁刺激治疗CPPS的文章都是观察性研究或小样本研究，目前缺乏关于外周磁刺激治疗慢性盆腔疼痛综合征的高质量研究；此外，合并精神心理障碍也没有被考虑进去。因此，我们开展了这项研究，以评估rPMS联合rTMS在伴有精神心理障碍的CPPS患者中的疗效及安全性，并为治疗合并有精神心理障碍的CPPS患者提供更多选择。

**参考文献：**

[1] D. Engeler, A.P. Baranowski, B. Berghmans, J. Birch, J. Borovicka, A.M. Cottrell, P. Dinis-Oliveira, S. Elneil, J. Hughes, E.J. Messelink, R.A. Pinto, M.L.v. Poelgeest, V. Tidman, A.C.d.C. Williams, P. Abreu-Mendes, S. Dabestani, B. Parsons, J. Tornic, V. Zumstein, EAU Guidelines on Chronic Pelvic Pain, 2022.

[2] C. Smith, Male chronic pelvic pain: An update, Indian Journal of Urology 32(1) (2016).

[3] P. Latthe, M. Latthe, L. Say, M. Gülmezoglu, K.S. Khan, WHO systematic review of prevalence of chronic pelvic pain: a neglected reproductive health morbidity, BMC Public Health 6(1) (2006).

[4] J. Kwon, H.J. Lee, J.H. Joo, E.C. Park, Urinary incontinence status changes and depressive symptoms among middle-aged and older women: Using data from a survey of the Korean Longitudinal Study of Aging, Journal of affective disorders 279 (2021) 549-553.

[5] W. Stones, Y. Cheong, F.M. Howard, S. Singh, Interventions for treating chronic pelvic pain in women(Review), Cochrane Collab 11 (2010) 1-43.

[6] K. Grinberg, Y. Sela, R. Nissanholtz-Gannot, New Insights about Chronic Pelvic Pain Syndrome (CPPS), International Journal of Environmental Research and Public Health 17(3005) (2020).

[7] P. Enck, D.B. Vodusek, Electromyography of pelvic floor muscles, Journal of electromyography and kinesiology : official journal of the International Society of Electrophysiological Kinesiology 16(6) (2006) 568-77.

[8] A.J. Slomski, How groups successfully manage pain patients, Medical economics 73(1) (1996) 112, 115-6, 119-20 passim.

[9] G.A. Santoro, A.P. Wieczorek, C.I. Bartram, Pelvic Floor Disorders Imaging and Multidisciplinary Approach to Management, Springer-Verlag Milan, Berlin, Germany, 2010.

[10] D.A. Tripp, Managing psychosocial correlates of urologic chronic pelvic pain syndromes: Advice from a urology pain psychologist, Canadian Urological Association journal = Journal de l'Association des urologues du Canada 12(6 Suppl 3) (2018) S175-s157.

[11] C. Dybowski, B. Löwe, C. Brünahl, Predictors of pain, urinary symptoms and quality of life in patients with chronic pelvic pain syndrome (CPPS): A prospective 12-month follow-up study, Journal of psychosomatic research 112 (2018) 99-106.

[12] S.N. Raja, D.B. Carr, M. Cohen, N.B. Finnerup, H. Flor, S. Gibson, F.J. Keefe, J.S. Mogil, M. Ringkamp, K.A. Sluka, X.J. Song, B. Stevens, M.D. Sullivan, P.R. Tutelman, T. Ushida, K. Vader, The revised International Association for the Study of Pain definition of pain: concepts, challenges, and compromises, Pain 161(9) (2020) 1976-1982.

[13] L. Lowenstein, M.P. FitzGerald, K. Kenton, L. Hatchett, R. Durazo-Arvizu, E.R. Mueller, K. Goldman, L. Brubaker, Evaluation of urgency in women, with a validated Urgency, Severity and Impact Questionnaire (USIQ), Int Urogynecol J Pelvic Floor Dysfunct 20(3) (2009) 301-7.

[14] L. Lowenstein, Y. Vardi, M. Deutsch, M. Friedman, I. Gruenwald, M. Granot, E. Sprecher, D. Yarnitsky, Vulvar vestibulitis severity--assessment by sensory and pain testing modalities, Pain 107(1-2) (2004) 47-53.

[15] J.J. van Lankveld, M. Granot, W.C. Weijmar Schultz, Y.M. Binik, U. Wesselmann, C.F. Pukall, N. Bohm-Starke, C. Achtrari, Women's sexual pain disorders, The journal of sexual medicine 7(1 Pt 2) (2010) 615-31.

[16] C. Allaire, C. Williams, S. Bodmer-Roy, S. Zhu, K. Arion, K. Ambacher, J. Wu, A. Yosef, F. Wong, H. Noga, S. Britnell, H. Yager, M.A. Bedaiwy, A.Y. Albert, S. Lisonkova, P.J. Yong, Chronic pelvic pain in an interdisciplinary setting: 1-year prospective cohort, Am J Obstet Gynecol 218(1) (2018) 114.e1-114.e12.

[17] J. Thomtén, A. Karlsson, Psychological factors in genital pain: The role of fear-avoidance, pain catastrophizing and anxiety sensitivity among women living in Sweden, Scandinavian journal of pain 5(3) (2014) 193-199.

[18] K. Grinberg, I. Weissman-Fogel, L. Lowenstein, L. Abramov, M. Granot, How Does Myofascial Physical Therapy Attenuate Pain in Chronic Pelvic Pain Syndrome?, Pain research & management 2019 (2019) 6091257.

[19] C.B. Patel, A.A. Patel, S. Diwan, The Role of Neuromodulation in Chronic Pelvic Pain: A Review Article, Pain physician 25(4) (2022) E531-e542.

[20] A.M. Cottrell, M.P. Schneider, S. Goonewardene, Y. Yuan, A.P. Baranowski, D.S. Engeler, J. Borovicka, P. Dinis-Oliveira, S. Elneil, J. Hughes, B.J. Messelink, C.W.A.C. de, Benefits and Harms of Electrical Neuromodulation for Chronic Pelvic Pain: A Systematic Review, European urology focus 6(3) (2020) 559-571.

[21] M. Tutolo, E. Ammirati, J. Heesakkers, T.M. Kessler, K.M. Peters, T. Rashid, K.D. Sievert, M. Spinelli, G. Novara, F. Van der Aa, D. De Ridder, Efficacy and Safety of Sacral and Percutaneous Tibial Neuromodulation in Non-neurogenic Lower Urinary Tract Dysfunction and Chronic Pelvic Pain: A Systematic Review of the Literature, European urology 73(3) (2018) 406-418.

[22] J. Pujol, A. Pascual-Leone, C. Dolz, E. Delgado, J.L. Dolz, J. Aldomà, The effect of repetitive magnetic stimulation on localized musculoskeletal pain, Neuroreport 9(8) (1998) 1745-8.

[23] T. Sato, H. Nagai, Sacral magnetic stimulation for pain relief from pudendal neuralgia and sciatica, Dis Colon Rectum 45(2) (2002) 280-2.

[24] S. Wu, X. Sun, X. Liu, J. Li, X. Yang, Y. Bao, H. Yu, Clinical Observations of Percutaneous Tibial Nerve Stimulation Combined with Sacral Nerve Root Magnetic Stimulation for the Treatment of Male Chronic Pelvic Pain and Chronic Prostatitis, Arch Esp Urol 75(10) (2022) 813-818.

[25] J.P. Lefaucheur, A. Aleman, C. Baeken, D.H. Benninger, J. Brunelin, V. Di Lazzaro, S.R. Filipović, C. Grefkes, A. Hasan, F.C. Hummel, S.K. Jääskeläinen, B. Langguth, L. Leocani, A. Londero, R. Nardone, J.P. Nguyen, T. Nyffeler, A.J. Oliveira-Maia, A. Oliviero, F. Padberg, U. Palm, W. Paulus, E. Poulet, A. Quartarone, F. Rachid, I. Rektorová, S. Rossi, H. Sahlsten, M. Schecklmann, D. Szekely, U. Ziemann, Evidence-based guidelines on the therapeutic use of repetitive transcranial magnetic stimulation (rTMS): An update (2014-2018), Clin Neurophysiol 131(2) (2020) 474-528.

[26] A. Dardenne, C. Baeken, C.L. Crunelle, C. Bervoets, F. Matthys, S.C. Herremans, Accelerated HF-rTMS in the elderly depressed: A feasibility study, Brain Stimul 11(1) (2018) 247-248.

[27] G.J. Diefenbach, L.B. Bragdon, L. Zertuche, C.J. Hyatt, L.S. Hallion, D.F. Tolin, J.W. Goethe, M. Assaf, Repetitive transcranial magnetic stimulation for generalised anxiety disorder: a pilot randomised, double-blind, sham-controlled trial, The British journal of psychiatry : the journal of mental science 209(3) (2016) 222-8.

[28] G.J. Diefenbach, M. Assaf, J.W. Goethe, R. Gueorguieva, D.F. Tolin, Improvements in emotion regulation following repetitive transcranial magnetic stimulation for generalized anxiety disorder, Journal of anxiety disorders 43 (2016) 1-7.

[29] G.J. Diefenbach, L. Rabany, L.S. Hallion, D.F. Tolin, J.W. Goethe, R. Gueorguieva, L. Zertuche, M. Assaf, Sleep improvements and associations with default mode network functional connectivity following rTMS for generalized anxiety disorder, Brain Stimul 12(1) (2019) 184-186.

**2.** **研究目的**

本研究旨在评价重复外周磁刺激（rPMS）联合重复经颅磁刺激（rTMS）对伴有精神心理障碍的盆腔疼痛综合征患者治疗的有效性和安全性。通过评估干预措施对患者盆腔疼痛量表/NIH-CPSI评分表、抑郁焦虑和压力（DASS-21）量表及SF-36生活质量量表评分及患者盆底肌表面肌电图及阴部神经运动诱发电位的影响，比较双靶磁刺激和外周磁刺激对伴有精神心理障碍的慢性盆腔疼痛综合征患者疼痛程度、精神心理评分及生活质量的影响；为临床治疗提供方案选择的依据。

**3. 研究对象**

符合欧洲泌尿外科协会（European Association of Urology，EAU）慢性盆腔疼痛指南（2022版）CPPS诊断标准及符合DSM-5的广泛性焦虑障碍或重性抑郁症的患者。

**3.1 入选标准**

（1）符合欧洲泌尿外科协会（European Association of Urology，EAU）慢性盆腔疼痛指南（2022版）CPPS诊断标准。

（2）年龄：18-70岁；

（3）根据DSM-5标准可明确诊断为广泛性焦虑障碍或重性抑郁症；

（4）体格检查和辅助检查未见明显病理变化；

（5）在就诊前3个月内，除口服药物外没有其他治疗；

（6）患者知情同意并自愿参与研究。

**3.2 排除标准**

(1) 急性全身和颅内出血疾病患者。

(2) 患有严重基础疾病的个体，如心血管、肝脏、肾脏、呼吸系统和血液疾病，以及恶性肿瘤和其他进展性疾病。

(3) 患有心脏金属膜、心脏起搏器、颅内金属植入物、腰椎金属植入物和可植入电子设备的患者。

(4) 在头部或腰骶区域感染的个体。

(5) 表现出生命体征不稳定的个体。

(6) 对磁疗有过不良反应的患者。

(7) 具有非典型自主神经反射的个体。

(8) 无法配合的认知障碍患者。

(9) 孕妇或哺乳期妇女。

(10) 患有导致外周神经损伤的疾病史的患者。

(11) 患有严重疾病的患者，如恶性渗出、活动性肺结核、癌症或重症肌无力。

(12) 患有严重精神疾病或癫痫的患者。

**3.3 退出标准**

参与者将在以下情况下被要求退出RCT：

1. 如果患者不遵守规定的治疗计划或接受替代治疗；
2. 如果患者出现严重不良反应或病情变化，导致他们无法继续参与试验；
3. 如果患者出现重要器官功能障碍、血压不稳、心率异常或呼吸困难；
4. 如果患者要求撤回知情同意，因为不可忍受的不良反应或无特定原因；
5. 如果患者在治疗或随访期间失去了随访。

**4. 方案设计**

本研究采用前瞻性、随机、盲法、对照试验设计。

**5. 样本量估算及分组**

**5.1 样本量估算**

根据Yang Jing-ming及其同事的研究，在β=0.1和α=0.05的条件下，效应大小为0.5。疼痛评分将作为主要测量标准，并采用G*Power3.1.9.7估算样本量。经计算，研究队列中约有54名患者，考虑到10%的脱失率，每组将包括22人，该RCT需要66名参与者。

**5.2 患者编号和随机化分组**

将患者数据输入电脑来获得一个受试者编号，所有受试者编号将为 3 位数，患者在研究过程中始终使用该编号，且不会被重新分配，患者编号分配后撤回知情同意或中止研究的患者将会保留他们的初始编号。随机化使用分层区组随机化法进行分组，根据性别因素进行分层，然后再在层内进行区组随机化分组。由于本研究目的是要对比慢性盆腔疼痛的症状评分及焦虑、抑郁评分的改善情况，性别是重要的影响因素，因此将性别作为分层因素，使用区组随机（层内区组数目为 2，长度为4）的方法进行分组。实现上述分层区组随机化的分组可以通过SPSS软件进行。

**6. 研究步骤或治疗方案**

**6.1 临床干预措施和步骤。**

（1)治疗前：进行三次检查和七次比较。研究人员将进行风险评估。

（2)刺激计划：首先进行rPMS，然后进行rTMS。

（3)刺激部位：对于外周刺激，圆形线圈的中心将与骶3区域对齐。在刺激过程中，如果会感到会阴肌肉收缩和脚背屈反应，则表示位置正确。对于中央刺激，针对重性抑郁患者将瞄准左侧DLPFC（l-DLPFC）区域，对于广泛性焦虑障碍患者将瞄准右侧DLPFC（r-DLPFC）区域。

（4) 刺激强度：对于外周刺激，将应用最大刺激强度的50%。对于中央刺激，设置将调整为静息运动阈值[RMT]的120%。

（5)刺激参数：①外周刺激：20赫兹，2秒刺激，28秒间隔，共1600脉冲。②r-DLPFC：1赫兹，10秒刺激，2秒间隔，共1000脉冲。③l-DLPFC：10赫兹，4秒刺激，26秒间隔，共3000脉冲。

（6) 刺激结束：刺激线圈将从患者的刺激部位移开。患者将被告知治疗完成，并且评估者将询问是否有任何不适。如果患者躺着，他们将被指示逐渐坐起，然后站起以避免摔倒。

（7) 治疗过程：每天一次，每周5天，共4周（20次）。疗程在第20次治疗后结束。

**6.2 双靶磁刺激治疗程序**

在治疗过程中：确定刺激计划，并在中央刺激之前开始进行外周刺激。将座椅调整到水平角度为10-20°，指导患者俯卧在座椅上，将圆形线圈的中心与骶3区域对齐，刺激直到感觉到肛门括约肌收缩或脚背屈反应，表明位置正确。确认位置后，使用单脉冲刺激确定刺激强度，并根据计划进行重复外周磁刺激。调整座椅，让患者坐直，测量初始治疗期间的RMT以确定患者的磁刺激强度。rTMS刺激强度设置为RMT的120%。

辅助定位设备：根据定位帽确定受刺激的皮层位置；对于重性抑郁患者，刺激左侧DLPFC，对于广泛性焦虑障碍患者，刺激右侧DLPFC，遵循中央磁刺激计划。

**6.3 外周磁刺激组治疗程序**

按照与双靶点磁刺激组相同的程序实施 rPMS。所有参数均与双靶点磁刺激组相同。

**6.4 假刺激组治疗程序**

给予外周和中央磁刺激均使用一个能发出声音但不产生磁场的线圈。其余参数和程序与双靶磁刺激组相同。

**6.5 治疗方案**

一个治疗疗程将包括20次，每周五次，每天一次。第20次治疗结束时将标志着疗程的结束。随访将安排在疗程结束后8周。在研究期间，患者可能会接受口服药物治疗，具体药物和剂量必须在入组时记录在案。如果患者的药物类型和剂量有任何重大调整，他们需要主动告知治疗师。

**7.** **随访方案**

（1）评估、随访计划及内容：治疗前、每周治疗结束时及疗程结束8周，需返院评估一次。评估内容包括患者的盆腔疼痛评分/NIH-CPSI，DASS-21量表，SF-36评分量表，盆底表面肌电图，以及阴部神经运动诱发电位。

（2）建立完整数据库，采用由专人负责的随访机制。

（3）随访时间节点：治疗前，每周治疗结束时至疗程结束8周后。

（4）在院评估和返院随访的时间节点以外，由专门的随访医护人员采取电话、信件 问卷、电子邮件等形式进行跟踪随访。

8. **研究结果的评价指标**

**8.1 主要疗效指标：疼痛评分**

(1) 盆腔疼痛评分表（适用于女性）：包括患者的基本信息、详细的症状变化以及先前治疗的有效性。它以性活动和月经引起的疼痛加重和缓解作为医疗史询问的起点。补充细节包括与运动、尿液和肠道功能相关的症状。

(2) NIH-CPSI（男性）：主要由三部分组成，评估慢性前列腺炎引起的疼痛或不适、尿路症状以及对生活质量的影响，共有九个问题。它具有客观、方便的特点，患者很快接受，可为科研和临床工作提供重要参考。

**8.2 次要疗效指标:**

(1) 焦虑和抑郁评分（DASS-21）：关于抑郁，轻度、中度和重度抑郁的临界值分别为10、14和21；对于焦虑量表，轻度、中度和重度焦虑的临界值分别为8、10和15；对于压力量表，轻度、中度和重度压力的临界值分别为15、19和26。

(2) 盆底肌表面肌电图：参与者将仰卧位，上半身和下半身形成一定角度（约120°），脚自然向外旋转。会在直肠放置一个电极以收集盆底肌表面肌电图数据，同时使用腹部电极监测腹部肌肉活动。在检查之前，参与者将被指导排尿和排便，教会如何正确收缩和放松盆底肌，并了解评估过程。他们将学会如何快速收缩并保持10秒的收缩。参与者将根据语音提示收缩和放松盆底肌以记录表面肌电图数值。

(3) 会阴神经运动诱发电位：参与者将被指示使用甘油栓进行直肠排空，并采取俯卧位。磁刺激线圈将放置在S3平面中线的侧面3-5厘米处，以55-60%的最大输出强度同时刺激两侧。在直肠内放置一个表面电极以记录肛门括约肌，接地电极连接到手腕。放大器的采样带宽为5-2000赫兹，每个分度线的灵敏度为3毫秒，分析时间为30毫秒。将进行五次成功的记录，并取平均结果。

(4) SF-36 QoL量表：一个包含36个项目的通用定量量表，涵盖了包括身体功能、身体角色、身体疼痛、一般健康、活力、社交功能、情感角色和心理健康在内的八个领域。

术后发生不良事件按照不良事件报告表进行登记，如为严重不良事件，请填写严重不 良事件表，并请在24小时内报告临床试验负责人、伦理委员会。并填写SAE报告表。

**8.3 安全性指标**

血常规、肝肾功及心电图：检查治疗措施是否对参研者造成伤害。

**9. 安全性评价：**

**9.1 获益**

根据相关报道指出，慢性盆腔疼痛综合征在国内外均显现出较高的发病率，伴有精神心理障碍的患者治疗方案有限且疗效差，因此，寻找针对伴有精神心理障碍的慢性盆腔疼痛综合征新的有效治疗方式显得尤为重要。 目前，随着无创神经调控技术的发展，经颅磁刺激已被广泛用于精神、心理疾病的治疗，外周磁刺激也在疼痛治疗中显示出一定疗效。本团队根据临床经验及文献报道，采用双靶磁刺激治疗伴有精神心理障碍的慢性盆腔疼痛综合征患者，观察其临床有效性及安全性，希望能为更多的关注于盆腔疼痛综合征的医师提供治疗参考，给广大患者带来健康与希望。

**9.2 风险**

（1)如果在研究期间您出现任何不适，或病情发生新的变化，或任何意外情况，不管

是否与研究有关，均应及时通知您的医生，他/她将对此做出判断并给与适当的医疗处理。

（2)经颅磁刺激已被国内外多项指南、共识推荐用于焦虑正、抑郁症的治疗；回顾本团队既往已开展的外周磁刺激和经颅磁刺激，仅有极少数患者出现刺激部位疼痛、短暂性听力减退，无严重不良反应，已证实该试验安全有效，风险可控。

**9.3 风险防范措施**

**9.3.1 指导原则：**

（1）预防为主：发现病例，及时报告，积极采取有效措施控制病情发展。

（2）依法管理：在临床试验的全过程中，贯彻执行相关法律法规，对出现的突发事件和 不良事件，要及时报告，在整个控制和救治工作中实行依法管理，对于违法行为，依法追究法律责任。

（3）分级负责：在临床试验中，严格执行标准化操作规程，定期或不定期监查、督察整 个过程，做到及时发现，及时救治。

（4）快速反应：建立预警和医疗救治快速反应，强化人力、物力、财力储备，增强应急处理能力，按照早期发现，及时报告，依靠科学，措施果断的原则，及时准确处置。

**9.3.2 组织管理：**

（1）领导机构：在院长领导下由医务处具体负责组织实施突发事件的处理工作。负责监督本院医务人员的医疗服务工作，检查医务人员的执业情况、接受投诉并向其提供咨询服务。配合并协调与之相关的争议和处理。

（2）指挥体系：将临床试验中受试者损害及突发事件纳入医院抢救和正常的医疗纠纷处理工作中。

（3）日常管理工作：临床试验机构办公室为日常的业务管理部门，具体负责临床试验的业务指导，组织管理与质量控制，监督检查，并负责日常信息沟通与组织协调工作及突发事件的报告。

（4）参与人员：各专业科室医、护、技专业技术人员以及心理卫生人员。

**9.3.3 防范措施：**

（1）伦理委员会的保证：临床试验开始前，试验方案经伦理委员会审议同意并签署意见后方能实施；临床试验进行期间，试验方案的任何修改均需经伦理委员会批准后方能执行；试验中发生任何受试者损害及严重不良事件，需向伦理委员会报告。

（2）主要研究者的保证：临床研究项目负责人应具有本科以上学历和高级职称，有临床试验方案中所要求的专业知识和经验，熟悉申办者所提供的与临床试验有关的资料和文献，并有权支配进行该项试验所需要的人员和设备；具有处理不良事件的能力和及时上报事件 的责任心。

（3）研究者的保证：熟悉不良事件报告程序的标准操作规程；临床试验开始前，各抢救设备和急救药品及时到位，确保出现受试者损害及突发事件时，受试者在第一时间得到救治。

（4）对受试者的保证：受试者必须是自愿参加并且对研究项目有充分的了解；必须始终尊重受试者保护自身的权利；尽可能采取措施以尊重受试者的隐私、资料的保密，并将其身体和精神以及人格的影响减至最小；向受试者告知该项试验的各方面情况后，受试者自愿确认其同意参加临床试验的过程，须以签名和注明日期的知情同意书作为文件证明。

（5）机构的保证：建立健全质量保证体系；对主要研究者资格的保证；对各专业科室设施的保证；对临床试验方案的质量保证；对临床试验数据资料的质量控制；建立符合GCP 管理规范的工作制度、设计规范、标准操作规程。制定受试者损害和突发事件应急预案：成立受试者损害和突发事件处理小组，保证医疗过程中出现受试者损害及突发事件后，受试者或患者得到及时处理；做好受试者损害与突发事件预防、现场控制、应急处理及其他物资和技术的准备与协调调度。

**9.3.4 不良事件：**

不良事件（AE）是指在临床诊疗活动中以及医院运行过程中，任何可能影响病人的诊 疗结果、增加病人的痛苦和负担、并可能引发医疗纠纷或医疗事故，以及影响医疗工作的 正常运行和医务人员人身安全的因素和事件。该事件不一定与这种治疗有因果关系。因此，不良事件可以是任何不良的和非预期的体征（包括异常的实验室发现）和症状，或者是与 医学产品使用有时间上的相关性的疾病，无论是否认为与研究有关。

研究过程中出现的疾病进展或恶化（包括出现疼痛加重和因为疾病进展而引起的精神心理疾病加重）应作为疗效评价的一部分，不应报告为不良事件或严重不良事件。

在临床研究期间所发生的所有不良事件将在病例报告表（CRF）的不良事件页上报告。 不良事件的严重程度将使用按照中国《医疗质量管理办法》制定的《医疗质量安全不良事件分级分类标准》 (https://www.gov.cn/zhengce)进行分级并按照CRF上的要求详细报告。

**<9.3.4.1> 严重不良事件的定义**

严重不良事件(SAE)是指在治疗过程中出现的符合下列某一标准的任何不良医学情况：

（1）致命性(导致死亡；注意：死亡是后果，不是事件)；

（2）危及生命(注意：“危及生命”是指在事件发生时患者立即有死亡的危险，并不是指如果事件更严重一些的话会引起死亡这样的假设)；

（3）导致患者住院治疗或者住院时间延长；

（4）导致终身的或严重的残疾/功能缺陷；

（5）在医学上具有重要意义或需要干预措施以防止上述任何一种后果的发生。

**<9.3.4.2> 因果关系**

应使用下列标准评估不良事件与治疗的关系：

（1）很可能有关：该类别指以很高程度的确定性认为与临床研究相关的不良事件。如果具备以下标准，可认为一个不良事件“很可能有关”：

①不良事件的出现与干预措施具有合理的时间相关性。

②已知的患者疾病状态、环境或毒性因素或患者使用的其他治疗不能合理解释不良事 件。

（2）可能有关(必须有前两项)：该类别指与本次研究不太可能有关，但是又不能肯定排除关联性的不良事件。如果具备以下标准，可认为一个不良事件“可能相关”：

①不良事件的出现与干预措施具有合理的时间相关性。

②不良反应可能是由患者的疾病状态、环境或毒性因素或患者使用的其他合并治疗造 成的。

（3)可能无关(必须具有前两项)：该类别适用于满足下述标准的不良事件：

①不良事件的出现与干预措施不具有合理的时间相关性。

②不良事件明显由患者的疾病状态、环境或毒性因素或患者使用的其他合并治疗造成。

1. 无关：该类别指清楚明确地判断为仅由外部因素(疾病、环境等)引起，而且不符合“可能无关”、“可能有关”或“很可能有关”项下相关性判断标准的不良事件。

必须遵照ICH对临床试验安全性数据管理指南、快速报告的定义和标准进行。

**<9.3.4.3> 预期不良事件**

（1）癫痫发作；

（2）晕厥；

（4）局部疼痛、头痛、不适；

（5）短暂的听力变化；

（6）短暂的认知/神经心理学的变化；

（7）急性精神变化；

（8）其他。

**<9.3.4.4> 不良事件处理措施**

（1) 根据具体情况采取必要的治疗措施，决定是否中止临床试验。并将其症状体征或实验室检查结果、出现时间、持续时间、程度、处理措施、经过等详细记录于病案，评价其与临床试验的相关性，由研究者签名并注明日期。

（2) 发现不良事件时，研究者应立即处理并向项目负责人、伦理委员会及临床研究中心汇报，根据病情决定必要的诊断与治疗措施，决定是否中止临床试验。所有不良事件件都应追踪调查，详细记录处理经过及结果，直到妥善解决或病情稳定，若化验异常者应追踪至恢复正常。追踪随访方式可根据不良反应的轻重选择住院、门诊、家访、电话、通讯等方式。

（3) 不良事件的报告：在研究期间，不论患者接受哪种治疗，所发生的任何严重不良事件或是严重的实验室检查结果异常，研究者必须填写不良事件报告表，并在获悉后24小时内报告给项目负责人、伦理委员会及临床研究中心。

SAE报告联系人：李雪倩（成都肛肠专科医院盆底中心）

单位联系人：张羽（成都肛肠专科医院学术委员会）

手机：+86-13608015271

邮箱：1246649597@qq.com

地址：四川省成都市青羊区太升南路大墙东街152号

（4）记录：研究者应在原始病案和CRF表中记录受试者的症状、体征、实验室检查，损害出现的时间、持续时间、程度、处理措施和经过等，保证记录真实、准确、完整、及时、 合法，填写严重不良事件报告表，签名并注明日期；在原始记录中应记录时间、报告方式以及报告的机构。

（5）随访：研究者应对所有受试者损害进行随访，根据病情决定随访时间，在随访过程中给予必要的处理和治疗措施，以确保将受试者损害降至最低，充分保证受试者安全。详细记录随访的经过和处理的结果。

**9.3.5 突发事件处理措施：**

（1）突发公共卫生事件处理措施：

①报告：在正常工作日，当班医护人员应立即报告受试者损害突发事件处理小组，节假日或夜间应立即报告行政总值班，由总值班向受试者损害和突发事件处理小组报告，小组人员应综合评估，初步判断突发公共卫生事件的性质，并向上级行政部门报告，提出是否 启动突发公共卫生事件应急预案的建议。

②启动应急预案：经上级卫生行政部门批准后，启动突发公共卫生事件应急预案，包括： ★成立特殊门诊、急诊及留诊室。

★院领导值班：院办迅速制定院领导值班表，保证一名院领导24小时值班，全面负责突发事 件；医务处每日有专门人员24小时值班；所有相关人员24小时手机开通，确保联络畅通，

并于每日上午8：00 、下午5：00到院部汇总相关信息，集中讨论，处理有关问题。

★保障物质供应：紧急调集人员、储备相关物资、交通工具以及相关的设施、设备，确保 医疗资源的合理配置，保障重点科室的医疗应急物资供应。

★疏散或隔离：根据病情需要，必要时对人员进行疏散和隔离。

★救援与记录：医务人员严格遵守防护措施，对患者进行紧急医疗救护和现场救治；书写 详细、完整的病历记录；需要转送他院的，按照规定转送至接诊的或指定的医疗机构。

★培训和演练：平时针对突发公共卫生事件的性质进行应急处理相关知识、技能的培训和

演练，随时做好应急准备工作。 （2） 自然灾害的处理措施：

火灾、水灾和地震等发生应立即与“119”或“110”联系，并通知受试者损害和突发事件处理小组，节假日或夜间应立即报告总值班，由总值班通知上述小组。小组负责人立即向院长报告，接受指示，同时由院办向全院通告紧急状况。相关职能部门做好各自工作。

（3）紧急停电、停水处理措施：

处理措施：一旦由于局部线路的原因造成科室突然停电，医护人员应当立即电话通知总务科管理人员，后勤管理人员在接到通知后立即安排专业人员赶赴现场紧急抢修线路，尽快恢复供电。各科室提前接到停水、停电通知，务必提前安排好工作；避开该时间段进行的相关检查或治疗。对于临床试验方案中要求必须当天检测的标本，应确保标本质量的前提下妥善保存，待供水或供电恢复后再行检测。

**9.3.6 处罚**：医务人员有下列行为之一的，由医院有关部门责令改正、通报批评、给予警告；

对科室负责人和其他直接责任人依法给予降级或撤职的纪律处分；造成受试者（患者）致 残、死亡或疾病传播、流行或者对社会公众健康造成其他严重危害后果，构成犯罪的，依 法追究刑事责任。

（1）未按规定履行不良事件或突发事件报告职责，隐瞒、缓报或者谎报的；

（2）未按规定对受试者出现的不良事件或突发事件及时采取措施的；

（3）突发事件来临时拒绝接诊病人的；

（4）突发事件来临时拒不服从医院工作调度的。

**10. 招募过程**（包括招募程序及启动时间、招募材料、预期招募人数）

**10.1 招募程序：**包括招募受试者、筛选合格受试者、获得受试者的知情同意三个环节。

**10.1.1 招募受试者**

（1）受试者招募工作人员：负责接诊的医生、分管的住院医师、也可以是临床监管医生。招募人员确定后开始制定招募计划与需求。

（2）招募方式：第一种是招募人员确定该患者符合试验要求，推荐患者参加试验；第二种是患者的主治医生不是试验的研究者，确定患者符合临床试验要求，询问患者意愿后与研究者联系纳入患者。

（3）招募的场所：医院门诊，住院部或者健康体检中心。

（4）接待潜在受试者：设置专门的招募窗口由专人负责接待来访的受试者或者利用专线电话对咨询患者进行疑问解答。

（5）按纳入和排除标准以及医生的临床经验初步判断受试者入选可能性。

（6）受试者合格性筛查：对初步判断可能合格的受试者进行试验概况说明，进行体格检查或实验室检查，根据检查结果，再次确认受试者是否真正符合试验纳入排除标准。

（7）受试者知情同意：对筛查合格的患者进行临床试验方案的详细说明，并告知可能存在的获益和风险，做到充分知情。

**10.1.2 筛选合格受试者**

将对初步判断可能合格的受试者进行合格性判断，事先应当根据合格性标准，设计 好单独的、清晰明确的病历筛查表。合格性判断标准就是把临床试验的诊断标准、纳入排 除标准进行细化为可判断的条目，并根据疾病和试验设计情况排除特殊人群（如妊娠、传 染性疾病）和患有相关并发症的患者。通过合格性判断标准判断受试者是否符合临床试验 的要求，符合研究条件的患者进入临床试验，判断不合格的受试者予以剔除，不对其进行 入组。不符合要求的患者产生的额外判断费用应由研究项目承担，并给予常规治疗。具体 的合格性判断流程如下：①根据事先确定的诊断标准纳入符合标准的受试者；②再根据事 先确定的纳入标准纳入符合标准的受试者；③最后根据排除标准将具有排除标准特征的患 者排除纳入不具有排除标准特征的患者。

**10.1.3 获得受试者的知情**

通过合格性筛查后，研究人员应该对每一个受试者进行知情同意告知并确认签字。

**10.2 启动时间**

项目获得审批通过并通过伦理委员会批准后于2024年1月1日开始启动受试者招募。

**10.3 招募材料**

知情同意书。

**10.4 预期招募人数**

符合入选条件的66名伴有精神心理障碍的慢性盆腔疼痛综合征患者。

**10.5 研究对象参加研究的时间**（每次参与时间与总时间）

入组并已接受第一周治疗（5次）为研究对象第一次参加研究时间；研究对象每周接受5次治疗为一次参研时间，共计4周（20次）治疗；疗程结束后8周返院完善相关检查及评估；总的参加研究时间为疗程结束8周后结束。

**11 、数据管理与统计分析计划**

**11.1 数据处理**

**11.1.1 数据收集**

所有资料均应及时、如实、详细地纪录在病历登记表（Case Report Form，CRF）中。研究者必须按照方案要求将信息输入CRF，研究中心委派监督员将检查CRF的完整性和准确性，并指导研究中心人员进行必需的修改或补充。CRF由研究监督员送交数据处理，一份复件保留在研究中心，一份附件则作为监督员的工作附件。CRF将交与两位可靠的医学数据处理员进行数据录入与核实。病历记录表应由本单位指派专人填写并经单位该项目负责人签名后方能视为有效病例。临床试验结束后，按临床总结规范要求写出临床分总结报告。

**11.1.2 病例报告表（CRF）**

在临床试验中，需要将规定的观察或检查项目记录在病例报告表中。病例报告表中的

内容须与原始资料完全一致，对于由原始资料计算所得的结果，其计算的依据应可以溯源。

在填写时须符合以下标准：

（1）用黑色签字笔或黑色圆珠笔填写；

（2）已经签署知情同意书并符合入选标准的病例，即使没有进行治疗或治疗后判定为不符合入选标准，也应填写病例报告表；

（3）进行修正时，须用横线划去原记录(不能用修正液等修改)，但要保证原记录的可辨认， 并在更正处签名并签署更正日期。

（4）对于未查项目，须标注“ND(Not Done) ”。

**11.1.3 数据库管理与质量控制**

CRF内的数据项目将使用具有二次录入审核的复式输入法输入研究数据库。文本项目 （如注释）从CRF 输入一次之后只能手动核对。随后，数据管理人员使用从确认程序和数据库列表中打印的错误信息，对输入数据库的信息进行系统检查。如有必要，一个独立的文件对DQF（Data Quality Form）处理及归档进行试验特殊操作有明确说明（如确认计划）。数据库在宣告完成并且无误后将被锁定。在那以后任何对数据库的改动只能经由获得临床研究领导者、研究统计学家和数据管理者联合书面同意而实现。结果的判读由不知道患者分组情况的第三方进行，确保结果的客观性。

**11.2 统计分析**

**11.2.1 分析人群定义：**

入组并进行治疗的病人构成本次试验的意向性治疗人群(ITT intention-to-treat ,

population)和遵从本临床试验方案要求并完成本次研究的病人构成符合临床实验的方案人群(PP-Per Protocol)，统计分析对缺失数据将采用意向分析（ITT），比较“完全分析集，FAS”和“符合方案集，PP”。

**11.2.2 统计分析方法**

统计分析将使用SPSS 27.0软件进行。连续数据将以均值±标准差的形式呈现。配对t检验将用于组间比较，重复测量方差分析将用于组内比较，包括治疗前后和随访期间。统计学显著性将由P值<0.05表示。在试验期间如有人员减少，将进行意向分析（ITT），比较“完全分析集，FAS”和“符合方案集，PP”。

**12 、资料保密计划**

参加试验及在试验中的个人资料均属保密内容。可以识别受试者身份的信息将不会透露给研究小组以外的成员，除非获得受试者的许可。所有的研究成员和研究申办方都被要求对受试者的身份保密。受试者的档案将保存在有锁的档案柜中，仅供研究人员查阅。为确保研究按照规定进行，必要时，政府管理部门或伦理审查委员会的成员按规定可以在研究单位查阅受试者的个人资料。这项研究结果发表时，将不会披露受试者个人的任何资料。

**13 、伦理要求与知情同意**

**13.1 取得伦理委员会批件**

在研究机构启动临床试验之前，必须获得研究机构伦理委员会的批件。在试验进行中 研究者应按要求提交试验进度报告给伦理委员会，试验结束后还应该书面报告伦理委员会。

**13.2 知情同意**

在病例登记前，主要研究者或研究者按照知情同意书向受试者说明临床试验相关内容 等，随后给予受试者足够的时间考虑，并由受试者自主决定是否签署知情同意书。主要研 究者或研究者以及受试者在知情同意书上签名并注明日期（说明日期、获得知情同意日期）。若试验相关人员进行补充说明，则该试验相关人员也须签名或填写姓名并盖章并记录说明日期。另外，试验过程中若因试验方案发生变更，或获得有可能影响受试者继续参加试验的新信息而需修订知情同意书时，则需向受试者说明变更的内容，并由受试者再次自主决定是否签署新的知情同意书。如果受试者不同意签署新的知情同意书，则受试者须中止临床试验。知情同意书一式两份，原件由中心保存、复印件由受试者保留。

**13.3 知情同意书的修订**

主要研究者可以依据最新的信息考虑修改知情同意书，新修订的知情同意书必须经过伦理委员会的审批同意后，才能在临床试验中使用。已经签署了旧的知情同意书且没有完成临床试验的患者，在继续进行试验之前应再次签署新的知情同意书，新、旧版本的知情同意书均须保留做原始资料。

**13.4 受试者的隐私保护**

在病例报告表、统计分析报告、临床试验报告、不良事件报告中，凡涉及到患者信息的内容均不得出现患者的真实信息。患者可通过编号和姓名缩写进行辨别。

**13.5 研究方案的修改**

本方案经伦理委员会批准后，若在实施过程中有重大修改，由临床研究负责人撰写“方案修改说明书” ，并签字，同时需报请伦理委员会批准后方可实施。未做原则性修改，由临床研究小组、统计学家、方法学家共同讨论决定并签字。

**14 、质量控制和质量保证**

**14.1 数据管理**

所有入选病例必须由指定医生完成CRF 表的填写，CRF 表中所有项目均须完成。所填写的项目不得涂改，如需修改，应先将填错的数据用一短横线划掉，然后在其上方填上正确的结果，并在旁边签名和注明修改日期。医生在填写完成后应认真检查核对所有CRF 中数据，并在CRF 表中签名，表明已检查过，务必保证数据填写的真实性、准确性。完成 的CRF 表由监察员审查后进行保管，进行数据录入与管理工作。

**14.2 本研究的质量控制**

控制内容：指标的填写是否有遗漏，是否与原始资料一致，是否准确填写。

常见问题：不同医生填写一致性不高；填写缺失、错误、与研究病例不一致。

解决方法：在本研究开始前，参与研究的医生与人员必须经过统一的培训，统一记录 方式与判断标准。确认评价指标的一致性、可靠性、完整性及准确性。在研究过程中，由PI 指派的监察员定期对研究进行监查访问及审核，以保证研究方案中的所有内容得到严格遵守、 填写CRF的正确以及填写数据的真实性、准确性。

**14.3 原始资料的保存**

本研究的原始资料包括已签署的知情同意书、有关的临床量表、实验室检验报告及功能检查报告、病例记录及其他相关记录等均应保存于成都肛肠专科医院档案室。

**14.4 试验盲法的设置**

一名研究人员将把 SPSS 软件生成的随机序列号单独放入不透明的信封中，并在信封表面按顺序编号。该研究人员将不参与其他研究，并确保装有随机序列号的信封在整个研究过程中都是密封的，这样其他人员就不会事先知道密封信封内的随机序列号。第二名研究人员将根据纳入和排除标准选择符合条件的患者纳入研究。患者将被告知研究的潜在益处和风险，如果同意参与，他们将签署知情同意书。第三名研究人员将询问患者病情、评估患者状况、协助填写 CRF 表格，并将信封分发给参与研究的患者。第四位研究人员将根据发给患者信封中的随机序列号对患者进行治疗，不参与其他研究。第五位研究人员将评估并完成每位入组患者的盆腔疼痛量表（女性）/美国国立卫生研究院慢性前列腺炎症状指数（NIH-CPSI）（男性）、DASS-21 量表和SF-36生活质量量表评估，并完成患者盆底表面肌电图和阴部神经运动诱发电位检测。该研究人员将不参与数据分析或其他研究。数据收集完成后，将进行第一次解盲，由两名研究人员分别输入和验证数据。最后，将由一名统计分析师进行统计分析，他将在不了解分组的情况下对三个组别进行比较。分析完成后，将进行第二次解盲，标志着研究结束。

**15. 数据和记录的保存**

研究者有责任确保研究记录不会受到意外损坏；受试者的医疗记录应贴上明确的标签， 以确保防止因错误意外销毁文件。研究者及研究机构须保存临床试验相关资料到临床试验 结束后五年，作为医疗文件的原始资料按照医院的有关规定保存，但不得少于临床试验结 束后五年。

**16. 研究流程**

所有受试者必须是符合EAU的 CPPS诊断标准和DSM-5的广泛性焦虑障碍或重性抑郁诊断标准。判定受试者符合纳入标准，不属于排除标准者均为潜在受试者。由受试者筛选医师对本研究受试者和家属进行告知，解释治疗的差异，征求受试者本人和家属的意见，是否同意随机分组。同意随机分组的患者签署知情同意书后，纳入本研究进行随机分组并接受疗程后随访。（见附录 1）

**17. 总结报告和论文发表**

（1）临床研究完成研究总结后可发表论文、参加国内外学术会议。

（2）本研究成果以论文形式公开发表。预计发表SCI论文1-2篇。

**附录1 研究流程图**

**
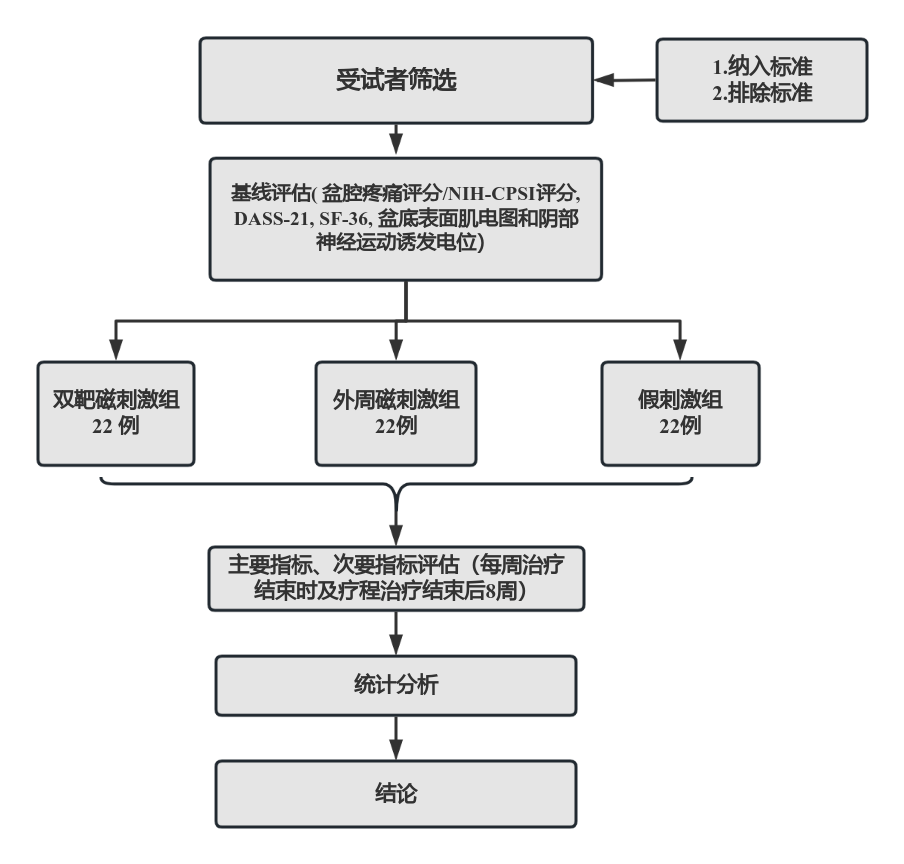
**

**附录** **2**

**表 1 评估时间表及流程**

| 阶段  项目 | 治疗前 | 第一周治疗 | 第二周治疗 | 第三周治疗 | 第四周治疗 | 疗程后8周 |
| --- | --- | --- | --- | --- | --- | --- |
|  | t0 | t1(5days) | t2(5days) | t3(5days) | t4(5days) | t5 |
| 资格筛选 | √ |  |  |  |  |  |
| 病史病历、基本资料 | √ |  |  |  |  |  |
| 常规检查 | √ |  |  |  | √ |  |
| 知情同意书 | √ |  |  |  |  |  |
| 允许的药物治疗 | √ | √ | √ | √ | √ | √ |
| 盆腔疼痛评分/NIH-CPSI评分 | √ | √ | √ | √ | √ | √ |
| DASS-21评分 | √ | √ | √ | √ | √ | √ |
| SF-36生活质量量表 | √ | √ | √ | √ | √ | √ |
| 盆底肌表面肌电图 | √ | √ | √ | √ | √ | √ |
| 阴部神经运动诱发电位 | √ | √ | √ | √ | √ | √ |
| 双靶磁刺激 |  | √ | √ | √ | √ |  |
| 外周磁刺激 |  | √ | √ | √ | √ |  |
| 假刺激 |  | √ | √ | √ | √ |  |
| 随访 |  |  |  |  |  | √ |
| 不良事件记录 |  | √ | √ | √ | √ | √ |
| 分析漏缺项目、失访者 |  |  |  |  |  | √ |

备注：常规检查包括：血常规、肝功能、肾功能和心电图检查。

**附录** **3**

**表2 盆腔疼痛评估表**

编号： 姓名：

身份证号码： 手机号码：

盆腔疼痛信息

1、请描述您的疼痛情况(如需要可另附页):

2、您认为疼痛原因是什么:

3、是否有与疼痛发病相关的事件发生? A.是 B.否

如有，相关事件是什么:

1. 您疼痛的时间有多长? 年______月

5、下列每一种症状，用评分表示过去一个月症状严重程度，请在适宜的评分所对应的方格内上打“√”

| 症状 | 评分：0-无疼痛，10-你能想象的最严重程度 | | | | | | | | | | |
| --- | --- | --- | --- | --- | --- | --- | --- | --- | --- | --- | --- |
|  | 0 | 1 | 2 | 3 | 4 | 5 | 6 | 7 | 8 | 9 | 10 |
| 你如何评价疼痛 |  |  |  |  |  |  |  |  |  |  |  |
| 排卵期疼痛(月经中期) |  |  |  |  |  |  |  |  |  |  |  |
| 经前期腹痛 |  |  |  |  |  |  |  |  |  |  |  |
| 经前疼痛(无痉挛) |  |  |  |  |  |  |  |  |  |  |  |
| 深部性交痛 |  |  |  |  |  |  |  |  |  |  |  |
| 抬腿时腹股沟痛 |  |  |  |  |  |  |  |  |  |  |  |
| 性交后疼痛持续数小时或数天 |  |  |  |  |  |  |  |  |  |  |  |
| 憋尿时疼痛 |  |  |  |  |  |  |  |  |  |  |  |
| 肌肉/关节痛 |  |  |  |  |  |  |  |  |  |  |  |
| 经期痉挛性疼痛 |  |  |  |  |  |  |  |  |  |  |  |
| 月经后疼痛消失 |  |  |  |  |  |  |  |  |  |  |  |
| 性交后阴道灼烧痛 |  |  |  |  |  |  |  |  |  |  |  |
| 尿痛 |  |  |  |  |  |  |  |  |  |  |  |
| 背痛 |  |  |  |  |  |  |  |  |  |  |  |
| 偏头痛 |  |  |  |  |  |  |  |  |  |  |  |
| 坐痛 |  |  |  |  |  |  |  |  |  |  |  |
| 合计得分 |  | | | | | | | | | | |

**附录** **4 表3 慢性前列腺炎症状指数**

编 号： 姓 名：

身份证号码： 电话号码：

| 一、疼痛或不适症状评分 | | | | | | | |
| --- | --- | --- | --- | --- | --- | --- | --- |
| 1、您在以下区域出现过疼痛或不适吗? | | 无 | 很少 | 偶尔 | 经常 | 很常见 | 几乎总是 |
|  |  | 0 | 1 | 2 | 3 | 4 | 5 |
| 1)会阴部 | |  |  |  |  |  |  |
| 2)睾丸 | |  |  |  |  |  |  |
| 3)阴茎头部 | |  |  |  |  |  |  |
| 4)腰骶部耻骨上区 | |  |  |  |  |  |  |
| 2、排尿时疼痛或烧灼感 | |  |  |  |  |  |  |
| 3、射精时或以后疼痛不适 | |  |  |  |  |  |  |
| 4、用数字描述以上疼痛或不适的感觉：不痛0 1 2 3 4 5 6 7 8 9 10很痛 | | | | | | | |
| 二、排尿症状评分 | | 无 | 少于1/5 | 少于  一半 | 大约  半数 | 多于  一半 | 几乎  每次 |
|  |  | 0 | 1 | 2 | 3 | 4 | 5 |
| 5、上周您是否经常有排尿不尽感 | |  |  |  |  |  |  |
| 6、上周您是否在两小时内排尿 | |  |  |  |  |  |  |
| 三、症状的影响 | | 无 | 有一点 | 有一些 | 很多 |  |  |
|  |  | 0 | 1 | 2 | 3 |  |  |
| 7、上述症状是否影响了您的日常生活? | |  |  |  |  |  |  |
| 8、您是否经常想起您的症状 | |  |  |  |  |  |  |
| 四、生活质量 | 非常  满意 | 满意 | 比较  满意 | 一般 | 不太  满意 | 不愉快 | 非常  恐惧 |
|  | 0 | 1 | 2 | 3 | 4 | 5 | 6 |
| 如不治疗就这样过以  后的生活，您觉得怎样? |  |  |  |  |  |  |  |
| NIH-CPSI积分结果分析 | | | | | | | |
| 疼痛和不适评分：1+2+3+4=( ) | | 症状对生活质量的影响评分：7+8+9=( ) | | | | | |
| 排尿症状评分：5+6=( ) | |  | | | | | |
| 症状严重程度：1+2+3+4+5+6=( ) | | 轻度0~9 | | 中度10~18 | | 重度19~31 | |
| 总体评分：1+2+3+4+5+6+7+8+9=( ) | | 轻度1~14 | | 中度15~29 | | 重度30~43 | |

**附录** **5**

**表4 抑郁焦虑和压力量表（DASS-21）**

编 号： 姓 名：

身份证号码： 电话号码：

指导语：仔细阅读下面每个句子，“过去一周”您是否出现过这些情况，请选择适合您的选项。

将各分量表得分乘以2，即为该分量表的分值，分值越高代表越具有这种情绪。

|  |  | 不符合 | 有时符合 | 常常符合 | 总是符合 | 项目评分 |
| --- | --- | --- | --- | --- | --- | --- |
| 1 | 我好像不再有任何愉快、舒畅的感觉 |  |  |  |  |  |
| 2 | 我感到很难主动去开始工作 |  |  |  |  |  |
| 3 | 我觉得自己对将来没有什么可盼望 |  |  |  |  |  |
| 4 | 我感到忧郁沮丧 |  |  |  |  |  |
| 5 | 我对任何事也不热衷 |  |  |  |  |  |
| 6 | 我觉得自己不怎么配做人 |  |  |  |  |  |
| 7 | 我感到生命毫无意义 |  |  |  |  |  |
| 抑郁分量表：10、14 及 21 分别是轻微、中度及重度抑郁的临界值 | | | | | |  |
| 8 | 我感到口干舌燥 |  |  |  |  |  |
| 9 | 我感到呼吸困难（例如：不做运动时也感到气促或透不过气来） |  |  |  |  |  |
| 10 | 我感到颤抖（例如手震） |  |  |  |  |  |
| 11 | 我担心一些令自己恐慌或出丑的场合 |  |  |  |  |  |
| 12 | 我感到快要恐慌了 |  |  |  |  |  |
| 13 | 我察觉自己在没有明显的体力劳动时，也感到心律不正常 |  |  |  |  |  |
| 14 | 我无缘无故地感到害怕 |  |  |  |  |  |
| 焦虑分量表：8、10 及 15 分别为轻微、中度及重度焦虑的临界值 | | | | | |  |
| 15 | 我觉得很难让自己安静下来 |  |  |  |  |  |
| 16 | 我对事情往往做出过敏反应 |  |  |  |  |  |
| 17 | 我觉得自己消耗很多精神 |  |  |  |  |  |
| 18 | 我感到忐忑不安 |  |  |  |  |  |
| 19 | 我感到很难放松自己 |  |  |  |  |  |
| 20 | 我无法容忍任何阻碍我继续工作的事情 |  |  |  |  |  |
| 21 | 我发觉自己很容易被触怒 |  |  |  |  |  |
| 压力分量表：15、19 及 26 分别为轻微、中度及重度压力的临界值 | | | | | |  |

**附录 6**

**表5 SF-36生活质量量表**

编号： 姓名：

身份证号码： 手机号码：

以下共 36 个问题，每个问题后都有几个答案供选择，请将您认为合适的答案对应 分值填入表格条目对应的得分栏。

| 条目 |  | | | | | | |  | | | | |  | | | | | | |  | | | | |  | | 得分 |  |
| --- | --- | --- | --- | --- | --- | --- | --- | --- | --- | --- | --- | --- | --- | --- | --- | --- | --- | --- | --- | --- | --- | --- | --- | --- | --- | --- | --- | --- |
| 1、总体来讲，您的健康 状况是 | ①非常好 | | | | | | | ②很好 | | | | | ③好 | | | | | | | ④一般 | | | | | ⑤差 | |  |  |
|  | 5 | | | | | | | 4.4 | | | | | 3.4 | | | | | | | 2 | | | | | 1 | |  |  |
| 2、跟 1 年以前比您觉得 自己的健康状况是 | ①好多了 | | | | | | | ②好一些 | | | | | ③差不多 | | | | | | | ④差一些 | | | | | ⑤差多了 | |  |  |
|  | 1 | | | | | | | 2 | | | | | 3 | | | | | | | 4 | | | | | 5 | |  |  |
| **健康和日常活动** | | | | | | | | | | | | | | | | | | | | | | | | | | |  |  |
| 3、以下这些问题都和日常活动有关。请您想一想， 您的健康状况是否限制了这些活动？ 如果有限 制，程度如何？ | | | | | | | | | | | | | ① 限 制 很大 | | | | | | ② 有 些 限 制 | | | | | ③ 毫 无 限 制 | | |  |  |
| （1）重体力活动。如跑步举重、参加剧烈运动等 | | | | | | | | | | | | | 1 | | | | | | 2 | | | | | 3 | | |  |  |
| (2)适度的活动。如移动一张桌子、扫地、打太极 拳、做简单体操等 | | | | | | | | | | | | | 1 | | | | | | 2 | | | | | 3 | | |  |  |
| （3）手提日用品。如买菜、购物等 | | | | | | | | | | | | | 1 | | | | | | 2 | | | | | 3 | | |  |  |
| （4）上几层楼梯 | | | | | | | | | | | | | 1 | | | | | | 2 | | | | | 3 | | |  |  |
| （5）上一层楼梯 | | | | | | | | | | | | | 1 | | | | | | 2 | | | | | 3 | | |  |  |
| （6）弯腰、屈膝、下蹲 | | | | | | | | | | | | | 1 | | | | | | 2 | | | | | 3 | | |  |  |
| （7）步行 1500 米以上的路程 | | | | | | | | | | | | | 1 | | | | | | 2 | | | | | 3 | | |  |  |
| （8）步行 1000 米的路程 | | | | | | | | | | | | | 1 | | | | | | 2 | | | | | 3 | | |  |  |
| （9）步行 100 米的路程 | | | | | | | | | | | | | 1 | | | | | | 2 | | | | | 3 | | |  |  |
| （10） 自己洗澡、穿衣 | | | | | | | | | | | | | 1 | | | | | | 2 | | | | | 3 | | |  |  |
| 4、在过去 4 个星期里，您的工作和日常活动有无因为身体健康的 原因而出现以下这些问题？ | | | | | | | | | | | | | | | | | | | | | 不是 | | | | | 是 |  |  |
| （1）减少了工作或其他活动时间 | | | | | | | | | | | | | | | | | | | | | 1 | | | | | 2 |  |  |
| （2）本来想要做的事情只能完成一部分 | | | | | | | | | | | | | | | | | | | | | 1 | | | | | 2 |  |  |
| （3）想要干的工作或活动种类受到限制 | | | | | | | | | | | | | | | | | | | | | 1 | | | | | 2 |  |  |
| （4）完成工作或其他活动困难增多（比如需要额外的努力） | | | | | | | | | | | | | | | | | | | | | 1 | | | | | 2 |  |  |
| 5、在过去 4 个星期里，您的工作和日常活动有无因为情绪的原因 （如压抑或忧虑）而出现以下这些问题？ | | | | | | | | | | | | | | | | | | | | | 不是 | | | | | 是 |  |  |
| （1）减少了工作或活动时间 | | | | | | | | | | | | | | | | | | | | | 1 | | | | | 2 |  |  |
| （2）本来想要做的事情只能完成一部分 | | | | | | | | | | | | | | | | | | | | | 1 | | | | | 2 |  |  |
| （3）干事情不如平时仔细 | | | | | | | | | | | | | | | | | | | | | 1 | | | | | 2 |  |  |
| 6、在过去 4 个星期里，您的健康或 情绪不好在多大程度上影响了您与 家人、朋友、邻居或集体的正常社 会交往？ | | | | | | 完全没 有影响 | | | | | | 有一点 影响 | | | | | 中等 影响 | | | | 影响很 大 | | | | | 影响非常 大 |  |  |
|  |  |  |  |  |  | 5 | | | | | | 4 | | | | | 3 | | | | 2 | | | | | 1 |  |  |
| 7、在过去 4 个星期里， 您有身体疼痛吗？ | 完全没 有疼痛 | | | | 稍微有 一点 | | | | | 有一点 疼痛 | | | | | | 中等疼 痛 | | | | | | 严重疼 痛 | | | | 很严重 疼痛 |  |  |
|  |  | | | |  | | | | |  | | | | | |  | | | | | |  | | | |  |  |  |
|  | 6 | | | | 5.4 | | | | | 4.2 | | | | | | 3.1 | | | | | | 2.2 | | | | 1 |  |  |
| 8、在过去 4 个星期里， 您的身体疼痛影响了您 的工作和家务吗？ | 完全没 有影响 | | | | 有一点 影响 | | | | | 中等影 响 | | | | | | 影响很 大 | | | | | | 影响非 常大 | | | | 备注 |  |  |
|  | 6 | | | | 4.75 | | | | | 3.5 | | | | | | 2.25 | | | | | | 1 | | | | 7 无 8 无 |  |  |
|  | 5 | | | | 4 | | | | | 3 | | | | | | 2 | | | | | | 1 | | | | 7 有 8 无 |  |  |
| **您的感觉** | | | | | | | | | | | | | | | | | | | | | | | | | | | | |
| 9、以下这些问题是关于过去 1 个月里您自己的感觉，对 每一条问题所说的事情，您 的情况是什么样的？ | | ①所有 的时间 | | ②大部 分时间 | | | | | ③比较 多时间 | | | | | ④ 一部 分时间 | | | | ⑤小部 分时间 | | | | | ⑥没有这种感 觉 | | | |  | |
| （1）您觉得生活充实 | | 6 | | 5 | | | | | 4 | | | | | 3 | | | | 2 | | | | | 1 | | | |  | |
| （2）您是一个敏感的人 | | 1 | | 2 | | | | | 3 | | | | | 4 | | | | 5 | | | | | 6 | | | |  | |
| （3）您的情绪非常不好，什 么事都不能使您高兴起来 | | 1 | | 2 | | | | | 3 | | | | | 4 | | | | 5 | | | | | 6 | | | |  | |
| （4）您的心理很平静 | | 6 | | 5 | | | | | 4 | | | | | 3 | | | | 2 | | | | | 1 | | | |  | |
| （5）您做事精力充沛 | | 6 | | 5 | | | | | 4 | | | | | 3 | | | | 2 | | | | | 1 | | | |  | |
| （6）您的情绪低落 | | 1 | | 2 | | | | | 3 | | | | | 4 | | | | 5 | | | | | 6 | | | |  | |
| （7）您觉得筋疲力尽 | | 1 | | 2 | | | | | 3 | | | | | 4 | | | | 5 | | | | | 6 | | | |  | |
| （8）您是个快乐的人 | | 6 | | 5 | | | | | 4 | | | | | 3 | | | | 2 | | | | | 1 | | | |  | |
| （9）您感觉厌烦 | | 1 | | 2 | | | | | 3 | | | | | 4 | | | | 5 | | | | | 6 | | | |  | |
| 10、不健康影响了您的社会 活动（如走亲访友） | | 1 | | 2 | | | | | 3 | | | | | 4 | | | | 5 | | | | | 6 | | | |  | |
| **总体健康情况** | | | | | | | | | | | | | | | | | | | | | | | | | | | | |
| 11、请看下列每一条问题，哪一 种答案最符合您的情况？ | | | ①绝对 正确 | | | | ② 大 部 分正确 | | | | ③ 不 能 肯定 | | | | ④大部 分错误 | | | | | | | ⑤ 绝对错 误 | | | | |  | |
| （1）我好象比别人容易生病 | | | 1 | | | | 2 | | | | 3 | | | | 4 | | | | | | | 5 | | | | |  | |
| （2）我跟周围人一样健康 | | | 5 | | | | 4 | | | | 3 | | | | 2 | | | | | | | 1 | | | | |  | |
| （3）我认为我的健康状况在变坏 | | | 1 | | | | 2 | | | | 3 | | | | 4 | | | | | | | 5 | | | | |  | |
| （4）我的健康状况非常好 | | | 5 | | | | 4 | | | | 3 | | | | 2 | | | | | | | 1 | | | | |  | |
| 换算得分=（实得分-该方面最低分）/（该方面最高分-该方面最低分） ×100； 缺失条目的得分用其所属方面的平均分代替 | | | | | | | | | | | | | | | | | | | | | | | | | | | 换算  得分 |  |
| 生理机能（PF：Physical Functioning）条目 3；PF=（得分-10）/20×100 | | | | | | | | | | | | | | | | | | | | | | | | | | |  |  |
| 生理职能（RP：Role-Physical）条目 4；RP=（得分-4）/4×100 | | | | | | | | | | | | | | | | | | | | | | | | | | |  |  |
| 躯体疼痛（BP：Bodily Pain），条目 7,8；BP=（得分-2）/10×100 | | | | | | | | | | | | | | | | | | | | | | | | | | |  |  |
| 一般健康状况（GH：General Health），条目 1,10；GH=（得分-5）/20×100 | | | | | | | | | | | | | | | | | | | | | | | | | | |  |  |
| 精力（VT：Vitality），条目 9（1,5,7,9）；VT=（得分-4）/20×100 | | | | | | | | | | | | | | | | | | | | | | | | | | |  |  |
| 社会功能（SF：Social Functioning）条目 6,9（10）；SF=（得分-2）/9×100 | | | | | | | | | | | | | | | | | | | | | | | | | | |  |  |
| 情感职能（RE：Role-Emotional）条目 5；RE=（得分-3）/3×100 | | | | | | | | | | | | | | | | | | | | | | | | | | |  |  |
| 精神健康（MH：Mental Health）条目 9（2,3,4,6,8,）；MH=（得分-5）/25×100 | | | | | | | | | | | | | | | | | | | | | | | | | | |  |  |
| 健康变化（HT：Reported Health Transition）条目 2；HT=（得分-1）/4×100 | | | | | | | | | | | | | | | | | | | | | | | | | | |  |  |

**附录7 随机化申请表**

申请日期: 年 月 日 随机号：

| **一般资料** | | | |
| --- | --- | --- | --- |
| 姓名缩写： | | 年龄：岁 | 性别：男□ 女□ |
| 体重： Kg | | 身高： m | BMI指数： Kg/m2 |
| DMS-5诊断 | 广泛性焦虑障碍 程度：轻度□、 中度□、 重度□ | | |
|  | 重性抑郁程度：轻度□、 中度□、 重度□ | | |

| **入选标准核对表** | | |
| --- | --- | --- |
| **入选标准** | **是** | **否** |
| 1.符合2022年EAU指南中的CPPS诊断标准 | □ | □ |
| 2. 明确诊断为广泛性焦虑障碍或重度抑郁症 | □ | □ |
| 3. 年龄在18岁至70岁之间 | □ | □ |
| 4.体格检查和辅助检查未见明显病理变化 | □ | □ |
| 5.在就诊前3个月内，除口服药物外没有其他治疗 | □ | □ |
| 6.患者知情同意并自愿参与研究 | □ | □ |
| **以上各条标准必须全部为“是” ，否则患者无法入组。** | | |

| **排除标准核对表** | | |
| --- | --- | --- |
| **排除标准** | **是** | **否** |
| 1. 急性全身和颅内出血性疾病患者 | □ | □ |
| 2. 患有严重基础疾病的个体，如心血管、肝脏、肾脏、呼吸系统和血液疾病，以及恶性肿瘤和其他进展性疾病 | □ | □ |
| 3.患有心脏金属膜、心脏起搏器、颅内金属植入物、腰椎金属植入物和可植入电子设备的患者 | □ | □ |
| 4.在头部或腰骶区域有感染的个体 | □ | □ |
| 5.表现出生命体征不稳定的个体 | □ | □ |
| 6.对磁疗有过不良反应的患者 | □ | □ |
| 7.具有非典型自主神经反射的个体 | □ | □ |
| 8. 无法配合的认知障碍患者 | □ | □ |
| 9. 孕妇或哺乳期妇女 | □ | □ |
| 10. 有患有导致外周神经损伤的疾病史的患者 | □ | □ |
| 11. 患有严重疾病的患者，如恶性渗出、活动性肺结核、癌症或重症肌无力 | □ | □ |
| 12. 患有严重精神疾病或癫痫的患者 | □ | □ |
| **以上各条标准必须全部为“否” ，否则患者无法入组。** | | |

**是否符合入组：**是□ 否□ 未入组原因

**符合入组者随机化结果**：A 组(双靶磁刺激)□ B 组（外周磁刺激）□ C组（假刺激）□

**感谢您的参与！**
