# Supplementary material for: Dual-target peripheral and central magnetic stimulation for rehabilitation of chronic pelvic pain syndrome associated with psychosomatic symptoms: Study protocol for a randomized controlled trial
Source: PLoS One. 2025 Jul 17;20(7):e0326740. doi: 10.1371/journal.pone.0326740 (PMC12270166; doi:10.1371/journal.pone.0326740)
Supplement: S4 File — (DOCX) [file pone.0326740.s004.docx]

**Chengdu Anorectal Hospital Clinical Research**

**Application Form**

**Declaration Type:** [ ] Large Cohort Study [ ] Key Project [ √] General Project

**Project Title:** Peripheral and Central Dual-target Magnetic Stimulation to Promote Rehabilitation of Chronic Pelvic Pain Syndrome with Psychosocial Disorders: Randomized Controlled Trial Protocol

**Applicant:** Chunmei Luo

**Department:** Gastroenterology (Constipation)

**Contact number:** 18708102327

**Email：** luochunmei@stu.cdutcm.edu.cn

**Version number：** 2.0

Version date： 2023.10.20

Chengdu Anorectal Hospital Clinical Research Center

一、Department Opinion

| Applicant's Commitment  I guarantee the authenticity of the information provided in this application. If funding is obtained, I and the members of this project team will strictly adhere to the relevant regulations of the "Clinical Research Management Measures (Trial)" of Chengdu Anorectal Specialty Hospital, ensure that research work is conducted during the designated time, diligently carry out the research work as planned, and submit the relevant materials on time.  Applicant (Seal): |
| --- |
| Department Opinion： Agree  Department Head (Seal)： |

**二.** Basic Information

| Applicant Information | Name | | Chunmei Luo | Gender | Female | Date of Birth | | February 1986 | Ethnicity | | Han Chinese |
| --- | --- | --- | --- | --- | --- | --- | --- | --- | --- | --- | --- |
|  | Title | | Associate Chief Physician | | | Department | | Department of Constipation | | | |
|  | Degree | | Master's degree | | | Year of Award | | 2014 | | | |
|  | Tel. | | 18708102327 | | | Email | | luochunmei@stu.tcm.edu.cn | | | |
|  | Main  Research Areas | | The Foundation and Clinical Research of Pelvic Floor Dysfunction Diseases | | | | | | | | |
| Project Basic Information | Project Title | | Peripheral and Central Dual-target Magnetic Stimulation to Promote Rehabilitation of Chronic Pelvic Pain Syndrome with Psychological Disorders: Randomized Controlled Trial Protocol | | | | | | | | |
|  | Research Type | | [ √] Clinical Trial (Randomized Controlled Trial) [ ] Case-Control Study  [ ] Cohort study [ ] Cross-sectional study [ ] Diagnostic study [ ] Other  (Please mark "√" in front of the research type that best fits.) | | | | | | | | |
|  | Research duration | | January 2024 to December 2025 | | | | Application for funding | | | 50,000 yuan | |
| Abstract | Chronic pelvic pain syndrome (CPPS) has a high incidence and can present with severe symptoms, leading to mental health issues, causing anxiety, depression, and pain catastrophizing, and placing significant stress on families and society. The exact causes and mechanisms of CPPS remain unclear; studies suggest it may be caused by psychological issues and multi-system dysfunction. Therefore, addressing psychological problems is crucial in the treatment of CPPS. Repetitive peripheral magnetic stimulation (rPMS) shows potential effectiveness in treating CPPS, while repetitive transcranial magnetic stimulation (rTMS) has been proven to be effective for anxiety and depression. This study employs a randomized, double-blind method, recruiting 66 CPPS participants with accompanying psychological issues, who are stratified by gender and randomly assigned to three groups (1:1:1): double-target magnetic stimulation group, peripheral magnetic stimulation group, and sham stimulation group, receiving the corresponding treatments. The efficacy and safety of double-target magnetic stimulation in CPPS patients with psychological disorders will be assessed by comparing changes in pelvic pain scores (for females) or the National Institutes of Health Chronic Prostatitis Symptom Index (NIH-CPSI, for males), depression, anxiety, and stress scales (DASS-21), pelvic floor surface electromyography, pudendal nerve motor evoked potentials, and the 36-item Short Form Health Survey (SF-36) scores before and after treatment. | | | | | | | | | | |
| Keywords | | Repetitive Transcranial Magnetic Stimulation, Repetitive Peripheral Magnetic Stimulation, Chronic Pelvic Pain Syndrome, Randomized Controlled Trial | | | | | | | | | |

**三.**Project Team Members (including Principal Investigators)

| Name | Unit | Dgree | Title | Task division | Ethics training | GCP training | Signature |
| --- | --- | --- | --- | --- | --- | --- | --- |
| ChunmeiLuo | Chengdu Anorectal Hospital | Master's degree | Associate Chief Physician | Project Leader | Yes | Yes |  |
| Xiangdong Yang | Chengdu Anorectal Hospital | Doctor | Professor | Project Guidance | Yes | Yes |  |
| Duigui Chang | Chengdu University of Traditional Chinese Medicine Affiliated Hospital | Doctor | Chief Physician | Supervision of Project Implementation | Yes | Yes |  |
| Jiabei He | Sichuan Province Fifth People's Hospital | Master's degree | Attending physician | Data organization, statistical analysis | Yes | Yes |  |
| Haibo Lan | Chengdu Anorectal Hospital | Master's degree | Associate Chief Physician | Project implementation, Observation and follow-up of cases. | No | Yes |  |
| Meizhu Zhao | Chengdu Anorectal Hospital | Master's degree | Associate Chief Physician | Project data collection and analysis | No | No |  |
| Xiaobin Zhen | Chengdu Anorectal Hospital | Undergraduate | Attending physician | Case follow-up, recording | No | No |  |
| Ren Liu | Chengdu Anorectal Hospital | Master's degree | Attending physician | Case follow-up, recording | No | No |  |
| Lanjin Bai | Chengdu Anorectal Hospital | Master's degree | Resident physician | Project Implementation | No | No |  |
| Xueqian Li | Chengdu Anorectal Hospital | Undergraduate | Resident physician | Project Implementation | No | No |  |
| Siyi Tian | Chengdu Anorectal Hospital | Undergraduate | Head Nurse | Observation and follow-up of cases. | No | No |  |

Project Secretary: Xueqian Li Contact Number: 19980717169 Email: 469243152@qq.com

四. Applicant's Work Accumulation

| The applicant has accumulated rich experience in clinical work, and the relevant technical reserves required for the implementation of this project are sufficient.   1. I have been engaged in the field of proctology since 2011, with over 10 years of clinical experience. I currently serve as the Deputy Secretary-General of the Chinese Society of Constipation Medicine, the Secretary of the Pelvic Floor Disease Prevention Branch of the Sichuan Provincial Preventive Medicine Association, a member of the Colorectal Committee of the Sichuan Provincial Women Physicians Association, and a council member of the Chinese Graduate Union of Colorectal Disease Research, among other positions in various professional societies.   2. The relevant technical reserves required for this project are sufficient: After graduating with a master's degree in 2014, I determined to focus on clinical research related to pelvic floor dysfunction as my professional research direction. I have been engaged in research on chronic refractory constipation for a long time and am skilled in diagnosing and treating difficult diseases such as chronic refractory constipation, persistent anal heaviness, anal rectal pain, chronic prostatitis, fecal incontinence, and overactive bladder. I have also undertaken municipal-level research projects such as "Research on the Treatment Patterns of Persistent Anal Heaviness and Pain" and hospital-level research projects like "Research on the Treatment Patterns of Gastrointestinal and Anorectal Diseases Combined with Mental and Psychological Disorders." |
| --- |

五. Analysis of Innovation and Feasibility

| 1. Features and Innovations of this Project  This study employs a prospective randomized controlled trial design, setting up a control group experiment. The control group will consist of CPPS patients receiving sham stimulation and those receiving peripheral magnetic stimulation, while the experimental group will consist of CPPS patients receiving dual-target magnetic stimulation. By comparing the clinical safety and efficacy indicators among the three groups of patients, the study aims to explore the therapeutic effects and safety of dual-target magnetic stimulation in CPPS patients with comorbid psychological disorders. Dual-target magnetic stimulation is derived from the author's team's years of clinical experience and in-depth understanding of various CPPS treatment methods. Currently, no relevant studies have been found in domestic and international literature regarding the use of this type of treatment for CPPS patients with comorbid psychological issues.   1. Feasibility Analysis (Department Conditions, Personnel Allocation, Case Sources, Technical Conditions, etc.)   Chengdu Anorectal Specialty Hospital is a tertiary first-class specialty hospital that integrates medical treatment, teaching, research, and preventive healthcare into a medical center. Its anorectal department is a nationally key clinical specialty, and the constipation department and pelvic floor center have a significant influence in the western region of China. The treatment for this clinical research will be conducted at the hospital's pelvic floor center.  Our team has accumulated certain clinical experience in the application effects and safety of peripheral magnetic stimulation in the treatment of CPPS. We have established standardized procedures for using transcranial magnetic stimulation to treat mental disorders such as anxiety and depression. According to relevant reports, the incidence of CPPS has shown an upward trend both domestically and internationally, and the presence of psychological issues is a major reason for its poor treatment outcomes. Therefore, it is particularly important to find effective treatment methods for CPPS patients with coexisting psychological disorders.  The research team currently has 5 senior technical staff members, 8 individuals with a master's degree or higher, and possesses rich experience in clinical research, patient follow-up, and statistical analysis. This ensures the accuracy of clinical data recording and statistical analysis. The talent structure is reasonable, with clear division of labor, which guarantees the smooth completion of this project. |
| --- |

1. Progress schedule and expected goals

| 1. Progress Plan (including annual task objectives, assessment indicators, and the planned progress for completing research and development content. It should be filled out annually, with each year's research plan and objectives being clear, measurable, and corresponding to the overall research tasks, completion timeline, and assessment indicators of the project.)  2024.01-2025.01 ① Successfully enrolled 40 cases of CPPS patients (conducting randomization and providing corresponding treatments), collected relevant clinical indicators and postoperative complication occurrences, and systematically recorded the data.  2025.02-2025.07 ② cases of CPPS patients were successfully enrolled (randomized grouping was conducted, and corresponding treatments were administered). Relevant clinical indicators and postoperative complications of the patients were collected, and the data was systematically recorded.  2025.08-2025.10 ③ Conduct statistical analysis on the collected data.  2025.11-2025.12 ④ Write a thesis.  2. Expected Goals and Assessment Indicators (Focus on describing the final outcomes and forms of this research, such as papers, results, patents, etc.; the content of the assessment indicators must be complete and clear, capable of evaluating the degree of completion of the project and its actual effects.)  (1) Investigate the application effects and safety of dual-target magnetic stimulation in the treatment of CPPS patients.  (2) Publish 2 SCI articles related to clinical research. |
| --- |

**七．Budget allocation**

| Serial Number | Budget Item Name | Budget (in ten thousand) | Remarks (Basis and Explanation of Calculations) |
| --- | --- | --- | --- |
| 1 | Clinical research business expenses | 3 | The costs for the preparation of the project proposal and CRF form, training fees for research assistants and researchers, labor costs for graduate students, expenses for external study, literature retrieval, and other related costs. |
| 2 | Consultation fees, research fees, and labor fees | 1 | Expert consultation fees, publication of papers, editing, follow-up fees for enrolled case investigations, etc. |
| 3 | Data fee | 0.3 | Copying and printing fees, data statistical analysis fees, etc. |
| 4 | Collaboration fee | 0.5 | The expenses incurred for collaboration or research during the implementation of the project. |
| 5 | Transportation expenses | 0.2 | Transportation expenses for studying abroad. |
|  | Total | 5 |  |

**Peripheral combined central dual-target magnetic stimulation for rehabilitation of chronic pelvic pain syndrome with psychosomatic disorders: Study protocol for a randomized controlled trial**

**Research Plan**

Research Leader: Luo Chunmei

Applicant: Chengdu Anorectal Specialty Hospital

Version number: 2.0

Version date: 2023-10-20

**Table of Contents**

[Plan Summary](#_Toc18536) 3

[1. Basis for the Topic 6](#_Toc21208)

[References 7](#_Toc6323)

[2. Research Purpose 9](#_Toc10318)

[3. Research subject 1](#_Toc25580)0

[3.1 Selection Criteria 1](#_Toc2035)0

[3.2 Exclusion Criteria 10](#_Toc5291)

[3.3 Exit Standard 11](#_Toc19402)

[4. Plan Design 11](#_Toc30603)

[5. Sample Size Estimation and Grouping 1](#_Toc1563)1

[5.1 Sample Size Estimation 11](#_Toc21861)

[5.2 Patient ID and randomization group 12](#_Toc12384)

[6. Research steps or treatment plan 12](#_Toc17164)

[6.1 Clinical intervention measures and steps 12](#_Toc17021)

[6.2 Bilateral Magnetic Stimulation Treatment Protocol 13](#_Toc25465)

[6.3 Peripheral magnetic stimulation treatment protocol 13](#_Toc4490)

[6.4 Placebo treatment protocol 13](#_Toc18302)

[6.5 Treatment plan 13](#_Toc19356)

[7. Follow-up plan 14](#_Toc23040)

[8. Evaluation indicators of research results 14](#_Toc30858)

[8.1 Main therapeutic efficacy indicators 14](#_Toc12794)

[8.2 Secondary efficacy indicators 14](#_Toc12279)

[8.3 Safety Indicators 15](#_Toc15048)

[9. Safety Assessment 15](#_Toc16620)

[9.1 Benefit 15](#_Toc23531)

[9.2 Risk 1](#_Toc24344)6

[9.3 Risk prevention measures 16](#_Toc961)

[10. Recruitment Process 2](#_Toc8910)4

[10.1 Recruitment Process 24](#_Toc8745)

[10.2 Startup time 25](#_Toc30320)

[10.3 Recruitment Materials 26](#_Toc16219)

[10.4 Expected number of recruits 26](#_Toc355)

[10.5 The time the research subjects participated in the study. 26](#_Toc28005)

[11 . Data Management and Statistical Analysis Plan 26](#_Toc14785)

[11.1 Data processing 26](#_Toc28533)

[11.2 Statistical analysis 27](#_Toc23087)

[12 .Confidentiality Plan 28](#_Toc8692)

[13. Ethical requirements and informed consent 28](#_Toc28794)

[13.1 Obtain ethical committee approval 28](#_Toc6746)

[13.2 Informed consent 28](#_Toc1943)

[13.3 Revisions to the Informed Consent Form 29](#_Toc15801)

[13.4 Protection of Participants' Privacy 29](#_Toc9845)

[13.5 Modification of the research plan 29](#_Toc31942)

[14 . Quality Control and Quality Assurance 29](#_Toc29300)

[14.1 Data Management 29](#_Toc26869)

[14.2 Quality Control of This Study 3](#_Toc31362)0

[14.3 Preservation of Original Data 3](#_Toc13216)0

[14.4 Setting up a blind test 3](#_Toc6183)0

[15. Data and Record Preservation 3](#_Toc28507)1

[16. Research Process 3](#_Toc32276)1

[17. Summary report and paper publication 3](#_Toc27867)1

[Appendix 1 3](#_Toc6167)2

Appendix 2 [3](#_Toc15674)3

[Appendix 3 3](#_Toc8090)4

[Appendix 4 3](#_Toc19027)5

[Appendix 5 3](#_Toc6167)6

Appendix 6 [3](#_Toc15674)8

[Appendix 7 4](#_Toc8090)1

Plan Summary

| Research Title | Peripheral combined central dual-target magnetic stimulation for rehabilitation of chronic pelvic pain syndrome with psychosomatic disorders: Study protocol for a randomized controlled trial |
| --- | --- |
| Principal Investigator | Deputy Chief Physician Chunmei Luo |
| Research Purpose | This study aims to evaluate the effectiveness and safety of repeated peripheral magnetic stimulation (rPMS) combined with repeated transcranial magnetic stimulation (rTMS) in treating patients with pelvic pain syndrome accompanied by psychological disorders. It will assess the impact of dual-target magnetic stimulation on patients' pelvic pain scale/NIH-CPSI scores, depression, anxiety, and stress (DASS-21) scores, as well as the SF-36 quality of life scale, and the effects on pelvic floor muscle surface electromyography and pudendal nerve motor evoked potentials. Additionally, it will compare the effects of dual-target magnetic stimulation and peripheral magnetic stimulation on pain levels, psychological status, and quality of life in patients with chronic pelvic pain syndrome accompanied by psychological disorders, providing a basis for clinical treatment options. |
| Main evaluation indicators (efficacy indicators) | Pelvic Pain Scale (Female) / NIH-CPSI (Male) |
| Secondary evaluation indicators (efficacy indicators) | Surface electromyography of the pelvic floor muscles  Pudendal nerve motor evoked potentials  Depression Anxiety and Stress Scale (DASS-21)  SF-36 Quality of Life Scale |
| Safety indicators | Complete Blood Count (CBC)  Liver and kidney function  Electrocardiogram |
| Selection Criteria | (1) Meets the diagnostic criteria for Chronic Pelvic Pain Syndrome (CPPS) according to the European Association of Urology (EAU) guidelines for chronic pelvic pain (2022 edition).  (2) Age: 18-70 years old.  (3) Generalized Anxiety Disorder or Major Depressive Disorder that meets DSM-5 criteria.  (4) No significant pathological changes were observed in the physical examination and auxiliary examinations.  (5) No other treatments except for oral medications in the three months prior to the visit.  (6) The patient is informed and voluntarily participates in the study. |

| Exclusion Criteria | (1) Patients with acute systemic and intracranial hemorrhagic diseases.  Individuals with severe underlying health conditions, such as cardiovascular, liver, kidney, respiratory, and blood diseases, as well as malignant tumors and other progressive diseases.  (3) Patients with cardiac metal membranes, cardiac pacemakers, intracranial metal implants, lumbar metal implants, and implantable electronic devices.  (4) Individuals with infections in the head or sacral region.  (5) Individuals exhibiting unstable vital signs.  (6) Patients who have had adverse reactions to magnetic therapy.  (7) Individuals with atypical autonomic reflexes.  (8) Patients with cognitive impairments who cannot cooperate.  (9) Pregnant or breastfeeding women.  (10)Patients with a history of diseases that lead to peripheral nerve damage.  (11)Patients with serious illnesses, such as malignant effusion, active tuberculosis, cancer, or myasthenia gravis.  (12)Patients with severe mental illness or epilepsy. |
| --- | --- |
| Exit Standard | Participants will be asked to withdraw from the RCT under the following circumstances:  (1) If the patient does not adhere to the prescribed treatment plan or receives alternative treatment;  (2) If the patient experiences severe adverse reactions or changes in their condition that prevent them from continuing to participate in the trial;  (3) If the patient experiences significant organ dysfunction, unstable blood pressure, abnormal heart rate, or difficulty breathing;  (4) If the patient requests to withdraw informed consent due to intolerable adverse reactions or for no specific reason;  (5) If the patient loses follow-up during treatment or follow-up. |
| Sample size verification of subjects | According to the research by Yang Jing-ming and his colleagues, under the conditions of β=0.1 and α=0.05, the effect size is 0.5. Pain scores will be used as the primary measurement standard, and G*Power 3.1.9.7 will be used to estimate the sample size. After calculations, it is determined that there will be approximately 54 patients in the study cohort. Considering a 10% dropout rate, each group will include 22 people, and this RCT will require 66 participants. |
| Treatment plan | (1) Double-target magnetic stimulation group treatment procedure: ① Before treatment: Three checks and seven pairs, conducted by a qualified practitioner for risk assessment, including the patient's mental symptoms, medication treatment effects, and contraindications, etc. ② During treatment: Determine the stimulation plan: individualized stimulation intensity, measure the resting motor threshold during the first treatment.  Rest motor threshold (RMT) determines the magnetic stimulation intensity for the patient, with the rTMS stimulation intensity set at 120% of RMT. Auxiliary positioning devices: based on the positioning cap, the location of the stimulation cortex is determined; for depressed patients, the left DLPFC is selected for 10Hz high-frequency stimulation, while for anxious patients, the right DLPFC is selected for 1Hz low-frequency stimulation. The chair is adjusted to a horizontal position of 10-20°, and the patient lies prone on the chair, with the center of the circular coil aligned with the S3 region. During stimulation, a contraction sensation in the anal sphincter muscles and a plantar flexion response indicate correct positioning. rPMS is performed first, followed by adjusting the chair to a sitting position for rTMS. After the treatment is completed, the patient is asked about any discomfort, and elderly patients are advised to sit by the bedside for a moment before leaving. The doctor completes all types of record forms and keeps them for future reference; the treatment room is disinfected and isolated.  (2) Peripheral magnetic stimulation group: ① Before treatment: same as the double-target magnetic stimulation group. ② During treatment: only rPMS is performed, with the same operation as the double-target magnetic stimulation group. ③ After treatment: same as the double-target magnetic stimulation group.  (3) Sham stimulation group: ① Before treatment: Same as the dual-target magnetic stimulation group. ② During treatment: Use coils that do not generate a magnetic field, only producing sound, first applying sham peripheral magnetic stimulation to the S3 region, followed by sham transcranial magnetic stimulation, with the same procedure as the dual-target magnetic stimulation group. ③ After treatment: Same as the dual-target magnetic stimulation group.  (4) Treatment regimen: A course consists of 20 sessions, with 5 sessions per week, and 1 session each day; patients need to actively inform the therapist when there are significant adjustments to the types and dosages of medications.  (5) Emergency Situation Handling: The system should provide protective measures in accordance with the requirements of GB9706.1-2007, such as an emergency braking device. During the treatment process, if unexpected seizures, loss of consciousness, or other emergencies occur, immediately take the following measures for handling and first aid: ① Immediately stop rTMS treatment; ② Ensure the patient's airway is clear; ③ Closely monitor the patient's vital signs; ④ For cardiac arrest or respiratory failure, immediately perform cardiopulmonary resuscitation; ⑤ Contact the patient's family to inform them of the situation and the corresponding handling measures. After the emergency situation is resolved, summarize the experiences and lessons learned. |

1. Basis for the Topic

Chronic Pelvic Pain Syndrome (CPPS) refers to persistent or recurrent pain in the pelvic region lasting more than three months, without confirmed infection or other obvious local pathological causes to explain the pain. It is often associated with negative cognitive, behavioral, sexual, and emotional consequences, as well as symptoms suggesting lower urinary tract (LUT), sexual, intestinal, pelvic floor, or gynecological dysfunction^[1]^. In men, the global prevalence of CPPS ranges from 2% to 16%^[2]^; while in women, the global prevalence of CPPS can be as high as 24%^[3]^. A series of pelvic floor functional disorders, such as chronic pelvic pain syndrome, have diverse and difficult-to-discuss symptoms that can severely impact patients' social activities and quality of life, leading to issues such as low self-esteem and depression, thereby increasing pressure on families and society^[4]^. One statistic estimates that the annual cost of treating CPPS is approximately $880 million^[5]^.

The exact cause and pathogenesis of Chronic Pelvic Pain Syndrome (CPPS) remain unclear; it may be the end result of the interaction between psychological disorders and multi-system dysfunction ^[6]^. Some studies suggest that chronic pelvic pain syndrome is a manifestation of pelvic floor dysfunction, and its occurrence and development are related to pelvic floor neuromuscular injury ^[7]^. In 85% of patients, the chronic pain syndrome originates from muscle ^[8]^. Muscle overactivity is a characteristic of chronic pelvic pain syndrome and is a major cause of pain ^[9]^. The symptoms of CPPS can severely affect the psychological state of patients, leading to issues such as anxiety, depression, and pain catastrophizing ^[10]^. Psychosocial factors not only directly lead to the occurrence and development of mental disorders but also influence the outcome of somatic diseases. Psychological factors have always been considered relevant in the maintenance of persistent pelvic and urogenital pain ^[11]^. The United Nations Pain Association describes chronic pain as an "unpleasant sensory and emotional experience," which requires psychosocial treatment. This treatment model recognizes the impact of mind-body regulation on pain ^[12]^.

Patients with Chronic Pelvic Pain Syndrome (CPPS) often exhibit hypersensitivity in both the central and peripheral nervous systems, leading to dysfunction in pain regulation, which can exacerbate pain^[13-18]^. Currently, treatment for pelvic pain is primarily based on its etiology and often involves physical therapy, medication, and nerve blocks. For refractory cases, neuromodulation can be applied to the central and peripheral nervous systems^[19]^. Two systematic reviews have evaluated the effects of neuromodulation techniques on CPPS. Both studies concluded that neuromodulation may be effective in alleviating pain and improving the quality of life for patients with CPPS^[20,21]^. Magnetic stimulation can generate small electrical currents in deep neural structures to non-invasively activate these structures (it can be used to detect nerve stimulation and serves as a non-invasive clinical tool for modulating and treating neural function). Some studies have reported that applying 8000 pulses of magnetic stimulation to painful areas in patients with localized musculoskeletal injuries can reduce pain^[22]^. Other research indicates that magnetic stimulation of the sacral nerve roots can significantly improve pudendal neuralgia and sciatica^[23]^. One study showed that magnetic stimulation with specific parameters applied to the peripheral nerves in the pelvic region can inhibit or activate neural pathways and modulate abnormal reflex arcs, thereby affecting the functions of the urethra, bladder, rectum, anus, and pelvic floor muscles^[24]^. Transcranial magnetic stimulation (TMS) is rapidly gaining popularity due to its specificity in modulating brain function and its status as a painless, safe, and reliable treatment method. Repetitive TMS (rTMS) therapy is used globally for patients with depression, with high-frequency rTMS (HF-rTMS) applied to the left dorsolateral prefrontal cortex (DLPFC) showing clear antidepressant effects^[25]^. The U.S. Food and Drug Administration (FDA) first approved this application on December 16, 2008; on the other hand, the benefits of rTMS have been reported to be advantageous for mood in elderly samples^[26]^. Low-frequency rTMS (LF-rTMS) applied to the right DLPFC significantly reduces generalized anxiety, improves self-reported emotional regulation, and enhances sleep quality^[27-29]^.

Research indicates that repetitive peripheral magnetic stimulation (rPMS) may be effective in treating chronic pelvic pain syndrome (CPPS), while repetitive transcranial magnetic stimulation (rTMS) has therapeutic effects on anxiety and depression. However, the articles on peripheral magnetic stimulation for CPPS are mostly observational studies or small sample studies, and there is currently a lack of high-quality research on the treatment of chronic pelvic pain syndrome with peripheral magnetic stimulation. Additionally, the presence of comorbid psychological disorders has not been taken into account. Therefore, we conducted this study to evaluate the efficacy and safety of rPMS combined with rTMS in CPPS patients with comorbid psychological disorders, and to provide more treatment options for CPPS patients with such comorbidities.

References:

[1] D. Engeler, A.P. Baranowski, B. Berghmans, J. Birch, J. Borovicka, A.M. Cottrell, P. Dinis-Oliveira, S. Elneil, J. Hughes, E.J. Messelink, R.A. Pinto, M.L.v. Poelgeest, V. Tidman, A.C.d.C. Williams, P. Abreu-Mendes, S. Dabestani, B. Parsons, J. Tornic, V. Zumstein, EAU Guidelines on Chronic Pelvic Pain, 2022.

[2] C. Smith, Male chronic pelvic pain: An update, Indian Journal of Urology 32(1) (2016).

[3] P. Latthe, M. Latthe, L. Say, M. Gülmezoglu, K.S. Khan, WHO systematic review of prevalence of chronic pelvic pain: a neglected reproductive health morbidity, BMC Public Health 6(1) (2006).

[4] J. Kwon, H.J. Lee, J.H. Joo, E.C. Park, Urinary incontinence status changes and depressive symptoms among middle-aged and older women: Using data from a survey of the Korean Longitudinal Study of Aging, Journal of affective disorders 279 (2021) 549-553.

[5] W. Stones, Y. Cheong, F.M. Howard, S. Singh, Interventions for treating chronic pelvic pain in women(Review), Cochrane Collab 11 (2010) 1-43.

[6] K. Grinberg, Y. Sela, R. Nissanholtz-Gannot, New Insights about Chronic Pelvic Pain Syndrome (CPPS), International Journal of Environmental Research and Public Health 17(3005) (2020).

[7] P. Enck, D.B. Vodusek, Electromyography of pelvic floor muscles, Journal of electromyography and kinesiology : official journal of the International Society of Electrophysiological Kinesiology 16(6) (2006) 568-77.

[8] A.J. Slomski, How groups successfully manage pain patients, Medical economics 73(1) (1996) 112, 115-6, 119-20 passim.

[9] G.A. Santoro, A.P. Wieczorek, C.I. Bartram, Pelvic Floor Disorders Imaging and Multidisciplinary Approach to Management, Springer-Verlag Milan, Berlin, Germany, 2010.

[10] D.A. Tripp, Managing psychosocial correlates of urologic chronic pelvic pain syndromes: Advice from a urology pain psychologist, Canadian Urological Association journal = Journal de l'Association des urologues du Canada 12(6 Suppl 3) (2018) S175-s157.

[11] C. Dybowski, B. Löwe, C. Brünahl, Predictors of pain, urinary symptoms and quality of life in patients with chronic pelvic pain syndrome (CPPS): A prospective 12-month follow-up study, Journal of psychosomatic research 112 (2018) 99-106.

[12] S.N. Raja, D.B. Carr, M. Cohen, N.B. Finnerup, H. Flor, S. Gibson, F.J. Keefe, J.S. Mogil, M. Ringkamp, K.A. Sluka, X.J. Song, B. Stevens, M.D. Sullivan, P.R. Tutelman, T. Ushida, K. Vader, The revised International Association for the Study of Pain definition of pain: concepts, challenges, and compromises, Pain 161(9) (2020) 1976-1982.

[13] L. Lowenstein, M.P. FitzGerald, K. Kenton, L. Hatchett, R. Durazo-Arvizu, E.R. Mueller, K. Goldman, L. Brubaker, Evaluation of urgency in women, with a validated Urgency, Severity and Impact Questionnaire (USIQ), Int Urogynecol J Pelvic Floor Dysfunct 20(3) (2009) 301-7.

[14] L. Lowenstein, Y. Vardi, M. Deutsch, M. Friedman, I. Gruenwald, M. Granot, E. Sprecher, D. Yarnitsky, Vulvar vestibulitis severity--assessment by sensory and pain testing modalities, Pain 107(1-2) (2004) 47-53.

[15] J.J. van Lankveld, M. Granot, W.C. Weijmar Schultz, Y.M. Binik, U. Wesselmann, C.F. Pukall, N. Bohm-Starke, C. Achtrari, Women's sexual pain disorders, The journal of sexual medicine 7(1 Pt 2) (2010) 615-31.

[16] C. Allaire, C. Williams, S. Bodmer-Roy, S. Zhu, K. Arion, K. Ambacher, J. Wu, A. Yosef, F. Wong, H. Noga, S. Britnell, H. Yager, M.A. Bedaiwy, A.Y. Albert, S. Lisonkova, P.J. Yong, Chronic pelvic pain in an interdisciplinary setting: 1-year prospective cohort, Am J Obstet Gynecol 218(1) (2018) 114.e1-114.e12.

[17] J. Thomtén, A. Karlsson, Psychological factors in genital pain: The role of fear-avoidance, pain catastrophizing and anxiety sensitivity among women living in Sweden, Scandinavian journal of pain 5(3) (2014) 193-199.

[18] K. Grinberg, I. Weissman-Fogel, L. Lowenstein, L. Abramov, M. Granot, How Does Myofascial Physical Therapy Attenuate Pain in Chronic Pelvic Pain Syndrome?, Pain research & management 2019 (2019) 6091257.

[19] C.B. Patel, A.A. Patel, S. Diwan, The Role of Neuromodulation in Chronic Pelvic Pain: A Review Article, Pain physician 25(4) (2022) E531-e542.

[20] A.M. Cottrell, M.P. Schneider, S. Goonewardene, Y. Yuan, A.P. Baranowski, D.S. Engeler, J. Borovicka, P. Dinis-Oliveira, S. Elneil, J. Hughes, B.J. Messelink, C.W.A.C. de, Benefits and Harms of Electrical Neuromodulation for Chronic Pelvic Pain: A Systematic Review, European urology focus 6(3) (2020) 559-571.

[21] M. Tutolo, E. Ammirati, J. Heesakkers, T.M. Kessler, K.M. Peters, T. Rashid, K.D. Sievert, M. Spinelli, G. Novara, F. Van der Aa, D. De Ridder, Efficacy and Safety of Sacral and Percutaneous Tibial Neuromodulation in Non-neurogenic Lower Urinary Tract Dysfunction and Chronic Pelvic Pain: A Systematic Review of the Literature, European urology 73(3) (2018) 406-418.

[22] J. Pujol, A. Pascual-Leone, C. Dolz, E. Delgado, J.L. Dolz, J. Aldomà, The effect of repetitive magnetic stimulation on localized musculoskeletal pain, Neuroreport 9(8) (1998) 1745-8.

[23] T. Sato, H. Nagai, Sacral magnetic stimulation for pain relief from pudendal neuralgia and sciatica, Dis Colon Rectum 45(2) (2002) 280-2.

[24] S. Wu, X. Sun, X. Liu, J. Li, X. Yang, Y. Bao, H. Yu, Clinical Observations of Percutaneous Tibial Nerve Stimulation Combined with Sacral Nerve Root Magnetic Stimulation for the Treatment of Male Chronic Pelvic Pain and Chronic Prostatitis, Arch Esp Urol 75(10) (2022) 813-818.

[25] J.P. Lefaucheur, A. Aleman, C. Baeken, D.H. Benninger, J. Brunelin, V. Di Lazzaro, S.R. Filipović, C. Grefkes, A. Hasan, F.C. Hummel, S.K. Jääskeläinen, B. Langguth, L. Leocani, A. Londero, R. Nardone, J.P. Nguyen, T. Nyffeler, A.J. Oliveira-Maia, A. Oliviero, F. Padberg, U. Palm, W. Paulus, E. Poulet, A. Quartarone, F. Rachid, I. Rektorová, S. Rossi, H. Sahlsten, M. Schecklmann, D. Szekely, U. Ziemann, Evidence-based guidelines on the therapeutic use of repetitive transcranial magnetic stimulation (rTMS): An update (2014-2018), Clin Neurophysiol 131(2) (2020) 474-528.

[26] A. Dardenne, C. Baeken, C.L. Crunelle, C. Bervoets, F. Matthys, S.C. Herremans, Accelerated HF-rTMS in the elderly depressed: A feasibility study, Brain Stimul 11(1) (2018) 247-248.

[27] G.J. Diefenbach, L.B. Bragdon, L. Zertuche, C.J. Hyatt, L.S. Hallion, D.F. Tolin, J.W. Goethe, M. Assaf, Repetitive transcranial magnetic stimulation for generalised anxiety disorder: a pilot randomised, double-blind, sham-controlled trial, The British journal of psychiatry : the journal of mental science 209(3) (2016) 222-8.

[28] G.J. Diefenbach, M. Assaf, J.W. Goethe, R. Gueorguieva, D.F. Tolin, Improvements in emotion regulation following repetitive transcranial magnetic stimulation for generalized anxiety disorder, Journal of anxiety disorders 43 (2016) 1-7.

[29] G.J. Diefenbach, L. Rabany, L.S. Hallion, D.F. Tolin, J.W. Goethe, R. Gueorguieva, L. Zertuche, M. Assaf, Sleep improvements and associations with default mode network functional connectivity following rTMS for generalized anxiety disorder, Brain Stimul 12(1) (2019) 184-186.

2. Research Objectives

This study aims to evaluate the effectiveness and safety of repetitive peripheral magnetic stimulation (rPMS) combined with repetitive transcranial magnetic stimulation (rTMS) in the treatment of patients with pelvic pain syndrome accompanied by psychological disorders. By assessing the impact of the interventions on patients' pelvic pain scale/NIH-CPSI scores, depression, anxiety, and stress (DASS-21) scores, as well as the SF-36 quality of life scale scores, and the surface electromyography of the pelvic floor muscles and pudendal nerve motor evoked potentials, the study compares the effects of dual-target magnetic stimulation and peripheral magnetic stimulation on pain levels, psychological scores, and quality of life in patients with chronic pelvic pain syndrome accompanied by psychological disorders. This will provide a basis for clinical treatment options.

3. Research Subjects

Patients who meet the chronic pelvic pain syndrome (CPPS) diagnostic criteria of the European Association of Urology (EAU) guidelines (2022 edition) and have generalized anxiety disorder or major depressive disorder as defined by DSM-5.

3.1 Inclusion Criteria

(1) Meets the diagnostic criteria for Chronic Pelvic Pain Syndrome (CPPS) according to the European Association of Urology (EAU) guidelines (2022 edition).

(2) Age: 18-70 years old;

(3) Can be clearly diagnosed as Generalized Anxiety Disorder or Major Depressive Disorder according to DSM-5 criteria;

(4) No significant pathological changes were observed in the physical examination and auxiliary examinations;

(5) No other treatments except for oral medications within 3 months prior to the visit;

(6) The patient is informed and voluntarily participates in the study.

3.2 Exclusion Criteria

(1) Patients with acute systemic and intracranial hemorrhagic diseases.

Individuals with severe underlying health conditions, such as cardiovascular, liver, kidney, respiratory, and blood diseases, as well as malignant tumors and other progressive diseases.

(3) Patients with cardiac metal membranes, cardiac pacemakers, intracranial metal implants, lumbar metal implants, and implantable electronic devices.

(4) Individuals with infections in the head or sacral region.

(5) Individuals exhibiting unstable vital signs.

(6) Patients who have had adverse reactions to magnetic therapy.

Individuals with atypical autonomic reflexes.

(8) Patients with cognitive impairments who cannot cooperate.

(9) Pregnant or breastfeeding women.

(10) Patients with a history of diseases that lead to peripheral nerve damage.

(11) Patients with serious illnesses, such as malignant effusion, active tuberculosis, cancer, or myasthenia gravis.

(12) Patients with severe mental illness or epilepsy.

3.3 Exit Standards

Participants will be asked to withdraw from the RCT under the following circumstances:

(1) If the patient does not adhere to the prescribed treatment plan or receives alternative treatment;

(2) If the patient experiences severe adverse reactions or changes in their condition that prevent them from continuing to participate in the trial;

(3) If the patient experiences significant organ dysfunction, unstable blood pressure, abnormal heart rate, or difficulty breathing;

(4) If the patient requests to withdraw informed consent due to intolerable adverse reactions or for no specific reason;

(5) If the patient loses follow-up during treatment or follow-up.

4. Plan Design

This study employs a prospective, randomized, blind, controlled trial design.

5. Sample Size Estimation and Grouping

5.1 Sample Size Estimation

According to the research by Yang Jing-ming and his colleagues, under the conditions of β=0.1 and α=0.05, the effect size is 0.5. Pain scores will be used as the primary measurement standard, and G*Power 3.1.9.7 will be used to estimate the sample size. It is calculated that there will be approximately 54 patients in the study cohort, and considering a 10% dropout rate, each group will include 22 people, requiring a total of 66 participants for this RCT.

5.2 Patient Number and Randomization Group

Input patient data into the computer to obtain a subject number. All subject numbers will be three digits long, and patients will consistently use this number throughout the study, which will not be reassigned. Patients who withdraw their informed consent or discontinue the study after their patient number has been assigned will retain their initial number. Randomization will be conducted using a stratified block randomization method, stratified by gender, followed by block randomization within each stratum. Since the purpose of this study is to compare the improvement in symptom scores of chronic pelvic pain as well as anxiety and depression scores, gender is an important influencing factor. Therefore, gender will be used as a stratification factor, and block randomization (with 2 blocks per stratum and a block length of 4) will be employed for grouping. The aforementioned stratified block randomization can be implemented using SPSS software.

6. Research steps or treatment plan

6.1 Clinical intervention measures and steps.

(1) Before treatment: Conduct three examinations and seven comparisons. Researchers will carry out a risk assessment.

(2) Stimulation plan: First conduct rPMS, then conduct rTMS.

(3) Stimulation Site: For peripheral stimulation, the center of the circular coil will be aligned with the S3 region. During the stimulation process, if you feel contractions in the perineal muscles and dorsiflexion responses in the feet, it indicates that the position is correct. For central stimulation, the left DLPFC (l-DLPFC) region will be targeted for patients with major depressive disorder, while the right DLPFC (r-DLPFC) region will be targeted for patients with generalized anxiety disorder.

(4) Stimulation intensity: For peripheral stimulation, 50% of the maximum stimulation intensity will be applied. For central stimulation, the setting will be adjusted to 120% of the resting motor threshold (RMT).

(5) Stimulation parameters: ① Peripheral stimulation: 20 Hz, 2 seconds of stimulation, 28 seconds interval, a total of 1600 pulses. ② r-DLPFC: 1 Hz, 10 seconds of stimulation, 2 seconds interval, a total of 1000 pulses. ③ l-DLPFC: 10 Hz, 4 seconds of stimulation, 26 seconds interval, a total of 3000 pulses.

(6) End of stimulation: The stimulation coil will be moved away from the patient's stimulation site. The patient will be informed that the treatment is complete, and the evaluator will ask if there is any discomfort. If the patient is lying down, they will be instructed to gradually sit up and then stand to avoid falling.

(7) Treatment process: Once a day, 5 days a week, for a total of 4 weeks (20 sessions). The treatment course ends after the 20th session.

6.2 Dual-target Magnetic Stimulation Treatment Procedure

During the treatment process: Determine the stimulation plan and begin peripheral stimulation before central stimulation. Adjust the chair to a horizontal angle of 10-20°, instruct the patient to lie prone on the chair, and align the center of the circular coil with the S3 region, stimulating until a contraction of the anal sphincter or a dorsiflexion response is felt, indicating correct positioning. Once the position is confirmed, use single-pulse stimulation to determine the stimulation intensity, and perform repeated peripheral magnetic stimulation according to the plan. Adjust the chair so that the patient sits upright, and measure the RMT during the initial treatment to determine the patient's magnetic stimulation intensity. The rTMS stimulation intensity is set to 120% of the RMT.

Assisted positioning device: Determine the stimulated cortical location based on the positioning cap; for patients with major depressive disorder, stimulate the left DLPFC, and for patients with generalized anxiety disorder, stimulate the right DLPFC, following the central magnetic stimulation protocol.

6.3 Peripheral Magnetic Stimulation Treatment Protocol

Implement rPMS according to the same procedure as the dual-target magnetic stimulation group. All parameters are the same as those in the dual-target magnetic stimulation group.

6.4 Sham Stimulation Group Treatment Procedure

Both peripheral and central magnetic stimulation use a coil that can produce sound but does not generate a magnetic field. The other parameters and procedures are the same as those in the dual-target magnetic stimulation group.

6.5 Treatment Plan

A treatment course will consist of 20 sessions, with five sessions per week, one session each day. The completion of the 20th treatment will mark the end of the course. Follow-up will be scheduled 8 weeks after the end of the course. During the study, patients may receive oral medication, and the specific medication and dosage must be recorded at the time of enrollment. If there are any significant adjustments to the type and dosage of the medication, they need to actively inform the therapist.

7. Follow-up Plan

(1) Assessment, follow-up plan and content: Patients need to return to the hospital for evaluation before treatment, at the end of each weekly treatment, and 8 weeks after the treatment course ends. The assessment includes the patient's pelvic pain score/NIH-CPSI, DASS-21 scale, SF-36 scoring scale, pelvic floor surface electromyography, and pudendal nerve motor evoked potentials.

(2) Establish a complete database and implement a follow-up mechanism managed by dedicated personnel.

(3) Follow-up time points: Before treatment, at the end of each weekly treatment, and up to 8 weeks after the end of the treatment course.

(4) Outside of the time points for in-hospital assessments and follow-up visits, dedicated follow-up medical staff will conduct tracking follow-ups through phone calls, written questionnaires, emails, and other forms.

8. Evaluation Indicators for Research Results

8.1 Main Efficacy Indicator: Pain Score

(1) Pelvic Pain Rating Scale (for women): It includes the patient's basic information, detailed changes in symptoms, and the effectiveness of previous treatments. It uses pain exacerbated and relieved by sexual activity and menstruation as the starting point for medical history inquiries. Additional details include symptoms related to exercise, urinary, and bowel function.

(2) NIH-CPSI (for men): It mainly consists of three parts, assessing pain or discomfort caused by chronic prostatitis, urinary symptoms, and the impact on quality of life, with a total of nine questions. It is characterized by objectivity and convenience, is quickly accepted by patients, and can provide important references for research and clinical work.

8.2 Secondary Efficacy Indicators:

(1) Anxiety and Depression Scores (DASS-21): For depression, the cutoff values for mild, moderate, and severe depression are 10, 14, and 21, respectively; for the anxiety scale, the cutoff values for mild, moderate, and severe anxiety are 8, 10, and 15, respectively; for the stress scale, the cutoff values for mild, moderate, and severe stress are 15, 19, and 26, respectively.

(2) Surface Electromyography of the Pelvic Floor Muscles: Participants will lie in a supine position, with their upper and lower bodies forming an angle of approximately 120°, and their feet naturally rotated outward. An electrode will be placed in the rectum to collect surface electromyography data from the pelvic floor muscles, while abdominal electrodes will monitor abdominal muscle activity. Before the examination, participants will be instructed to urinate and defecate, taught how to properly contract and relax the pelvic floor muscles, and informed about the assessment process. They will learn how to quickly contract and hold the contraction for 10 seconds. Participants will contract and relax their pelvic floor muscles based on verbal prompts to record surface electromyography values.

(3) Pudendal nerve motor evoked potentials: Participants will be instructed to use a glycerin suppository for rectal evacuation and to assume a prone position. A magnetic stimulation coil will be placed 3-5 centimeters lateral to the midline of the S3 plane, stimulating both sides simultaneously at 55-60% of the maximum output intensity. A surface electrode will be placed in the rectum to record the anal sphincter, and a ground electrode will be connected to the wrist. The amplifier's sampling bandwidth is 5-2000 Hz, with a sensitivity of 3 milliseconds per division and an analysis time of 30 milliseconds. Five successful recordings will be made, and the average results will be taken.

(4) SF-36 QoL Scale: A generic quantitative scale consisting of 36 items, covering eight domains including physical functioning, physical role, bodily pain, general health, vitality, social functioning, emotional role, and mental health.

Adverse events occurring after surgery should be recorded using the adverse event report form. If it is a serious adverse event, please fill out the serious adverse event form and report it to the clinical trial leader and the ethics committee within 24 hours. Additionally, please complete the SAE report form.

8.3 Safety Indicators

Blood routine, liver and kidney function tests, and electrocardiogram: Check whether the examination and treatment measures have caused harm to the participants.

9. Safety Evaluation:

9.1 Benefits

According to relevant reports, chronic pelvic pain syndrome has shown a high incidence both domestically and internationally. Patients with this condition who also have mental health disorders have limited treatment options and poor efficacy. Therefore, it is particularly important to find new effective treatment methods for chronic pelvic pain syndrome accompanied by mental health disorders. Currently, with the development of non-invasive neuromodulation technology, transcranial magnetic stimulation has been widely used in the treatment of mental and psychological disorders, and peripheral magnetic stimulation has also shown certain efficacy in pain treatment. Our team, based on clinical experience and literature reports, has adopted dual-target magnetic stimulation to treat patients with chronic pelvic pain syndrome accompanied by mental health disorders, observing its clinical effectiveness and safety. We hope to provide treatment references for more physicians focusing on pelvic pain syndrome and bring health and hope to a large number of patients.

9.2 Risk

(1) If you experience any discomfort during the study, or if there are any new changes in your condition, or any unexpected situations, regardless of...

Whether it is related to the research or not, you should promptly inform your doctor, who will make a judgment and provide appropriate medical treatment.

(2) Transcranial magnetic stimulation has been recommended for the treatment of anxiety and depression by multiple guidelines and consensus both domestically and internationally. A review of our team's previous studies on peripheral magnetic stimulation and transcranial magnetic stimulation shows that only a very small number of patients experienced pain at the stimulation site and transient hearing loss, with no serious adverse reactions. This has confirmed that the trial is safe and effective, with controllable risks.

9.3 Risk Prevention Measures

9.3.1 Guiding Principles:

(1) Prevention is key: Upon discovering cases, report them promptly and actively take effective measures to control the progression of the disease.

(2) Legal Management: Throughout the entire process of clinical trials, relevant laws and regulations must be implemented. Any unexpected events and adverse events should be reported promptly. Legal management should be enforced during the entire control and treatment process, and legal responsibility should be pursued for any violations.

(3) Hierarchical Responsibility: In clinical trials, standardized operating procedures are strictly implemented, and the entire process is monitored and inspected regularly or irregularly to ensure timely detection and prompt treatment.

(4) Rapid Response: Establish early warning and rapid medical response systems, strengthen the reserves of human, material, and financial resources, enhance emergency handling capabilities, and follow the principles of early detection, timely reporting, reliance on science, and decisive measures for prompt and accurate handling.

9.3.2 Organizational Management:

(1) Leadership Organization: Under the leadership of the dean, the Medical Affairs Department is specifically responsible for organizing and implementing the handling of emergencies. It is responsible for supervising the medical services provided by the hospital's medical staff, inspecting the practice of medical personnel, accepting complaints, and providing consultation services. It also cooperates and coordinates with related disputes and their resolution.

(2) Command System: Incorporate the harm to subjects and emergencies in clinical trials into the hospital's rescue operations and normal medical dispute resolution processes.

(3) Daily management work: The office of the clinical trial institution is responsible for daily business management, specifically overseeing business guidance for clinical trials, organizing management and quality control, conducting supervision and inspection, as well as handling daily information communication, organizational coordination, and reporting of emergencies.

(4) Participants: Medical, nursing, technical professionals from various specialized departments, as well as mental health personnel.

9.3.3 Preventive Measures:

(1) Assurance from the Ethics Committee: Before the clinical trial begins, the trial protocol must be reviewed and approved by the Ethics Committee and signed off before implementation; during the clinical trial, any modifications to the trial protocol must be approved by the Ethics Committee before they can be executed; any harm to subjects or serious adverse events that occur during the trial must be reported to the Ethics Committee.

(2) Assurance from the Principal Investigator: The head of the clinical research project should have at least a bachelor's degree and a senior professional title, possess the specialized knowledge and experience required by the clinical trial protocol, be familiar with the materials and literature related to the clinical trial provided by the sponsor, and have the authority to allocate the personnel and equipment necessary for conducting the trial; they should also have the ability to handle adverse events and the responsibility to report such events in a timely manner.

(3) Researcher's Assurance: Familiarity with the standard operating procedures for adverse event reporting; before the clinical trial begins, all rescue equipment and emergency medications are in place in a timely manner to ensure that participants receive immediate treatment in the event of harm or emergencies.

(4) Assurance to the subjects: Subjects must voluntarily participate and have a thorough understanding of the research project; the rights of subjects to protect themselves must always be respected; measures should be taken to respect the privacy of subjects, maintain the confidentiality of their data, and minimize the impact on their physical and mental well-being as well as their personality; after informing the subjects of all aspects of the trial, subjects must voluntarily confirm their consent to participate in the clinical trial process, which must be documented with a signed and dated informed consent form.

(5) Institutional Guarantees: Establish and improve the quality assurance system; ensure the qualifications of principal investigators; guarantee the facilities of various specialized departments; ensure the quality of clinical trial protocols; control the quality of clinical trial data; establish work systems, design specifications, and standard operating procedures that comply with GCP management regulations. Develop emergency plans for subject harm and unexpected events: establish a handling team for subject harm and unexpected events to ensure that subjects or patients receive timely treatment in the event of harm or unexpected incidents during the medical process; prepare and coordinate the prevention of subject harm and unexpected events, on-site control, emergency response, and other resources and technical support.

9.3.4 Adverse Events:

Adverse events (AEs) refer to any factors and incidents that may affect a patient's treatment outcomes, increase the patient's suffering and burden, potentially lead to medical disputes or accidents, and impact the normal operation of medical work and the personal safety of medical staff during clinical diagnosis and treatment activities as well as hospital operations. These events do not necessarily have a causal relationship with the treatment. Therefore, adverse events can be any negative and unexpected signs (including abnormal laboratory findings) and symptoms, or diseases that are temporally related to the use of medical products, regardless of whether they are considered related to the study.

During the research process, disease progression or deterioration (including increased pain and exacerbation of mental health issues due to disease progression) should be considered part of the efficacy evaluation and should not be reported as adverse events or serious adverse events.

During the clinical research period, all adverse events that occur will be reported on the adverse event page of the Case Report Form (CRF). The severity of adverse events will be classified using the "Classification and Grading Standards for Adverse Events in Medical Quality Safety" established according to China's "Medical Quality Management Measures" (https://www.gov.cn/zhengce) and will be reported in detail as required on the CRF.

9.3.4.1 Definition of Serious Adverse Events

Serious Adverse Events (SAE) refer to any adverse medical condition that occurs during the treatment process and meets one of the following criteria:

(1) Lethality (causing death; note: death is the consequence, not the event);

(2) Life-threatening (Note: "Life-threatening" refers to the immediate danger of death to the patient at the time the event occurs, and does not refer to a hypothetical situation where a more severe event could lead to death.)

(3) Leads to hospitalization of the patient or an extended hospital stay;

(4) Resulting in lifelong or severe disabilities/functional impairments;

(5) It is of significant importance in medicine or requires intervention measures to prevent the occurrence of any of the aforementioned consequences.

9.3.4.2 Causal Relationship

The following standards should be used to assess the relationship between adverse events and treatment:

(1) Very likely related: This category refers to adverse events that are considered to be related to clinical research with a high degree of certainty. An adverse event can be deemed "very likely related" if it meets the following criteria:

The occurrence of adverse events is reasonably time-related to the intervention measures.

The known patient disease status, environmental or toxic factors, or other treatments used by the patient cannot reasonably explain the adverse events.

(2) Possibly related (must meet the first two criteria): This category refers to adverse events that are unlikely to be related to this study, but cannot be definitively ruled out as having a connection. An adverse event can be considered "possibly related" if it meets the following criteria:

The occurrence of adverse events is reasonably time-related to the intervention measures.

Adverse reactions may be caused by the patient's disease state, environmental or toxic factors, or other concomitant treatments used by the patient.

(3) Possibly unrelated (must have the first two items): This category applies to adverse events that meet the following criteria:

The occurrence of adverse events is not reasonably correlated in time with the intervention measures.

Adverse events are clearly caused by the patient's disease state, environmental or toxic factors, or other concomitant treatments used by the patient.

(4) Irrelevant: This category refers to adverse events that are clearly and unequivocally determined to be caused solely by external factors (such as diseases, environment, etc.) and do not meet the relevance judgment criteria under "possibly irrelevant," "possibly relevant," or "very likely relevant."

It must comply with the ICH guidelines for the management of clinical trial safety data and the definitions and standards for expedited reporting.

9.3.4.3 Expected Adverse Events

(1) Epileptic seizure;

(2) fainting;

(4) Localized pain, headache, discomfort;

(5) Temporary changes in hearing;

(6) Brief cognitive/neuropsychological changes;

(7) Acute mental changes;

(8) Others.

9.3.4.4 Adverse Event Handling Measures

(1) Take necessary treatment measures based on specific circumstances and decide whether to terminate the clinical trial. Detailed records of the symptoms, signs, or laboratory test results, including the time of occurrence, duration, severity, management measures, and progress, should be documented in the medical record. The researcher should evaluate the relevance to the clinical trial, sign, and date the record.

(2) When adverse events are discovered, researchers should immediately address the situation and report it to the project leader, the ethics committee, and the clinical research center. They should determine the necessary diagnostic and treatment measures based on the patient's condition and decide whether to terminate the clinical trial. All adverse events should be investigated and tracked, with detailed records of the handling process and outcomes maintained until the issue is properly resolved or the patient's condition stabilizes. If there are abnormal test results, they should be monitored until they return to normal. The method of follow-up can be chosen based on the severity of the adverse reaction, including hospitalization, outpatient visits, home visits, phone calls, or correspondence.

(3) Reporting of Adverse Events: During the study period, regardless of the treatment received by the patient, any serious adverse events or serious laboratory test result abnormalities that occur must be documented by the investigator on an adverse event report form and reported to the project leader, ethics committee, and clinical research center within 24 hours of becoming aware of them.

SAE report contact person: Xueqian Li(Chengdu Anorectal Hospital Pelvic Floor Center)

Contact person: Yu Zhang(Academic Committee of Chengdu Anorectal Hospital)

Mobile phone：+86-13608015271

Email：1246649597@qq.com

Address: No. 152, East Daqiang Street, Taisheng South Road, Qingyang District, Chengdu, Sichuan Province.

(4) Recording: Researchers should document the subjects' symptoms, signs, laboratory tests, the time of onset of damage, duration, severity, treatment measures, and progress in the original medical records and CRF forms, ensuring that the records are true, accurate, complete, timely, and legal. They should fill out the serious adverse event report form, sign it, and indicate the date; the original records should include the time, reporting method, and the reporting institution.

(5) Follow-up: Researchers should follow up on all adverse events experienced by the subjects, determining the follow-up time based on the condition. During the follow-up process, necessary interventions and treatments should be provided to ensure that the harm to the subjects is minimized and to fully guarantee their safety. Detailed records of the follow-up process and the outcomes of the interventions should be maintained.

9.3.5 Emergency Response Measures:

(1) Measures for handling public health emergencies:

Report: During normal working days, on-duty medical staff should immediately report any incidents of harm to subjects to the incident handling team. On holidays or at night, they should report immediately to the administrative duty officer, who will then report to the subject harm and incident handling team. The team members should conduct a comprehensive assessment, make a preliminary judgment on the nature of the public health emergency, and report to the higher administrative department, suggesting whether to activate the emergency response plan for public health incidents.

(2) Activate the emergency plan: After approval from the higher health administrative department, activate the emergency plan for public health emergencies, including:

★ Establish special outpatient services, emergency services, and observation rooms.

★ Hospital leadership duty: The hospital office quickly formulated a duty schedule for the hospital leaders, ensuring that one leader is on duty 24 hours a day, fully responsible for emergencies; the medical department has dedicated personnel on duty 24 hours a day; all relevant personnel have their mobile phones on 24 hours a day to ensure smooth communication. Every morning at 8:00 AM and every afternoon at 5:00 PM, gather relevant information at the department, hold a focused discussion, and address related issues.

★ Ensure Material Supply: Urgently mobilize personnel, reserve relevant materials, transportation tools, as well as related facilities and equipment, to ensure the reasonable allocation of medical resources and guarantee the supply of emergency medical materials for key departments.

★ Evacuation or Isolation: Evacuate and isolate personnel as necessary based on the condition.

★ Rescue and Documentation: Medical personnel strictly adhere to protective measures, providing emergency medical care and on-site treatment for patients; they write detailed and complete medical records; if transfer to another hospital is necessary, patients are transferred to the receiving or designated medical institution as per regulations.

★ Training and Drills: Regular training on emergency response knowledge and skills related to the nature of public health emergencies. Drill, always be prepared for emergencies.

(2) Measures for dealing with natural disasters:

In the event of a fire, flood, earthquake, or similar incidents, you should immediately contact "119" or "110" and inform the damage and emergency response team. During holidays or at night, the report should be made to the general duty officer, who will then notify the aforementioned team. The team leader will promptly report to the dean and receive instructions, while the office will announce the emergency situation to the entire institution. Relevant functional departments should carry out their respective duties.

(3) Emergency power and water outage response measures:

Handling measures: In the event of a sudden power outage in a department due to local circuit issues, medical staff should immediately notify the management personnel of the General Affairs Department by phone. After receiving the notification, the logistics management personnel should promptly arrange for professionals to rush to the site for emergency repairs to restore power as soon as possible. Departments should make arrangements in advance for work when notified of water or power outages; relevant inspections or treatments scheduled during that time should be avoided. For specimens that must be tested on the same day as required by the clinical trial protocol, they should be properly preserved while ensuring specimen quality, and testing should be conducted after water or power supply is restored.

9.3.6 Penalties: If medical personnel engage in any of the following behaviors, the relevant department of the hospital shall order corrections, issue a notice of criticism, and give a warning;

Disciplinary actions such as demotion or dismissal shall be imposed on department heads and other directly responsible individuals in accordance with the law; if the consequences result in disability, death, or the spread of disease among subjects (patients), or cause other serious harm to public health, and constitute a crime, criminal responsibility shall be pursued in accordance with the law.

(1) Failure to fulfill the reporting responsibilities for adverse events or emergencies as required, including concealing, delaying, or falsely reporting.

(2) Failure to take timely measures for adverse events or emergencies that occur in subjects as required.

(3) Refusing to see patients when an emergency occurs;

(4) Refusing to comply with hospital work arrangements during emergencies.

10. Recruitment process (including recruitment procedures and start time, recruitment materials, expected number of recruits)

10.1 Recruitment Procedure: This includes three stages: recruiting subjects, screening qualified subjects, and obtaining informed consent from the subjects.

10.1.1 Recruitment of Subjects

(1) Subject recruitment staff: Responsible doctors for patient reception, attending resident physicians, and clinical supervising doctors can also be involved. Once the recruitment personnel are determined, a recruitment plan and requirements will be developed.

(2) Recruitment methods: The first method is for the recruiting personnel to determine that the patient meets the trial requirements and recommend the patient to participate in the trial; the second method is when the patient's attending physician is not a researcher of the trial, the physician determines that the patient meets the clinical trial requirements, inquires about the patient's willingness, and then contacts the researcher to include the patient.

(3) Recruitment locations: hospital outpatient departments, inpatient wards, or health examination centers.

(4) Reception of potential subjects: Set up a dedicated recruitment window with designated personnel responsible for receiving visiting subjects or use a dedicated phone line to answer inquiries from consulting patients.

(5) Make a preliminary judgment on the likelihood of subject inclusion based on the inclusion and exclusion criteria as well as the doctor's clinical experience.

(6) Subject eligibility screening: Provide an overview of the trial to subjects who are initially judged to be potentially eligible, conduct physical examinations or laboratory tests, and based on the results of these examinations, confirm again whether the subjects truly meet the inclusion and exclusion criteria for the trial.

(7) Informed consent of the subjects: A detailed explanation of the clinical trial protocol will be provided to patients who meet the screening criteria, and they will be informed of the potential benefits and risks, ensuring that they are fully informed.

10.1.2 Screening Qualified Subjects

The initial assessment of potentially eligible subjects will be conducted for eligibility determination. A separate, clear, and specific medical history screening form should be designed in advance based on the eligibility criteria. The eligibility criteria involve refining the clinical trial's diagnostic criteria and inclusion/exclusion criteria into assessable items, and excluding special populations (such as pregnant individuals and those with infectious diseases) and patients with related complications based on the disease and trial design. The eligibility criteria will determine whether subjects meet the requirements of the clinical trial; patients who meet the research conditions will enter the clinical trial, while those deemed ineligible will be excluded and not enrolled. Any additional assessment costs incurred for patients who do not meet the requirements should be borne by the research project, and they should receive standard treatment. The specific eligibility determination process is as follows: ① Include subjects who meet the predetermined diagnostic criteria; ② Then include subjects who meet the predetermined inclusion criteria; ③ Finally, exclude patients who exhibit characteristics of the exclusion criteria from those who do not exhibit such characteristics.

10.1.3 Obtaining Informed Consent from Subjects

After passing the eligibility screening, researchers should inform each participant about informed consent and confirm their signature.

10.2 Startup Time

The project will start recruiting participants on January 1, 2024, after obtaining approval and ethics committee approval.

10.3 Recruitment Materials

Informed Consent Form.

10.4 Expected Number of Recruits

66 patients with chronic pelvic pain syndrome accompanied by mental and psychological disorders who meet the selection criteria.

10.5 Time of participation in the study (duration of each participation and total time)

Participants who have been enrolled and received the first week of treatment (5 sessions) are considered to have participated in the study for the first time; participants receive 5 treatments per week for a total of 4 weeks (20 sessions); after the treatment course ends, they return to the hospital 8 weeks later for further examinations and assessments; the total duration of participation in the study ends 8 weeks after the treatment course concludes.

11. Data Management and Statistical Analysis Plan

11.1 Data Processing

11.1.1 Data Collection

All data should be recorded in a timely, truthful, and detailed manner in the Case Report Form (CRF). Researchers must input information into the CRF according to the protocol requirements. The research center will assign a supervisor to check the completeness and accuracy of the CRF and guide the research center staff in making necessary modifications or additions. The CRF will be submitted to data processing by the research supervisor, with one copy retained at the research center and another attached as the supervisor's work document. The CRF will be handed over to two reliable medical data processors for data entry and verification. The case report form should be filled out by a designated person from the unit and signed by the project leader of the unit to be considered a valid case. After the clinical trial is completed, a clinical summary report should be written according to the requirements of the clinical summary guidelines.

11.1.2 Case Report Form (CRF)

In clinical trials, it is necessary to record the specified observations or examination items in the case report form. The case report form includes...

The content must be completely consistent with the original data, and the basis for the results calculated from the original data should be traceable.

When filling out, you must meet the following standards:

(1) Fill in with a black signature pen or black ballpoint pen;

(2) Cases that have already signed the informed consent form and meet the inclusion criteria should fill out the case report form, even if they have not received treatment or are determined to not meet the inclusion criteria after treatment.

(3) When making corrections, the original record must be crossed out with a line (correction fluid or similar cannot be used), but the original record must remain legible. Additionally, the person making the correction must sign at the correction point and indicate the date of the correction.

(4) For unexamined items, it must be marked as "ND (Not Done)".

11.1.3 Database Management and Quality Control

The data items in the CRF will be entered into the research database using a double-entry verification method. Text items (such as comments) can only be manually verified after being entered once from the CRF. Subsequently, data managers conduct systematic checks on the information in the input database using error messages printed from the confirmation procedures and database lists. If necessary, a separate document provides clear instructions for special operations related to the handling and archiving of the DQF (Data Quality Form) for the trial (such as confirmation plans). The database will be locked once it is declared complete and error-free. After that, any changes to the database can only be made with the joint written consent of the clinical research leader, research statistician, and data manager. The interpretation of results will be conducted by a third party who is unaware of the patient grouping, ensuring the objectivity of the results.

11.2 Statistical Analysis

11.2.1 Analysis of Population Definition:

The patients who were enrolled and received treatment constitute the intention-to-treat population (ITT) for this trial. The population and the patients who comply with the requirements of this clinical trial protocol and complete this study constitute the per protocol population (PP). The statistical analysis will use intention-to-treat (ITT) for missing data, comparing the "full analysis set (FAS)" and the "per protocol set (PP)."

11.2.2 Statistical Analysis Methods

Statistical analysis will be conducted using SPSS 27.0 software. Continuous data will be presented as mean ± standard deviation. Paired t-tests will be used for inter-group comparisons, and repeated measures ANOVA will be used for intra-group comparisons, including before and after treatment and during follow-up. Statistical significance will be indicated by a P value &lt; 0.05. If there is a reduction in personnel during the trial, an intention-to-treat (ITT) analysis will be performed, comparing the "full analysis set (FAS)" and the "per-protocol set (PP)."

12. Data Confidentiality Plan

Participation in the trial and personal information during the trial are confidential. Information that can identify the subjects will not be disclosed to anyone outside the research team unless permission is obtained from the subjects. All research members and sponsors are required to keep the identities of the subjects confidential. The subjects' files will be stored in a locked filing cabinet and will only be accessible to researchers. To ensure that the research is conducted according to regulations, government regulatory agencies or members of the ethics review committee may review the subjects' personal information at the research site as necessary. When the results of this study are published, no personal information about the subjects will be disclosed.

13. Ethical Requirements and Informed Consent

13.1 Obtain approval from the ethics committee

Before initiating clinical trials at a research institution, approval from the institution's ethics committee must be obtained. During the trial, researchers are required to submit progress reports to the ethics committee, and after the trial concludes, a written report should also be provided to the ethics committee.

13.2 Informed Consent

Before the case registration, the principal investigator or researchers explain the relevant content of the clinical trial to the subjects according to the informed consent form, and then provide the subjects with sufficient time to consider it. The subjects then independently decide whether to sign the informed consent form. The principal investigator or researchers and the subjects sign the informed consent form and indicate the date (specifying the date of obtaining informed consent). If trial-related personnel provide additional explanations, they must also sign or write their name and stamp it, and record the date of the explanation. Furthermore, if there are changes to the trial protocol during the trial, or if new information arises that may affect the subjects' decision to continue participating in the trial, the subjects must be informed of the changes, and they must again independently decide whether to sign a new informed consent form. If the subjects do not agree to sign the new informed consent form, they must withdraw from the clinical trial. The informed consent form is made in duplicate, with the original kept by the center and a copy retained by the subjects.

13.3 Revision of the Informed Consent Form

The principal investigator may consider modifying the informed consent form based on the latest information. The revised informed consent form must be approved by the ethics committee before it can be used in the clinical trial. Patients who have signed the old informed consent form and have not completed the clinical trial should sign the new informed consent form again before continuing with the trial. Both the new and old versions of the informed consent form must be retained as original documents.

13.4 Protection of Participants' Privacy

In case report forms, statistical analysis reports, clinical trial reports, and adverse event reports, any content that involves patient information must not include the patient's real information. Patients can be identified through numbers and initials.

13.5 Modification of the Research Plan

This plan, after being approved by the ethics committee, requires that if there are significant modifications during the implementation process, the clinical research leader must write a "Protocol Amendment Document" and sign it. Additionally, it must be submitted for approval by the ethics committee before implementation. If there are no fundamental changes, the clinical research team, statisticians, and methodologists will discuss and decide together and sign.

14. Quality Control and Quality Assurance

14.1 Data Management

All selected cases must have the CRF (Case Report Form) completed by a designated physician, and all items in the CRF must be filled out. The filled items must not be altered; if a correction is needed, the incorrect data should be crossed out with a short horizontal line, and the correct result should be written above it, along with a signature and the date of the correction. After completing the form, the physician should carefully check and verify all data in the CRF and sign the form to indicate that it has been reviewed, ensuring the authenticity and accuracy of the data filled in. The completed CRF will be reviewed by the monitor and then kept for data entry and management.

14.2 Quality Control of This Study

Control content: Check if there are any omissions in the filling of indicators, whether it is consistent with the original data, and whether it is filled out accurately.

Frequently Asked Questions: Low consistency in completion by different doctors; missing or incorrect entries, and inconsistencies with research cases.

Solution: Before the start of this study, the participating doctors and staff must undergo standardized training, with uniform recording methods and judgment criteria. Ensure the consistency, reliability, completeness, and accuracy of the evaluation indicators. During the study, monitors appointed by the Principal Investigator (PI) will conduct regular monitoring visits and audits to ensure strict adherence to all aspects of the study protocol, the correctness of the Case Report Form (CRF) completion, and the authenticity and accuracy of the data recorded.

14.3 Preservation of Original Data

The original data of this study, including signed informed consent forms, relevant clinical scales, laboratory test reports, functional examination reports, case records, and other related records, should be kept in the archives of Chengdu Anorectal Specialty Hospital.

14.4 Setting up the experimental blind method

A researcher will place the random sequence numbers generated by SPSS software into opaque envelopes separately and number the envelopes in order on the surface. This researcher will not participate in other studies and will ensure that the envelopes containing the random sequence numbers remain sealed throughout the research process, so that other personnel will not know the random sequence numbers inside the sealed envelopes in advance. A second researcher will select eligible patients for the study based on inclusion and exclusion criteria. Patients will be informed of the potential benefits and risks of the study, and if they agree to participate, they will sign an informed consent form. The third researcher will ask patients about their condition, assess their status, assist in filling out the CRF forms, and distribute the envelopes to the patients participating in the study. The fourth researcher will treat patients according to the random sequence numbers in the envelopes given to them and will not participate in other studies. The fifth researcher will evaluate and complete the pelvic pain scale (for women) / National Institutes of Health Chronic Prostatitis Symptom Index (NIH-CPSI) (for men), the DASS-21 scale, and the SF-36 quality of life scale assessments, and will complete the patients' pelvic floor surface electromyography and pudendal nerve motor evoked potential tests. This researcher will not participate in data analysis or other studies. After data collection is completed, the first unblinding will occur, with two researchers entering and verifying the data separately. Finally, a statistical analyst will conduct the statistical analysis, comparing the three groups without knowing the group assignments. After the analysis is completed, a second unblinding will take place, marking the end of the study.

15. Preservation of Data and Records

Researchers are responsible for ensuring that research records are not accidentally damaged; the medical records of subjects should be clearly labeled to prevent accidental destruction of documents due to errors. Researchers and research institutions must retain clinical trial-related materials for five years after the conclusion of the clinical trial. Original medical documents should be kept according to the relevant regulations of the hospital, but must not be less than five years after the conclusion of the clinical trial.

16. Research Process

All subjects must meet the diagnostic criteria for CPPS according to EAU and the diagnostic criteria for generalized anxiety disorder or major depressive disorder according to DSM-5. Subjects who are determined to meet the inclusion criteria and do not fall under the exclusion criteria are considered potential subjects. The screening physician will inform the subjects and their families about the study, explain the differences in treatment, and seek the opinions of the subjects and their families on whether they agree to random grouping. Patients who agree to random grouping will sign an informed consent form and will then be included in the study for random grouping and will receive follow-up after the treatment course. (See Appendix 1)

17. Summary report and paper publication

(1) After completing the research summary of the clinical study, papers can be published and participation in domestic and international academic conferences is possible.

(2) The results of this research will be published in the form of papers. It is expected to publish 1-2 SCI papers.

**Appendix 1**

**Research Treatment Chart**

**
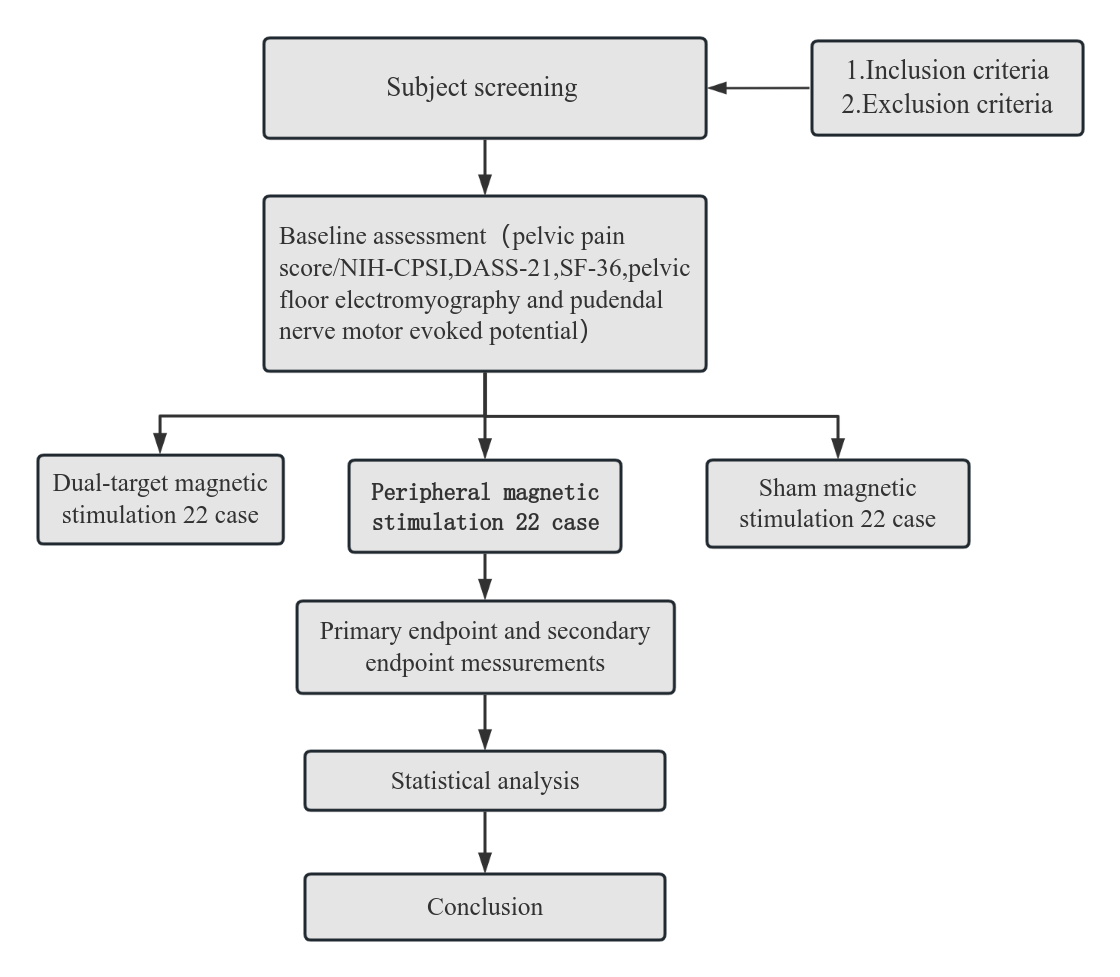
**

**Appendix 2**

Table 1 SPIRIT schedule

| Research Phase  Project Name | Before Treatment | First Week Treatment | Second Week Treatment | Third Week Treatment | Fourth Week Treatment | 8 Weeks After Treatment |
| --- | --- | --- | --- | --- | --- | --- |
|  | t0 | t1(5days) | t2(5days) | t3(5days) | t4(5days) | t5 |
| Screening for Enrollment | √ |  |  |  |  |  |
| Medical history and basic information | √ |  |  |  |  |  |
| Routine examinations | √ |  |  |  | √ |  |
| Informed Consent Form | √ |  |  |  |  |  |
| Drug treatment | √ | √ | √ | √ | √ | √ |
| Pelvic Pain Scale/NIH-CPSI Score | √ | √ | √ | √ | √ | √ |
| DASS-21Score | √ | √ | √ | √ | √ | √ |
| SF-36 Quality of Life Scale | √ | √ | √ | √ | √ | √ |
| Surface electromyography of the pelvic floor muscles | √ | √ | √ | √ | √ | √ |
| Pudendal nerve motor evoked potentials | √ | √ | √ | √ | √ | √ |
| Bilateral magnetic stimulation |  | √ | √ | √ | √ |  |
| Peripheral magnetic stimulation |  | √ | √ | √ | √ |  |
| Fake stimulus |  | √ | √ | √ | √ |  |
| Follow-up |  |  |  |  |  | √ |
| Adverse Event |  | √ | √ | √ | √ | √ |
| Analysis of missing items and non-respondents. |  |  |  |  |  | √ |

Note: Routine examinations include: complete blood count, liver function, kidney function, and electrocardiogram examination. Pelvic pain scale, Female Pelvic Pain Scoring Scale; NIH-CPSI, The National Institutes of Health Chronic Prostatitis Symptom Index (NIH-CPSI); SF-36,SF-36 scale of quality of life; t0, enrollment; t1, At the end of 5 sessions;t2,At the end of 10 sessions; t3,At the end of 15 sessions; t4,At the end of 20 sessions; t5, Follow-up visit 8 weeks after treatment ends.

**Appendix 3**

**Table 2 Pelvic Pain Score Sheet**

Number： Name：

ID： Tel：

Pain characteristics

1. Please describe your pain situation, such as frequency, duration, and intensity of the pain.

1. What do you think is the cause of the pain?

3、Has there been any event related to the onset of pain? A.Yes B.No

If so, what is the related event?

1. How long has this been going on?
2. The following symptoms are graded based on the severity of the past week. Please √ the appropriate box.

| Symptoms | 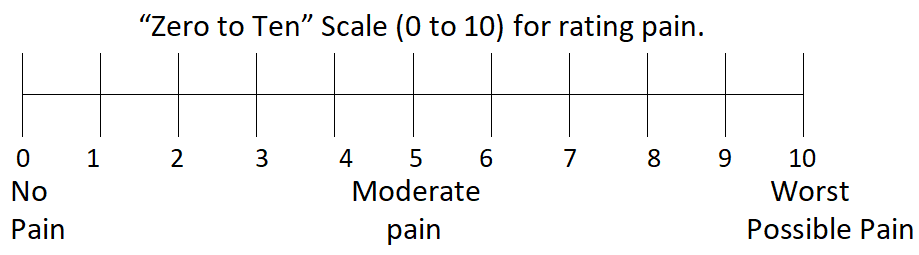 | | | | | | | | | | |
| --- | --- | --- | --- | --- | --- | --- | --- | --- | --- | --- | --- |
|  | 0 | 1 | 2 | 3 | 4 | 5 | 6 | 7 | 8 | 9 | 10 |
| How do you evaluate pain? |  |  |  |  |  |  |  |  |  |  |  |
| Ovulation pain (mid-menstrual cycle) |  |  |  |  |  |  |  |  |  |  |  |
| Premenstrual abdominal pain |  |  |  |  |  |  |  |  |  |  |  |
| Premenstrual pain (no spasms) |  |  |  |  |  |  |  |  |  |  |  |
| Deep dyspareunia |  |  |  |  |  |  |  |  |  |  |  |
| Groin pain when lifting leg |  |  |  |  |  |  |  |  |  |  |  |
| Post-coital pain lasting hours or days |  |  |  |  |  |  |  |  |  |  |  |
| Pain when holding urine |  |  |  |  |  |  |  |  |  |  |  |
| Muscle/joint pain |  |  |  |  |  |  |  |  |  |  |  |
| Menstrual cramping pain |  |  |  |  |  |  |  |  |  |  |  |
| Pain disappears after menses |  |  |  |  |  |  |  |  |  |  |  |
| Burning pain in the vagina after sex |  |  |  |  |  |  |  |  |  |  |  |
| painful urination |  |  |  |  |  |  |  |  |  |  |  |
| backache |  |  |  |  |  |  |  |  |  |  |  |
| migraines |  |  |  |  |  |  |  |  |  |  |  |
| seating pain |  |  |  |  |  |  |  |  |  |  |  |
| Overall score |  | | | | | | | | | | |

**Appendix 4 Table 3 NIH-CPSI**

Number： Name：

ID： Tel：

The following symptoms are graded based on the severity of the past week. Please √ the appropriate box.

| **Pain or discomfort symptoms** | | None | Rarely | Occasionally | Frequently | Very common | Almost always |
| --- | --- | --- | --- | --- | --- | --- | --- |
| 1. Have you experienced pain or discomfort in the following areas? | | 0 | 1 | 2 | 3 | 4 | 5 |
| Perineal pain | |  |  |  |  |  |  |
| Didymalgia | |  |  |  |  |  |  |
| Glans penis | |  |  |  |  |  |  |
| Lumbosacral and suprapubic region | |  |  |  |  |  |  |
| 1. Pain or burning sensation during urination | |  |  |  |  |  |  |
| 1. Painful discomfort at or after ejaculation | |  |  |  |  |  |  |
| 1. Use numbers to describe the degree of pain or discomfort mentioned above.   No pain 0 1 2 3 4 5 6 7 8 9 10 worst possible pain | | | | | | | |
| **Urinary symptoms** | | None | Less than 1/5 | Less than half | About half | More than half | Almost every time |
|  |  | 0 | 1 | 2 | 3 | 4 | 5 |
| 1. Whether there is often a feeling of incomplete urination at the end of urination ? | |  |  |  |  |  |  |
| 1. Do you often feel the need to urinate again within 2 hours after urinating? | |  |  |  |  |  |  |
| **Symptom severity** | | None | Little | Some | A lot |  |  |
|  |  | 0 | 1 | 2 | 3 |  |  |
| 1. Do the above symptoms affect your daily life? | |  |  |  |  |  |  |
| 1. Are you always reminded of your symptoms? | |  |  |  |  |  |  |
| Quality of life | Very satisfactory | Satisfactory | Good | Just so so | Mostly unsatisfactory | Unhappy | Fearful |
|  | 0 | 1 | 2 | 3 | 4 | 5 | 6 |
| If left untreated, how do you think your life will be in the future? |  |  |  |  |  |  |  |
| Analysis of NIH-CPSI score results | | | | | | | |
| Pain and discomfort score:1+2+3+4=( ) | | Symptom impact scores on quality of life：7+8+9=( ) | | | | | |
| Voiding Symptom Score：5+6=( ) | |  | | | | | |
| Symptom severity：1+2+3+4+5+6=( ) | | Mild:0~9 | | Moderate:10~18 | | Severe:19~31 | |
| Overall rating:  1+2+3+4+5+6+7+8+9=( ) | | Mild:1~14 | | Moderate:15~29 | | Severe:30~43 | |

**Appendix 5**

# Table 4 Depression Anxiety and Stress Scale(DASS-21)

Number： Name：

ID： Tel：

(The subscale scores were multiplied by 2 to give a score for that subscale, with higher scores representing more of that emotion.)

| Please read each statement and indicate the extent to which it applies to you in the past week by checking the appropriate box. There are no right or wrong answers. Do not spend too much time on any statement.The scoring criteria are as follows: 0 Does not apply to me at all 1 Applies to me to some extent, or applies sometimes 2 Applies to me to a onsiderable extent, or applies most of the time 3 Applies to me very much, or applies most of the time | | | | | | | | | | | |
| --- | --- | --- | --- | --- | --- | --- | --- | --- | --- | --- | --- |
|  |  | | | | 0 | 1 | | 2 | | 3 | score |
| 1 | I couldn’t seem to experience any positive feeling at all | | | |  |  | |  | |  |  |
| 2 | I found it difficult to work up the initiative to do things | | | |  |  | |  | |  |  |
| 3 | I felt that I had nothing to look forward to | | | |  |  | |  | |  |  |
| 4 | I felt down-hearted and blue | | | |  |  | |  | |  |  |
| 5 | I was unable to become enthusiastic about anything | | | |  |  | |  | |  |  |
| 6 | I felt I wasn’t worth much as a person | | | |  |  | |  | |  |  |
| 7 | I felt that life was meaningless | | | |  |  | |  | |  |  |
| Depression Scale | | | | | | | | Total score | | |  |
| 8 | I was aware of dryness of my mouth | | | |  |  | |  | |  |  |
| 9 | I experienced breathing difficulty (e.g. excessively rapid breathing, breathlessness in the absence of physical exertion) | | | |  |  | |  | |  |  |
| 10 | I experienced trembling (e.g. in the hands) | | | |  |  | |  | |  |  |
| 11 | I was worried about situations in which I might panic and make a fool of myself | | | |  |  | |  | |  |  |
| 12 | I felt I was close to panic | | | |  |  | |  | |  |  |
| 13 | I was aware of the action of my heart in the absence of physical  exertion (e.g. sense of heart rate increase, heart missing a beat) | | | |  |  | |  | |  |  |
| 14 | I felt scared without any good reason | | | |  |  | |  | |  |  |
| Anxiety Scale | | | | | | | | Total score | | |  |
| 15 | I found it difficult to relax | | | |  |  | |  | |  |  |
| 16 | I tended to over-react to situations | | | |  |  | |  | |  |  |
| 17 | I felt that I was using a lot of nervous energy | | | |  |  | |  | |  |  |
| 18 | I felt that I was rather touchy | | | |  |  | |  | |  |  |
| 19 | I found it hard to wind down | | | |  |  | |  | |  |  |
| 20 | I was intolerant of anything that kept me from getting on with what I  was doing | | | |  |  | |  | |  |  |
| 21 | I found myself getting agitated | | | |  |  | |  | |  |  |
| Stress Scale | | | | | | | | Total score | | |  |
| Recommended cut-off scores for conventional severity labels (normal, moderate, severe) are as follows: NB Scores on the DASS-21 will need to be multiplied by 2 to calculate the final score. | | | | | | | | | | | |
|  | | Normal | Mild | Moderate | | | Severe | | Extremely Severe | | |
| Depression | | 0-9 | 10-13 | 14-20 | | | 21-27 | | 28+ | | |
| Anxiety | | 0-7 | 8-9 | 10-14 | | | 15-19 | | 20+ | | |
| Stress | | 0-14 | 15-18 | 19-25 | | | 26-33 | | 34+ | | |
| Lovibond, S.H. & Lovibond, P.F. (1995). Manual for the Depression Anxiety & Stress Scales. (2nd Ed.)Sydney: Psychology Foundation. | | | | | | | | | | | |

**Appendix 6**

# Table 5 36-Item Short Form Survey

Number： Name：

ID： Tel：

There are a total of 36 questions below, and after each question, there are several answers to choose from. Please fill in the corresponding score in the score column of the table for the answers you think are appropriate.

| Item |  | | | | |  | | | | | |  | | | | | |  | | | | |  | | | Score |
| --- | --- | --- | --- | --- | --- | --- | --- | --- | --- | --- | --- | --- | --- | --- | --- | --- | --- | --- | --- | --- | --- | --- | --- | --- | --- | --- |
| 1. In general, would you say your health is | Excellent | | | | | Very good | | | | | | Good | | | | | | Fair | | | | | Poor | | |  |
|  | 5 | | | | | 4.4 | | | | | | 3.4 | | | | | | 2 | | | | | 1 | | |  |
| 1. **Compared to one year ago**, how would you rate your health in genera **now**? | Much better | | | | | Somewhat better | | | | | | About the same | | | | | | Somewhat worse | | | | | Much worse | | |  |
|  | 1 | | | | | 2 | | | | | | 3 | | | | | | 4 | | | | | 5 | | |  |
| The following items are about activities you might do during a typical day. Does **your health now limit you** in these activities? If so, how much? | | | | | | | | | | | | Yes, limited a lot | | | | Yes, limited a little | | | | | | No, not limited at all | | | |  |
| 1. **Vigorous activities**, such as running, lifting heavy objects, participating in strenuous sports | | | | | | | | | | | | 1 | | | | 2 | | | | | | 3 | | | |  |
| 1. **Moderate activities**, such as moving a table, pushing a vacuum cleaner, bowling, or playing golf | | | | | | | | | | | | 1 | | | | 2 | | | | | | 3 | | | |  |
| 1. Lifting or carrying groceries | | | | | | | | | | | | 1 | | | | 2 | | | | | | 3 | | | |  |
| 1. Climbing **several** flights of stairs | | | | | | | | | | | | 1 | | | | 2 | | | | | | 3 | | | |  |
| 1. Climbing **one** flight of stairs | | | | | | | | | | | | 1 | | | | 2 | | | | | | 3 | | | |  |
| 1. Bending, kneeling, or stooping | | | | | | | | | | | | 1 | | | | 2 | | | | | | 3 | | | |  |
| 1. Walking **more than a mile** | | | | | | | | | | | | 1 | | | | 2 | | | | | | 3 | | | |  |
| 1. Walking **several blocks** | | | | | | | | | | | | 1 | | | | 2 | | | | | | 3 | | | |  |
| 1. Walking **one block** | | | | | | | | | | | | 1 | | | | 2 | | | | | | 3 | | | |  |
| 1. Bathing or dressing yourself | | | | | | | | | | | | 1 | | | | 2 | | | | | | 3 | | | |  |
| During the **past 4 weeks**, have you had any of the following problems with your work or other regular daily activities **as a result of your physical health**? | | | | | | | | | | | | | | | | | | | No | | | | Yes | | |  |
| 1. Cut down the **amount of time** you spent on work or other activities | | | | | | | | | | | | | | | | | | | 1 | | | | 2 | | |  |
| 1. **Accomplished less** than you would like | | | | | | | | | | | | | | | | | | | 1 | | | | 2 | | |  |
| 1. Were limited in the **kind** of work or other activities | | | | | | | | | | | | | | | | | | | 1 | | | | 2 | | |  |
| 1. Had **difficulty** performing the work or other activities (for example, it took extra effort) | | | | | | | | | | | | | | | | | | | 1 | | | | 2 | | |  |
| During the **past 4 weeks**, have you had any of the following problems with your work or other regular daily activities **as a result of any emotional problems** (such as feeling depressed or anxious)? | | | | | | | | | | | | | | | | | | | No | | | | Yes | | |  |
| 1. Cut down the **amount of time** you spent on work or other activities | | | | | | | | | | | | | | | | | | | 1 | | | | 2 | | |  |
| 1. **Accomplished less** than you would like | | | | | | | | | | | | | | | | | | | 1 | | | | 2 | | |  |
| 19. Didn't do work or other activities as **carefully** as usual | | | | | | | | | | | | | | | | | | | 1 | | | | 2 | | |  |
| 20. During the **past 4 weeks**, to what extent has your physical health or emotional problems interfered with your normal social activities with family, friends, neighbors, or groups? | | | | | Not at all | | | | | Slightly | | | | | Moderately | | | | Quite a bit | | | | Extremely | | |  |
|  |  |  |  |  | 5 | | | | | 4 | | | | | 3 | | | | 2 | | | | 1 | | |  |
| 21.How much **bodily** pain have you had during the **past 4 weeks**? | None | | | Very mild | | | | | | Mild | | | | | Moderate | | | | | Severe | | | | Very severe | |  |
|  | 6 | | | 5.4 | | | | | | 4.2 | | | | | 3.1 | | | | | 2.2 | | | | 1 | |  |
| 22.During the past 4 weeks, how much did pain interfere with your normal work (including both work outside the home and housework)? | | Score selection | | | | | Not at all | | | | A little bit | | | | Moderately | | | | Quite a bit | | | | Extremely | | |  |
|  |  | Answered items 21 | | | | | 6 | | | | 4.75 | | | | 3.5 | | | | 2.25 | | | | 1 | | |  |
|  |  | Unanswered items 21 | | | | | 5 | | | | 4 | | | | 3 | | | | 2 | | | | 1 | | |  |
| These questions are about how you feel and how things have been with you during the past 4 weeks. For each question, please give the one answer that comes closest to the way you have been feeling. How much of the time during the past 4 weeks... | | | | All of the time | | | | | Most of the time | | | | A good bit of the time | | | | Some of the time | | | | A little of the time | | | | None of the time |  |
| 23.Did you feel full of pep? | | | | 6 | | | | | 5 | | | | 4 | | | | 3 | | | | 2 | | | | 1 |  |
| 24.Have you been a very nervous person? | | | | 1 | | | | | 2 | | | | 3 | | | | 4 | | | | 5 | | | | 6 |  |
| 25.Have you felt so down in the dumps that nothing could cheer you up? | | | | 1 | | | | | 2 | | | | 3 | | | | 4 | | | | 5 | | | | 6 |  |
| 26.Have you felt calm and peaceful? | | | | 6 | | | | | 5 | | | | 4 | | | | 3 | | | | 2 | | | | 1 |  |
| 27.Did you have a lot of energy? | | | | 6 | | | | | 5 | | | | 4 | | | | 3 | | | | 2 | | | | 1 |  |
| 28.Have you felt down hearted and blue? | | | | 1 | | | | | 2 | | | | 3 | | | | 4 | | | | 5 | | | | 6 |  |
| 29.Did you feel worn out? | | | | 1 | | | | | 2 | | | | 3 | | | | 4 | | | | 5 | | | | 6 |  |
| 30.Have you been a happy person? | | | | 6 | | | | | 5 | | | | 4 | | | | 3 | | | | 2 | | | | 1 |  |
| 31.Did you feel tired? | | | | 1 | | | | | 2 | | | | 3 | | | | 4 | | | | 5 | | | | 6 |  |
| 32.During the **past 4 weeks**, how much of the time has **your physical health or emotional problems** interfered with your social activities (like visiting with friends, relatives, etc.)? | | | All of the time | | | | | Most of the time | | | | | | Some of the time | | | | | A little of the time | | | | | None of the time | |  |
|  |  |  | 1 | | | | | 2 | | | | | | 3 | | | | | 4 | | | | | 5 | |  |
| How TRUE or FALSE is **each** of the following statements for you. | | | Definitely true | | | | | Mostly true | | | | | | Don't know | | | | | Mostly false | | | | | Definitely false | |  |
| 1. I seem to get sick a little easier than other people | | | 1 | | | | | 2 | | | | | | 3 | | | | | 4 | | | | | 5 | |  |
| 1. I am as healthy as anybody I know | | | 5 | | | | | 4 | | | | | | 3 | | | | | 2 | | | | | 1 | |  |
| 1. I expect my health to get worse | | | 1 | | | | | 2 | | | | | | 3 | | | | | 4 | | | | | 5 | |  |
| 1. My health is excellent | | | 5 | | | | | 4 | | | | | | 3 | | | | | 2 | | | | | 1 | |  |
| Score conversion = (actual score - lowest score in that aspect) / (highest score in that aspect - lowest score in that aspect) × 100; missing entry scores are replaced by the average score of their respective aspect. | | | | | | | | | | | | | | | | | | | conversion formula | | | | | | | Score conversion |
| Physical Functioning(PF), Consist of items 3-12, | | | | | | | | | | | | | | | | | | | PF=(actual score-10）/20×100 | | | | | | |  |
| Role-Physical(RP) ,Consist of items 13-16, | | | | | | | | | | | | | | | | | | | RP=（actual score-4）/4×100 | | | | | | |  |
| Bodily Pain(BP), Consist of items 21, 22, | | | | | | | | | | | | | | | | | | | BP=（actual score-2）/10×100 | | | | | | |  |
| General Health(GH), Consist of items 1, 33-36, | | | | | | | | | | | | | | | | | | | GH=（actual score-5）/20×100 | | | | | | |  |
| Vitality(VT), Consist of items 23, 27, 29, 31, | | | | | | | | | | | | | | | | | | | VT=（actual score-4）/20×100 | | | | | | |  |
| Social Functioning(SF), Consist of items 20, 32, | | | | | | | | | | | | | | | | | | | SF=（actual score-2）/9×100 | | | | | | |  |
| Role-Emotional(RE), Consist of items 17-19, | | | | | | | | | | | | | | | | | | | RE=（actual score-3）/3×100 | | | | | | |  |
| Mental Health(MH), Consist of items 24-26, 28, 30, | | | | | | | | | | | | | | | | | | | MH=（actual score-5）/25×10 | | | | | | |  |
| Reported Health Transition(HT), Consist of items 2, | | | | | | | | | | | | | | | | | | | HT=（actual score-1）/4×100 | | | | | | |  |
| ReferencesWare, J.E., Jr., & Sherbourne, C.D. “The MOS 36-Item Short-Form Health Survey (SF-36): I. Conceptual Framework and Item Selection,”. Medical Care, 30:473-483, 1992.Hays, R.D., & Shapiro, M.F. “An Overview of Generic Health-Related Quality of Life Measures for HIV Research,” Quality of Life Research. 1:91-97, 1992. Steward, A.L., Sherbourne, C., Hayes, R.D., et al. “Summary and Discussion of MOS Measures,” in A.L. Stewart & J.E. Ware (eds.), Measuring Functioning and Well-Being: The Medical Outcome Study Approach (pp. 345-371). Durham, NC: Duke University Press, 1992. | | | | | | | | | | | | | | | | | | | | | | | | | | |

**Appendix 7 Table 6 Randomization Application Form**

Application Date: Year Month Day Random Number:

| **General Information** | | | |
| --- | --- | --- | --- |
| Name Abbreviation: | | Age: years | Gender: Male □ Female □ |
| Weight： Kg | | Height： m | BMI Index： Kg/m2 |
| DMS-5 Diagnosis | Generalized Anxiety Disorder Severity: Mild □, Moderate □, Severe □ | | |
|  | Severity of Major Depression: Mild □, Moderate □, Severe □ | | |

| Selection Criteria Checklist | | |
| --- | --- | --- |
| Inclusion criteria | Yes | No |
| 1.Meeting the CPPS diagnostic criteria in the 2022 EAU guidelines | □ | □ |
| 2. Definitive generalized anxiety disorder or major depressive disorder | □ | □ |
| 3. Individuals aged between 18 and 70 years | □ | □ |
| 4.No identifiable pathological changes in physical examinations and auxiliary tests | □ | □ |
| 5.No treatment other than oral medications in the 3 months prior to the visit | □ | □ |
| 6.Patient's informed consent and voluntary participation in the study | □ | □ |
| All of the above criteria must be "yes," otherwise the patient cannot be enrolled. | | |

| Exclusion Criteria Checklist | | |
| --- | --- | --- |
| Exclusion criteria | Yes | No |
| 1. Patients in the acute phase of systemic and intracranial hemorrhagic diseases | □ | □ |
| 2.Individuals with serious underlying conditions, such as cardiovascular, liver, kidney, respiratory, and blood disorders, in addition to malignant tumors and other advancing illnesses | □ | □ |
| 3.Patients with cardiac metal membranes, cardiac pacemakers, intracranial metal implants, lumbar sacral metal implants, and implantable electronic devices | □ | □ |
| 4.Individuals with infections in the head or lumbar sacral regions | □ | □ |
| 5.Individuals exhibiting unstable vital signs | □ | □ |
| 6.Patients with previous adverse reactions to magnetic therapy | □ | □ |
| 7.Individuals with atypical autonomic reflexes | □ | □ |
| 8. Patients with cognitive impairment who cannot cooperate | □ | □ |
| 9. Expectant or breastfeeding women | □ | □ |
| 10. Patients with a history diseases causing peripheral nerve damage | □ | □ |
| 11. Patients with debilitating diseases, such as malignant effusion, active pulmonary tuberculosis, cancer, or myasthenia gravis | □ | □ |
| 12.Patients with severe mental illness or epilepsy | □ | □ |
| All of the above criteria must be "no"; otherwise, the patient cannot be enrolled. | | |

Is it eligible for enrollment: Yes □ No □

Reason for not enrolling:

Randomization results for eligible participants:

Group A (dual-target magnetic stimulation) □

Group B (peripheral magnetic stimulation) □

Group C (sham stimulation) □

**Thank you for your participation!**
