# Supplementary material for: Dual-target peripheral and central magnetic stimulation for rehabilitation of chronic pelvic pain syndrome associated with psychosomatic symptoms: Study protocol for a randomized controlled trial
Source: PLoS One. 2025 Jul 17;20(7):e0326740. doi: 10.1371/journal.pone.0326740 (PMC12270166; doi:10.1371/journal.pone.0326740)
Supplement: Attachment — Submitted filename: renamed_b9c25.docx [file pone.0326740.s005.docx]

# COMMENTS – Manuscript PONE-D-24-31808

Title: “Peripheral combined central dual-target magnetic stimulation for rehabilitation of chronic pelvic pain syndrome with psychosomatic disorders: Study protocol for a randomized controlled trial”

**Important note: This review pertains only to ‘statistical aspects’ of the study and so ‘clinical aspects’ [like medical importance, relevance of the study, ‘clinical significance and** **implication(s)’ of the whole study, etc.] are to be evaluated [should be assessed] separately/independently. Further please note that any ‘statistical review’ is generally done under the assumption that study specific methodological [as well as execution] issues are perfectly taken care of by the investigator(s). This review is not an exception to that and so does not cover clinical aspects {however, seldom comments are made only if those issues are intimately / scientifically related & intermingle with ‘statistical aspects’ of the study}. Agreed that** **‘statistical methods’ are used as just tools here, however, they are vital part of methodology [and so should be given due importance]. I look at the manuscript in/with statistical view point, other reviewer(s) look(s) at it with different angle so that in totality the review is very comprehensive. However, there should be efforts from authors side to improve (may be by taking clues from reviewer’s comments). Therefore, please do not limit the revision only (with respect) to comments made here.**

**COMMENTS:** Unfortunately, there are [as I see/found] many minors as well as few major flaws in the study/manuscript. I have different opinion / observations/concerns or rather questions regarding few issues [including serious ones] which [only vital ones] are given below:

Information given in section ‘Sample size’ (lines 197-202) is not adequate. You assumed the effect size (of 0.5) and quoted an article which is not directly or indirectly(?) related to your topic {according lines 41-43 “This study will aim to assess the efficacy and safety of dual-target magnetic stimulation in CPPS patients with psychological disorders” whereas that article is “Sacral nerve magnetic stimulation combined with extracorporeal shockwave for the treatment of type-ⅢB chronic prostatitis”. Is it correct to use ‘effect size’ from a study in which only the term ‘magnetic stimulation’ is used? Further you said “The pain visual analog score will be used as the primary measurement standard” in which case the sample size has be inflated at least by 10% as the variable is “ordinal”. Software “G*Power” is excellent but the final result [required sample size for this study] will be according to “input”. Since most of the software will yield the result (number of subjects required per arm), I request authors to confirm this [if 54 patients required per arm?].

In this context, I request to kindly note that according to table-2 on page 158 of Jacob Cohen’s paper “A power primer” in Psychological Bulletin, 1992, vol.:112, pp 155-159 [which is a sort of summary of the excellent book by Cohen himself titled ‘Statistical power analysis for the behavioral sciences’, Academic Press, 1977, New York] for medium effect size you need n=64 [for two groups study] or =52 [for three groups study] per group (type-I error=0.05, power=80%). Please note that the ‘effect size’ assumed should have some basis (exact reference needs to be quoted) &/or reasonable/realistic, else the study is very likely ‘not to be able to’ detect a difference despite its presence}.

As stated in the aim {lines 41-43 “This study will aim to assess the efficacy and safety of dual-target magnetic stimulation in CPPS patients with psychological disorders”} that you are assuming existence of some psychological disorders, then why are assessing for presence of psychological disorders {refer to lines 59-60: secondary outcomes will include the Depression, Anxiety and Stress Scale (DASS-21)}. Is not contradictory? In ‘Abstract-Methods’ it is stated that “This prospective, double-blind, randomized controlled trial will recruit 60 CPPS participants and randomly assigned them to three groups (1:1:1) following gender stratification” then in section ‘Study population’ [lines 139-140] it is said that “The study population has no gender restrictions and will include individuals aged 18–70 years, experiencing CPPS accompanied by anxiety or depressions”. Which one is valid or you intend to convey something else? You may be correct, but the question is: How you will execute the recruitment process? [In fact, “How you are executing the recruitment process?” because according section on ‘Trial status’ (lines 403-5) “The trial is currently in the recruitment phase, which began in January 2024 and will end in December 2025. It is still ongoing, and the research is working hard to reach the necessary sample size”.

The difference(s) in [if any] between Repetitive peripheral magnetic stimulation (rPMS) and repetitive transcranial magnetic stimulation (rTMS) has/have not given/brought out/discussed. As stated, “This study will aim to assess the efficacy and safety of dual-target magnetic stimulation in CPPS patients with psychological disorders”, I guess, by “dual-target magnetic stimulation” you mean “rPMS + rTMS. If that is true, the question is ‘Why there are three groups: dual-target magnetic stimulation, peripheral magnetic stimulation, and sham stimulation’? What is this ‘standard treatment’ which all are receiving? That means ‘sham stimulation’ group is ‘active control’ group. Why not work with only two groups: dual-target magnetic stimulation versus/and sham stimulation? What is the logic of having ‘peripheral magnetic stimulation’ group (separate third group)? In short, ‘DESIGN OF THE STUDY’ (one of the very vital/important issue) is not very clear.

In the ‘ABSTRACT’ (lines 41-43) it is stated that “. This study will aim to assess the efficacy and safety of dual-target magnetic stimulation in CPPS patients with psychological disorders” then why you state in lines 107-8:: The aim of this study is to design a high-quality RCT with the stratified randomization of participants? . Question is then ‘which is correct?’ In lines 294-6 you said “A third researcher will …. and distribute the envelopes to the participating patients”. The question is ‘why distribute the envelopes to the participating patients? Is what is described under section “Blinding” [lines 285-308] is really a BLINDING procedure? In fact, at many places section heading is different than the contents [example, section “Randomization and allocation concealment” (lines 213-219)]. In ‘Abstract-Methods’ (lines 44-46) it is stated that “This prospective, double-blind, randomized controlled trial will recruit 60 CPPS participants and randomly assigned them to three groups (1:1:1) following gender stratification“ but the term ‘gender stratification’ does/did not appear anywhere else. Similarly, the term ‘stratified randomization’ was used only in line 107. Regrettably, it seems that ‘gender stratification was forgotten then [as it never reflected later].

Surprising (highly regret to note that) one important term “CONSORT” is not mentioned anywhere in this manuscript. It is well-known that while reporting {findings from and even planning [let the intervention techniques be therapeutic agents, devices, regimes or procedures (i.e. therapeutic trials), preventive ones like vaccines (i.e. prophylactic trials) or rehabilitative, educational etc]}] ‘Clinical Trial’ one should follow CONSORT guidelines. Even important items {like Random Sequence generation (Item 8a), Allocation concealment (Item 9), Blinding (Item 11a)} of/in CONSORT checklist are not described properly [since your article type is ‘Clinical Trial’, you are supposed to cover these items in the report or even in ‘Protocol’ (even if you may not use them) correctly/adquately].

Very good that ‘SPIRIT’ Checklist’ is given in supplementary information file (as S2). However, the accuracy/correctness of the page numbers indicated to content that info seems to be doubtful [example: for item 14 on ‘Sample Size’ you indicated/referred to page 8 but any relevant info is not found there]. Similarly, note that Figure 2: Clinical trial flow chart, similar diagram of flow of sample size is suggested in CONSORT guidelines {available for free download on WWW/NET}.

In “Intervention” section (line 220 onwards) it is stated that “Clinical intervention measures and steps. 1) Before treatment: conduct three checks and seven comparisons…..” and subsequently many things are described. Question is: ‘Are they comparisons (as stated) ‘? If not what they are? Please clarify. Kindly check for the ‘English’ language {this is only as an example; the entire manuscript is full of such examples. One such is in lines 404-5 (the research is working hard to reach the necessary sample size)}. Agreed that English is not our mother tongue (definitely not mine, may or may not be yours but certainly not of many readers), however in any case, remember/ Kindly mind you [please excuse me for such a harsh comment/statement] that this is a scientific/academic document and so all details should be clearly/correctly communicated (do not take reader’s for granted). You may take help of language professional expert, if needed.

Section on ‘Statistical analyses’ (lines 370-76) is extremely ‘inadequate’ (utterly failed to mention even most important/vital techniques). No test is proposed for between groups comparison. Moreover, you stated that “Continuous data will be presented as mean ± standard deviation” but note that all continuous appearing variables may not have/achieved a ‘ratio’ level of measurement. Then Paired t-tests will not be applicable. In this context, I request authors to read the following note pasted from one standard textbook on ‘Medical Research Methodology’ [though I am sure that the authors already know these things].

Though few variables are continuous in appearance they are likely to yield data that are in [at the most] ‘ordinal’ level of measurement [and not in ratio level of measurement for sure]. Then application of suitable non-parametric (or distribution free) test(s) is/are indicated/advisable [even if distribution may be ‘Gaussian’ (also called ‘normal’)]. Agreed that there is/are no non-parametric test(s)/technique(s) available to be used as alternative in all situation(s), but should be used whenever/wherever they are available. Therefore, in short use suitable non-parametric test(s)/technique(s) while dealing with data that are in ‘ordinal’ level of measurement even if [despite that] the distribution may be ‘Gaussian’.

Note that though the measures/tools used are appropriate often times [examples: Pelvic pain score sheet (for females), NIH-CPSI (for males), Anxiety and depression score (DASS-21), SF-36 QoL scale], most of them are likely to yield data that are in ‘ordinal’ level of measurement.

Look at the ‘Conclusions’ [To our knowledge, there have been no literature reports on randomized controlled trials evaluating the impact of dual-target magnetic stimulation on CPPS with comorbid psychiatric disorders. It may serve as a valuable reference for future clinical interventions and research on CPPS with comorbid psychiatric disorders.]. Are they conclusions of/from this study? Basically, ‘are you supposed to draw any ‘Conclusions’ in protocol?’

Moreover, limitations (if any) of the study are not mentioned/listed anywhere. Does that mean {according to authors} there are none? As pointed out in ‘important note’ above “This review pertains only to ‘statistical aspects’ of the study and so ‘clinical aspects’ should be assessed separately/independently [one should carefully consider/look at the clinical implications of the study]. In my opinion, to rescue this article (which seems quite difficult, if not impossible), large amount of re-vision (re-drafting) may be needed. However, please do not limit the revision only (with respect) to comments made here. More improvement is expected. Nevertheless, ‘how to handle/accommodate these suggestions is not questionable as the study is in protocol stage. The respected ‘Editor’ may consider accepting/further processing only if found ‘clinical implications’ valuable [i.e., add(s) to clinical knowledge or positively influence clinical practice]. ‘Major revision’ is recommended [in lieu of plain rejection, to give chance to authors for improvement of the manuscript].
