# Supplementary material for: Dual-target peripheral and central magnetic stimulation for rehabilitation of chronic pelvic pain syndrome associated with psychosomatic symptoms: Study protocol for a randomized controlled trial
Source: PLoS One. 2025 Jul 17;20(7):e0326740. doi: 10.1371/journal.pone.0326740 (PMC12270166; doi:10.1371/journal.pone.0326740)
Supplement: Attachment — Submitted filename: Response_to_Reviewers_auresp_2.docx [file pone.0326740.s008.docx]

Dear Editors and Reviewers:

Thank you for your letter and for the reviewers’ comments concerning our manuscript entitled “Peripheral combined central dual-target magnetic stimulation for rehabilitation of chronic pelvic pain syndrome with psychosomatic disorders: Study protocol for a randomized controlled trial”(ID: PONE-D-24-31808R1). Those comments are all valuable and very helpful for revising and improving our paper, as well as the important guiding significance to our researches. We have studied comments carefully and have made correction which we hope meet with approval.

Previously revised parts are still marked in yellow in the thesis, and new revised parts are standardized in green. The main corrections in the paper and the responds to the reviewer’s comments are as flowing:

**Reviewer #1**: I am happy to see that all most all the comments made on earlier draft are/were addressed/answered. Few are even considered positively & are attended {however not all reasons/arguments are very convincing – though the answers are very comprehensive many are not to the point / focused]. Nevertheless, I recommend the acceptance (yet the editor can decide ‘not to accept’ as original manuscript level/quality is difficult to improve a lot especially if the study is already started which is the case here) only because for revised version much hard work is done by the authors. As a result, the manuscript in present form is ready to publish [with few quality limitations ofcourse], however, following point(s) is/are to be noted and please be incorporated.

**Response to comment:** Thank you again for your positive comments and valuable suggestions to improve the quality of our manuscript.

**Reviewer #**1: According to revised manuscript (lines 122-3: Recruitment will continue until 33 male and 33 female recruits have been registered.) and in lines 119-120 you stated that “Eligible patients, upon providing informed consent, will be categorized by gender and subsequently randomized in a 1:1:1 ratio into the following three groups” is little confusing. Process/method/procedure to be adapted for ‘randomized allocation’ need(s) to be clear. Sample size in each group is 20 i.e. total is 60. But according to lines 122-123 total sample size is 66 [33 male and 33 female recruits]. What is it? How you have achieved (or will achieve) the same sample size [20 or 22 in each group] without using ‘Permuted Block Randomization’ technique? {because there is no such mention}.
Assuming that sample size fixed as 66, you need to give details about how ‘you are going ensure that 11 males and 11 females in each group’ are allocated. In my opinion, sample size fixed at 24 in each group [therefore total sample size is fixed as 72] is more suitable as 24 is completely divisible by randomly chosen permuted block size {3,6} and 2 {M, F}. But even in this case, ‘allocation’ is complicated and can this be called as purely random (or sort of ‘quota’)?

**Response to comment:** Thank you for your comments on our article. According to your suggestions, for inclusion in the study, we used stratification by gender followed by block randomization of participants into 3 groups on a 1:1:1 basis. The description of the modifications has been marked in green in the text and is detailed in lines 65-76 and 120-123. In addition, we also changed the flowchart to have 22 participants in each group.

We appreciate for Editors/Reviewers’ warm work earnestly, and hope the correction will meet with approval. Once again, thank you very much for your comments and suggestions.

Best regards,

Sincerely

Xiangdong Yang

Department of Anorectal, Chengdu Anorectal Hospital, Chengdu, Sichuan Province, China.

Add: 152 Daqiang East Street, Taisheng South Road, Qingyang District, Chengdu City, Sichuan Province, China

Email: xiangdongyang2011@163.com
