# Supplementary material for: Dual-target peripheral and central magnetic stimulation for rehabilitation of chronic pelvic pain syndrome associated with psychosomatic symptoms: Study protocol for a randomized controlled trial
Source: PLoS One. 2025 Jul 17;20(7):e0326740. doi: 10.1371/journal.pone.0326740 (PMC12270166; doi:10.1371/journal.pone.0326740)
Supplement: Attachment — Submitted filename: Response_to_Reviewers_auresp_3.docx [file pone.0326740.s009.docx]

Dear Editor and Reviewers,

Thank you for your letter and the reviewers' comments on our paper entitled “Peripheral combined central dual-target magnetic stimulation for rehabilitation of chronic pelvic pain syndrome with psychosomatic disorders: study protocol for a randomized controlled trial” (ID: PONE-D-24-31808R1). All comments are valuable and have been very helpful in revising and improving our manuscript. We have carefully studied all comments and revised the manuscript accordingly, and hope the revised version meets with your approval.

Changes made in response to the reviewers' comments are highlighted in yellow throughout the revised manuscript. The major revisions and our responses to the reviewers' comments are as follows:

Reviewer #1:

Comment 1: Although most of the comments made on the previous manuscript were addressed/answered, I am not very happy with this manuscript [because of the new mistakes/errors]. I don't find the assignment instructions given very convincing - how do you ensure gender balance in each group? Please clarify.

Comment 2: The reviewer was also not very happy with some other issues [e.g. sample size]. The presentation is lengthy, but sentences/materials are confusing or use non-standard English.

Response:

We sincerely appreciate the reviewer's continued scrutiny and valuable suggestions for improving our manuscript.

Response to Comment 1 (Gender Balance): We addressed gender balance by employing stratified randomization. Based on the known gender incidence ratio (male:female = 2:3) of CPPS with psychosomatic disorders at our center, participants were randomized into the three study groups with stratification by gender. The relevant text has been highlighted in yellow in the revised manuscript (Lines 150, 223-232, 244-245). Additionally, we have updated the study flowchart (Figure 3) to clearly reflect the allocation of 25 participants per group.

Response to Comment 2 (Other Issues & Presentation): We thank the reviewer for highlighting these concerns. We have carefully reviewed the sample size justification and presentation throughout the manuscript. The sample size calculation section has been reviewed for clarity. We have also thoroughly edited the entire manuscript to improve conciseness, sentence structure, clarity, and standard English usage. Non-essential details have been removed where possible.

We sincerely thank the Editors and Reviewers for their time and insightful feedback. We hope that the revisions made in response to the comments are satisfactory. Thank you again for your comments and suggestions.

Best regards,

Sincerely

Xiangdong Yang

Department of Anorectal, Chengdu Anorectal Hospital, Chengdu, Sichuan Province, China.

Add: 152 Daqiang East Street, Taisheng South Road, Qingyang District, Chengdu City, Sichuan Province, China

Email: xiangdongyang2011@163.com
